# Mono- and Bichromophores Formed from Perylene Monoimide Diesters: Competition between Intramolecular Charge Transfer and Intermolecular Singlet Exciton Fission

**DOI:** 10.1021/acs.jpca.4c05424

**Published:** 2024-10-23

**Authors:** Demet Demirci Gültekin, Serkan Şen, Ayhan Elmalı, Ahmet Karatay, Muhammet Erkan Köse, Anthony Harriman, Özgür Altan Bozdemir

**Affiliations:** † Department of Chemistry and Chemical Process Technologies, Technical Sciences Vocational School, Ataturk University, Erzurum 25240, Turkey; ‡ Department of Chemistry, Faculty of Science, Ordu University, Ordu 52200, Turkey; § Department of Physics Engineering, Ankara University, Ankara 06100, Turkey; ∥ Department of Chemistry, Kocaeli University, Izmit, Kocaeli 41001, Turkey; ⊥ Molecular Photonics Laboratory, SNES, Newcastle University, Newcastle upon Tyne NE1 7RU, U.K.; # Department of Chemistry, Atatürk University, Erzurum 25240, Turkey

## Abstract

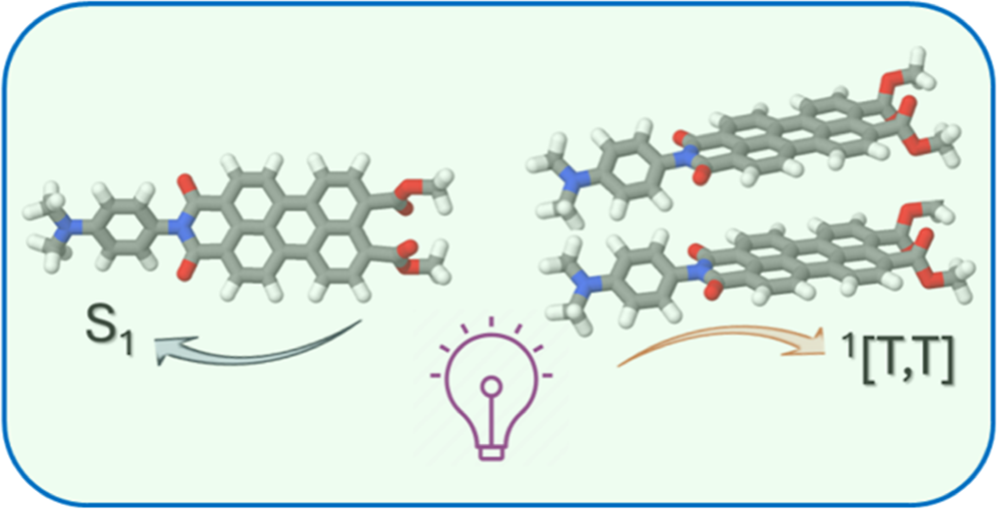

Perylene monoimide diesters and the corresponding phenyl-linked bichromophores are strongly fluorescent in dilute solution with minimal triplet population after relaxation of the initial Franck–Condon state. The monomer forms nonemissive face-to-face dimers in solution, wherein illumination leads to formation of a spin-correlated, triplet pair with a yield of *ca*. 13% and with a time constant of 4 ± 1 ps. The triplet pair, which is localized on the aggregate, cannot separate and decays with a mean lifetime of 80 ± 10 ps. The relaxed S_1_ state of the weakly coupled, phenyl-linked bichromophores establishes an equilibrium with an intramolecular charge-transfer state over a hundred picoseconds or so, depending on the solvent and the geometry of the linkage. This equilibrium mixture, being dominated by the relaxed S_1_ state, decays on the nanosecond time scale in solution at room temperature without implication of a triplet state. Self-association occurs at higher concentration and, for the *para*-bridged bichromophore, leads to inefficient triplet formation in tetrahydrofuran at room temperature.

## Introduction

The 21st century has brought renewed interest in enhancing triplet yields by way of singlet exciton fission (SEF).^1^ Initial attention to this concept, first evoked in the mid-1960s,^2^ arose because of its possible capability to improve the performance of certain types of organic solar cells by increasing carrier density^3^ but such optimism has largely waned in recent years. Nonetheless, interest in the SEF process,^4^ and the mechanistic details in particular,^5^ remain undiminished and there continues to be a remarkably diverse output in fundamental research related to this topic. In passing, the consideration given to SEF has rekindled interest in triplet-singlet electronic energy transfer,^6^ this being a largely ignored feature of molecular photophysics.

The two basic requisites for effective SEF, recognized from the origin of the subject,^7,8^ have not been challenged in contemporary research. These concern the need for the triplet excitation energy not to significantly exceed one-half of the corresponding singlet excitation energy, although a modest degree of endothermicity can be tolerated.^9^ This means that for chromophores absorbing in the orange region, the triplet excitation energy must be less than 1.0 eV. The second requirement is that two or more chromophores must act cooperatively. The latter requisite has led to the study of SEF in crystals,^10,11^ thin films,^12,13^ sublimed deposits,^14,15^ molecular aggregates,^16^ and highly concentrated solutions.^17^ In particular cases, the yield of the triplet state can approach twice that of the precursor excited-singlet state, while the triplet lifetime can reach several microseconds.

Of the dozen or more chromophores that have been used successfully for SEF, our attention was drawn to the perylene monoimides (PMIs).^18,19^ Recent work by Lin et al.^20^ has recorded high triplet yields for thin films and crystals of a sterically blocked derivative, whereas the isolated monomer in solution shows little tendency toward intersystem crossing. Separately, Papadopoulos et al.^21^ reported inefficient triplet formation via intramolecular SEF in solution at room temperature for a bichromophoric 2,7-naphthyl-bridged perylene monoimide diester (PMIDE). The resultant triplet biexciton was subject to geminate triplet–triplet recombination to reform the excited-singlet state, and this situation is clearly disadvantageous for use with organic solar cells. This initial study has now been complemented by a detailed comparison of perylene mono- and diimide-based bichromophores linked via phenyl or naphthyl spacers, where the importance of rotational freedom as a means to optimize SEF was stressed.^22^

In the recent past, it has been recognized that small conjugated molecules can replace fullerenes as electron acceptors in certain types of organic solar cells.^23^ These alternatives include several derivatives of PMIs, and among these have been some bichromophores based on a range of aryl linkers.^24,25^ Such compounds are photochemically stable, possess strong absorption in the visible region, and are open to synthetic modification. Of course, these materials are susceptible to self-association in solution^26^ but improved solubility can be attained with the corresponding diesters. Advanced applications for PMI derivatives requires a thorough knowledge of their photophysical properties,^18,19^ including any tendency for SEF. As part of an ongoing investigation into how stepwise accretion affects the optical properties of PMI diesters,^27^ we report on the photophysics of a few derivatives, including a bichromophore structurally similar to one studied by Papadopoulos et al.^22^

## Methods

Full experimental details, including sample preparation and characterization, are provided in the Supporting Information. All geometry optimizations and frequency calculations were carried out for the target compound in vacuo using the B3LYP hybrid functional^28^ along with the 6-311G(d,p) basis set.^29^ The absence of imaginary frequencies for the optimized geometries was used to confirm the presence of minima on the potential energy surfaces. Time-dependent density functional theory calculations for singlet states were performed with the B3LYP functional, whereas the CAM-B3LYP functional was exploited to determine the triplet energies for B3LYP-optimized geometries.^30^ CAM-B3LYP was chosen to calculate the triplet states since a similar approach for PMI derivatives was used successfully in recent studies.^21,22^ The concentration dependence for optical absorption spectroscopy was made by usual methods,^27^ with analysis according to Equations S3–S5.^31^ The ultrafast transient absorption spectral studies are described in the Supporting Information. The temporal resolution was *ca*. 200 fs. Data interpretation, making use of global analysis, and corrections for spectral chirp and removal of excitation spikes was made by Surface Xplorer.^32^

## Results and Discussion

### Sample Characterization

**Figure 1:**
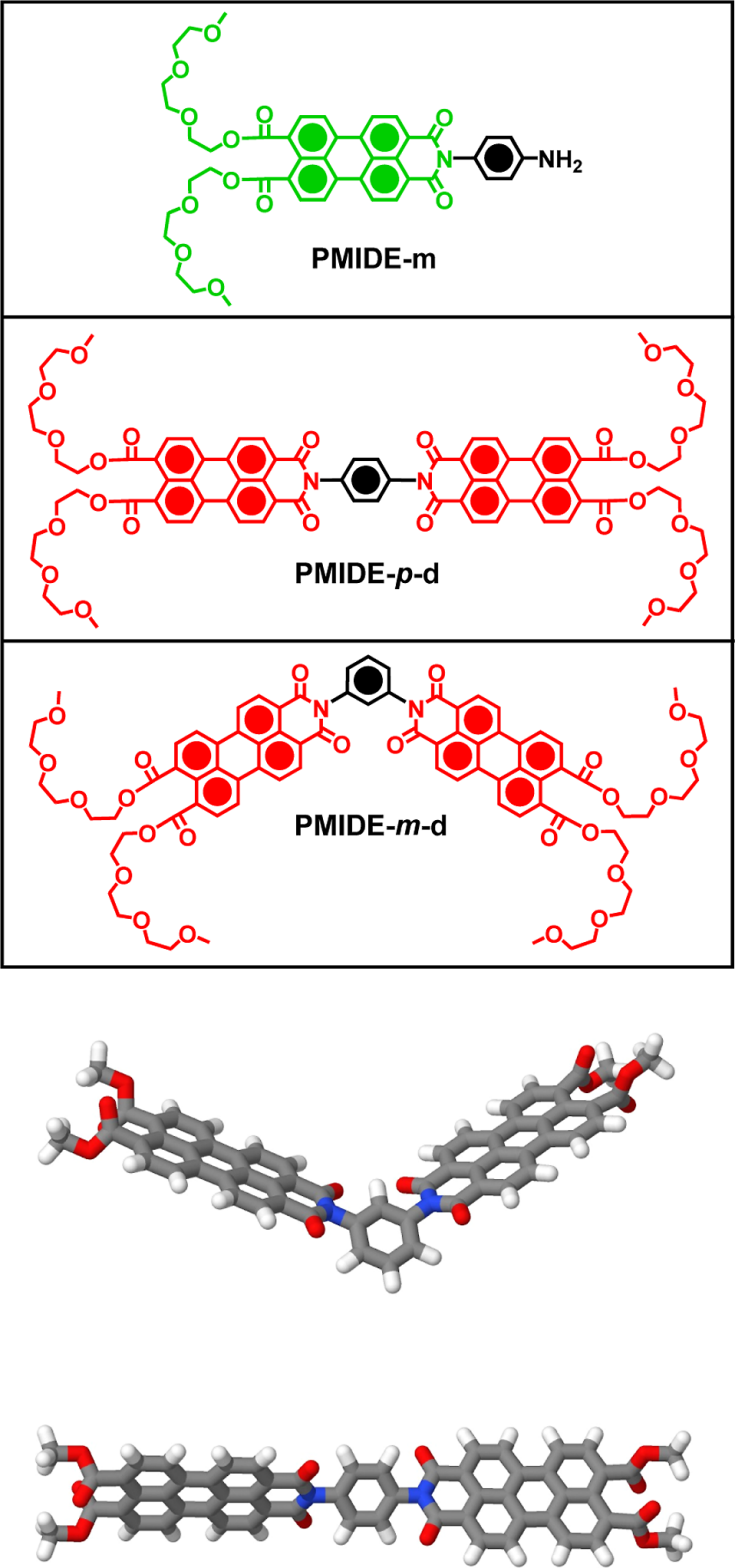
Molecular formulas for the target compounds and computed 3D representations of the two bichromophores.

The target PMIDE derivatives (Figure 1) were synthesized using previously reported^27^ procedures in an effort to compare properties of the monomer, **PMIDE**-**m**, with those of the covalently linked bichromophores, **PMIDE**-*m*-**d** and **PMIDE**-*p*-**d**. These latter compounds differ in terms of the mutual orientation of the two PMIDE units; **PMIDE**-*m*-**d** is a *meta*-dimer with a 120° angle between the PMIDE units, while **PMIDE**-*p*-**d** is a linear *para*-dimer. Consequently, the substitution pattern at the central phenyl spacer determines the overall geometry of the respective bichromophore, although rotation is possible around the connecting bonds. As this linker is attached at the nodal plane of the perylene skeleton, it serves only as a constitutional bridge and does not take part in ground- or excited-state electronic processes associated with the PMIDE chromophores (vide infra). The two tri(ethylene oxide) monomethyl ether chains, appended as sterically demanding carboxyester groups, impart improved solubility in both acetonitrile (MeCN) and tetrahydrofuran (THF).

Optimized ground-state geometries calculated for **PMIDE**-*m*-**d** and **PMIDE**-*p*-**d** show that the central phenyl rings do not lie on the same plane as the appended PMIDE units (Figure 1). These calculations indicate mean torsion angles between the central phenyl ring and the attached polycycles of 64° for **PMIDE**-*m*-**d** and 69° for **PMIDE**-*p*-**d**. These relatively large twist angles help break conjugation between the terminal chromophores and curtail π-electron delocalization throughout the extended molecule. Although such delocalization is possible with the *para*-linkage, the large twist angle serves to minimize electronic communication between the complementary PMIDE units. Indeed, frontier molecular orbital plots for the S_1_ and S_2_ states of **PMIDE**-*m*-**d** and **PMIDE**-*p*-**d** indicate the absence of molecular orbital coefficients associated with the phenyl spacers (Figure S27). As such, each PMIDE functions independently.

The computed S_0_ → S_1_ absorption transition energy for **PMIDE**-**m** is 2.49 eV, which is in excellent agreement with the experimental value of 2.48 eV. The calculated S_0_ → S_1_ absorption energy for both **PMIDE**-*m*-**d** and **PMIDE**-*p*-**d** is 2.45 eV, again in close agreement to the experimental values of 2.49 and 2.50 eV, respectively. These values overestimate the excitation energy of the relaxed S_1_ state. The T_1_ energy measured^21,22^ for a PMIDE bichromophore bridged by a 2,7-naphthyl linker was reported to be 1.12 eV, and we anticipate a similar value for our compounds, although we were unable to observe low-temperature phosphorescence. The calculated T_1_ energies are in the range of 1.1 ± 0.1 eV (Table S1), in full agreement with literature values.^20−22,33^ The derived natural transition orbitals, generated from the canonical molecular orbitals for vertical transitions of the triplet-excited states, indicate that electronic transitions are localized on the PMIDE units in each bichromophore (Figure S28 and p S29). This finding emphasizes the electronic isolation of each PMIDE within the bichromophore.

### Optical Spectroscopic Studies with **PMIDE-m**

**Figure 2:**
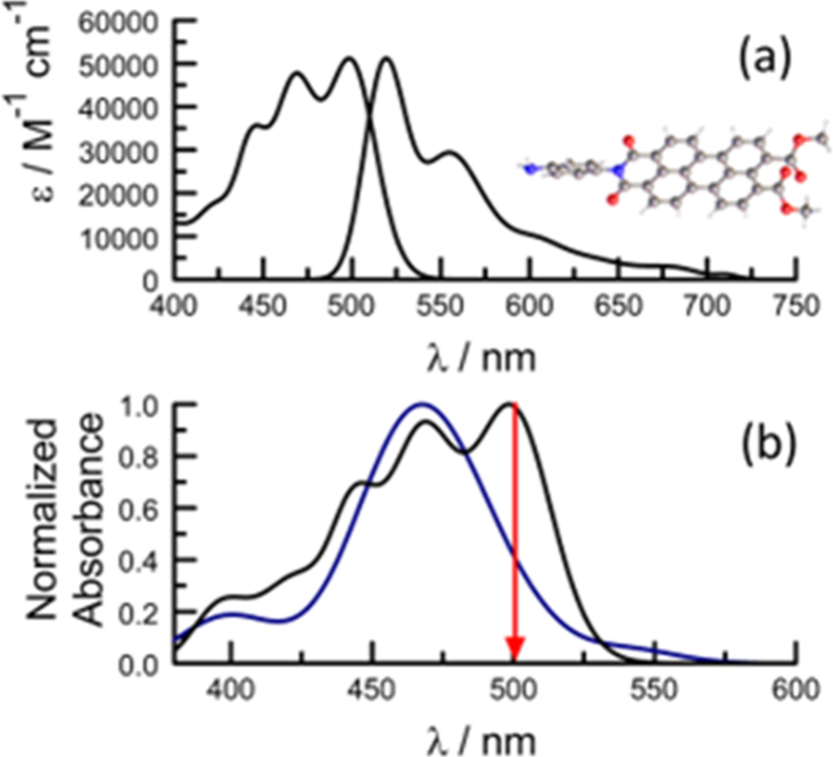
(a) Absorption and normalized fluorescence spectra for **PMIDE**-**m** in THF solution at a concentration of 2 × 10^–7^ M, together with the computed molecular geometry. (b) Comparison of normalized absorption spectra for monomeric (2 × 10^–7^ M; black curve) and aggregated (2 × 10^–4^ M; violet curve) forms of **PMIDE**-**m** in THF. The arrow shows the excitation wavelength used for spectroscopic studies.

For **PMIDE**-**m** in THF, the 0,0 transitions for absorption and fluorescence are located at 500 nm (i.e., 2.48 eV) and 520 nm (i.e., 2.38 eV), respectively. This amounts to a sizable Stokes shift of 770 cm^–1^, indicating a modest geometry change following excitation. Similar values are found for MeCN solutions (Table 1). The molar absorption coefficient, ε_MAX_, at the absorption maximum was determined to be 51,200 M^–1^ cm^–1^ in dilute THF solution. The fluorescence quantum yield (ϕ_F_) in MeCN at room temperature is 0.66 ± 0.04, while the excited-singlet state lifetime (τ_S_), measured by time-correlated, single photon counting, is 5.2 ± 0.1 ns; analysis of the decay profiles indicated the presence of a minor (i.e., <4%) component with a slightly longer lifetime in both solvents (Figure S43). These parameters are consistent with literature values for related compounds.^34^ There is superficial mirror symmetry between normalized absorption and fluorescence spectra (Figure 2a), which can be traced to differences in the underlying vibronic envelope. Thus, while the reduced absorption spectrum can be deconstructed into a progression of medium-frequency vibrations of 1280 cm^–1^, it is necessary to include a second vibration of *ca*. 700 cm^–1^ to properly describe the reduced emission spectrum (Figure S4).

**Table 1:** Collation of the Photophysical and Spectroscopic Properties Derived for PMIDE-m in Dilute Solution at Room Temperature

property	MeCN	THF
ν_ABS_/cm^–1^^a^	20,050	20,000
ν_FLU_/cm^–1^^b^	19,165	19,230
Δ_SS_/cm^–1^^c^	885	770
ε_MAX_/M^–1^ cm^–1^	NA	51,200
φ_F_	0.66	0.70
τ_S_/ns	5.2	5.4
τ_T_/μs^d^	45	NA
τ_R_/ps	2.7	2.8
τ_S’_/ns^e^	4.7	3.9
K_2_/M^–1^	1.6 × 10^3^	1.1 × 10^4^

a 0,0 transition for absorption.

b 0,0 transition for fluorescence.

c Stokes shift using 0,0 transitions.

d Triplet lifetime in deoxygenated solution.

e Lifetime for relaxed S_1_ state measured by transient absorption spectroscopy.

**Figure 3:**
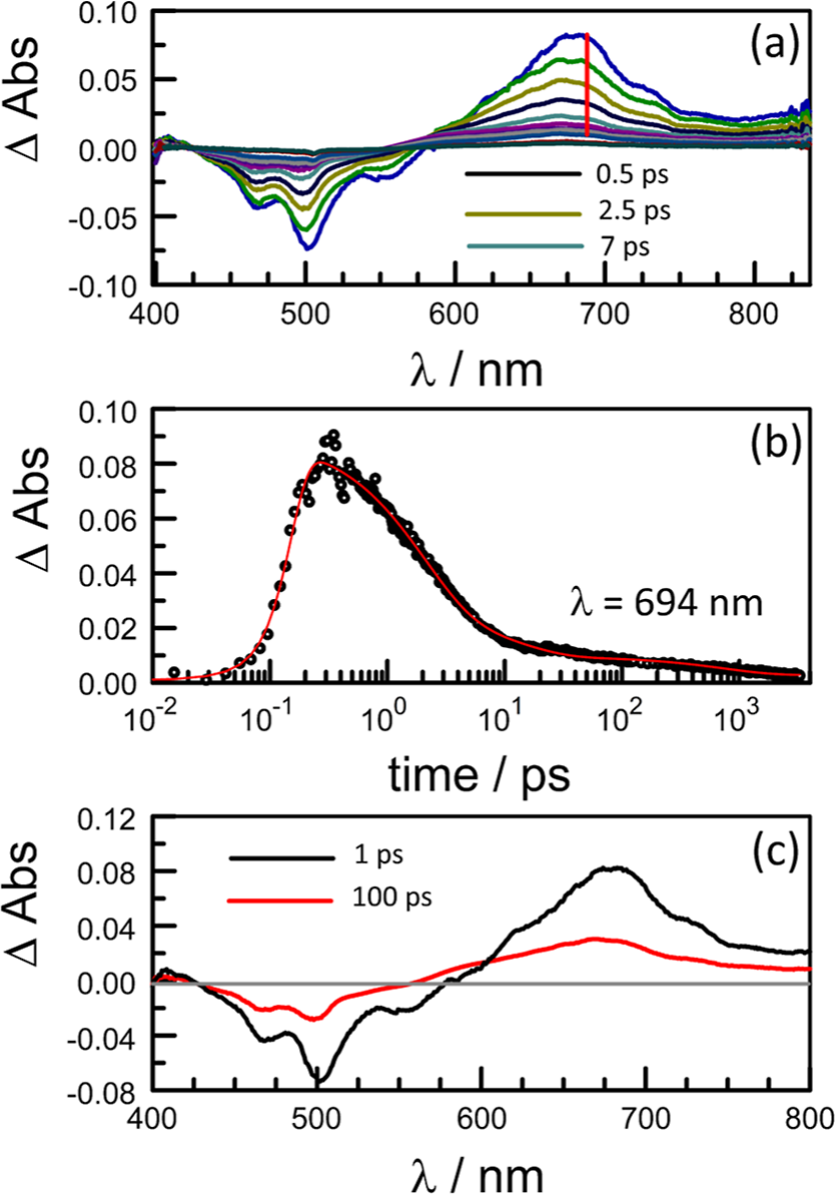
(a) Examples of transient differential absorption spectra recorded following excitation of **PMIDE**-**m** in MeCN (*C*_T_ = 100 μM; α_D_ = 0.13; *F*_D_ = 4%) with a 120 fs laser pulse at 500 nm. Spectra were recorded at delay times of 0.5, 1.25, 2.5, 4, 7, 12, 20, 40, 100, 2000, and 4000 ps; see Figure S32a for full color coding. The arrow indicates the direction of increasing delay time. (b) Typical decay trace (open circles) and kinetic fit (red curve) to the lifetimes reported in the text. (c) Derived differential absorption spectra for S_FC_ (1 ps; black curve) and the relaxed S_1_ (100 ps; red curve) obtained from the species associated difference spectra (SADS) analysis. See Figure S33 for further examples of kinetic traces.

Prior work by Papadopoulos et al.^22^ has established that the ultrafast transient absorption spectral records for a derivative similar to **PMIDE**-**m** in 2-methyltetrahydrofuran are consistent with a two-state model. Thus, excitation produces a Franck–Condon state (S_FC_) that undergoes solvent-assisted relaxation to form the fluorescent excited-singlet state (S_1_). The latter decays over a few nanoseconds without significant population of the triplet state. Our results with dilute solutions of **PMIDE**-**m** are consistent with these prior studies;^22^ the total concentration of chromophore (C_T_), the mole fraction of dimer (α_D_), and the percentage of incident light absorbed by the dimer (F_D_) are indicated in the caption to Figure 3.

Thus, fs-laser excitation of **PMIDE-m** in MeCN at 500 nm resulted in a strong negative signal with a minimum at 506 nm; this signal comprises depletion of the ground-state absorption bands centered at 475 and 500 nm together with a crop of stimulated emission having a peak intensity at 520 nm (Figure 3). In addition, there is a broad absorption band covering the range from 580 to 800 nm, with a maximum at 690 nm. This species is formed within the excitation pulse and therefore can be assigned to S_FC_. Fast vibrational relaxation to the thermally equilibrated S_1_ state occurs with a time constant (τ_R_) of 2.7 ± 0.3 ps in MeCN (Figure 3). This step is accompanied by an important loss of absorbance, indicating a significant change in the transition dipole moment, and is followed by a subtle absorption spectral change occurring over 10–15 ps. This latter process corresponds to solvent assisted relaxation of S_1_ but involves only a minor perturbation of the absorption spectrum. The time constant for decay of the relaxed S_1_ state (τ_S’_ = 4.7 ± 0.2 ns) is comparable to that determined by time-correlated single photon counting. These transient absorption signals compare well with those reported^35^ for structurally related derivatives adsorbed on alumina, where charge injection into the host is forbidden.

In THF solution, the absorbance signal ascribed to S_1_ decays to the prepulse baseline, indicating negligible population of the triplet manifold, but in MeCN, there is a residual signal centered at *ca*. 500 nm, which persists onto the μs time scale (Figure S46). Interrogation of the system with a 4 ns laser pulse generates a small positive absorbance in the region around 500 nm, superimposed over the ground-state bleach, that recovers with a lifetime of 45 ± 5 μs (Figure S47). This latter species has the hallmarks of the triplet state as reported by Papadopoulos et al.^21,22^ following triplet–triplet energy transfer using a palladium porphyrin as the energy donor.

It is well documented^36−38^ that many diverse perylene dyes aggregate in solution and, for **PMIDE-m**, nonlinear absorption spectral changes accompany an increase in solute concentration from 1 × 10^–7^ to 1 × 10^–4^ M (Figures S10 and S11). There is a pronounced solvent effect. Thus, in MeCN, the lowest-energy absorption envelope broadens with increasing solute concentration due to the evolution of a new transition centered at *ca*. 470 nm (Figure S10). This spectral broadening is significantly accentuated in THF (Figure S11), allowing derivation of the absorption spectral profile for the aggregate present at high solute concentration (Figure 2b). This profile is dominated by a broad, intense band centered at 470 nm with a much weaker transition centered at 544 nm. Such band splitting is reminiscent^37^ of excitonic coupling as seen for many different families of aggregated chromophores. In the case of **PMIDE-m**, the splitting energy amounts to 1470 cm^–1^.^38,39^ The higher-energy absorption transition, identified as the H-state according to conventional molecular excitonic theory,^38^ accounts for more than 90% of the integrated absorption intensity. In turn, the lower–energy transition can be assigned to the J-state.^39^

For cofacially stacked chromophores having parallel transition dipole moments the transition from the ground state to the H-state is fully allowed, while the transition to the corresponding J-state is forbidden.^38,39^ The transition dipole moment vector for **PMIDE-m** is aligned with the long molecular axis, thereby suggesting that the intermolecular dimer has an average conformation with an almost cofacial parallel alignment of the two chromophores. This geometry would explain both the strong blue-shifted absorption maximum and the weak red-shifted transition. However, it should be noted that the selection rules for zero-order exciton states are sensitive to the existence of vibronic coupling effects.^38^

In earlier work,^27^ the homogeneous nucleation-elongation model developed by Goldstein and Stryer^40^ was used to analyze the absorbance vs concentration profiles recorded for **PMIDE**-**m** in MeCN and THF at 20 °C. Here, we are more concerned with restricted concentration ranges where transient absorption spectroscopy can be applied. Analysis of the concentration dependent absorbance measurements at modest concentrations (1 × 10^–7^ to 5 × 10^–5^ M) allowed determination of the equilibrium constant (*K*_2_) for self-association. For **PMIDE**-**m** in MeCN, where the underlying spectral changes are modest, the experimental *K*_2_ value is 1.6 ± 0.4 × 10^3^ M^–1^ and, under the conditions used for spectroscopic studies, aggregation is kept to a minimum. The derived *K*_2_ value of 1.1 ± 0.2 × 10^4^ M^–1^ indicates a stronger tendency toward aggregation in THF. Here, a mixture of monomer and aggregate is to be expected at higher solute concentrations, although aggregation is likely restricted to dimerization under our conditions.

Fluorescence spectra recorded at higher (i.e., >5 μM) concentrations show pronounced effects of self-absorption, despite the moderate Stokes shift. No new emission bands appear in these spectra and, in particular, there are no indications for excimer emission under these conditions. This contrasts with reports that weak, far-red fluorescence has been observed^41^ for thick films of certain perylene dyes and for high concentrations of a perylene diimide derivative dispersed in plastic films.^42^ Furthermore, Yoo et al.^43^ described the red-shifted fluorescence observed with covalently linked perylene diimide bi- and trichromophores as being excimer-like. It is perhaps notable that Lin et al.^20^ did not observe excimer fluorescence from either crystalline samples or thin films of a sterically encumbered PMI derivative not too dissimilar to **PMIDE**-**m**. The solubilizing chains appended to the latter might prevent adoption of the ideal geometry for excimer formation, while emission from residual monomer would likely obscure any weak fluorescence from the aggregate.

**Figure 4:**
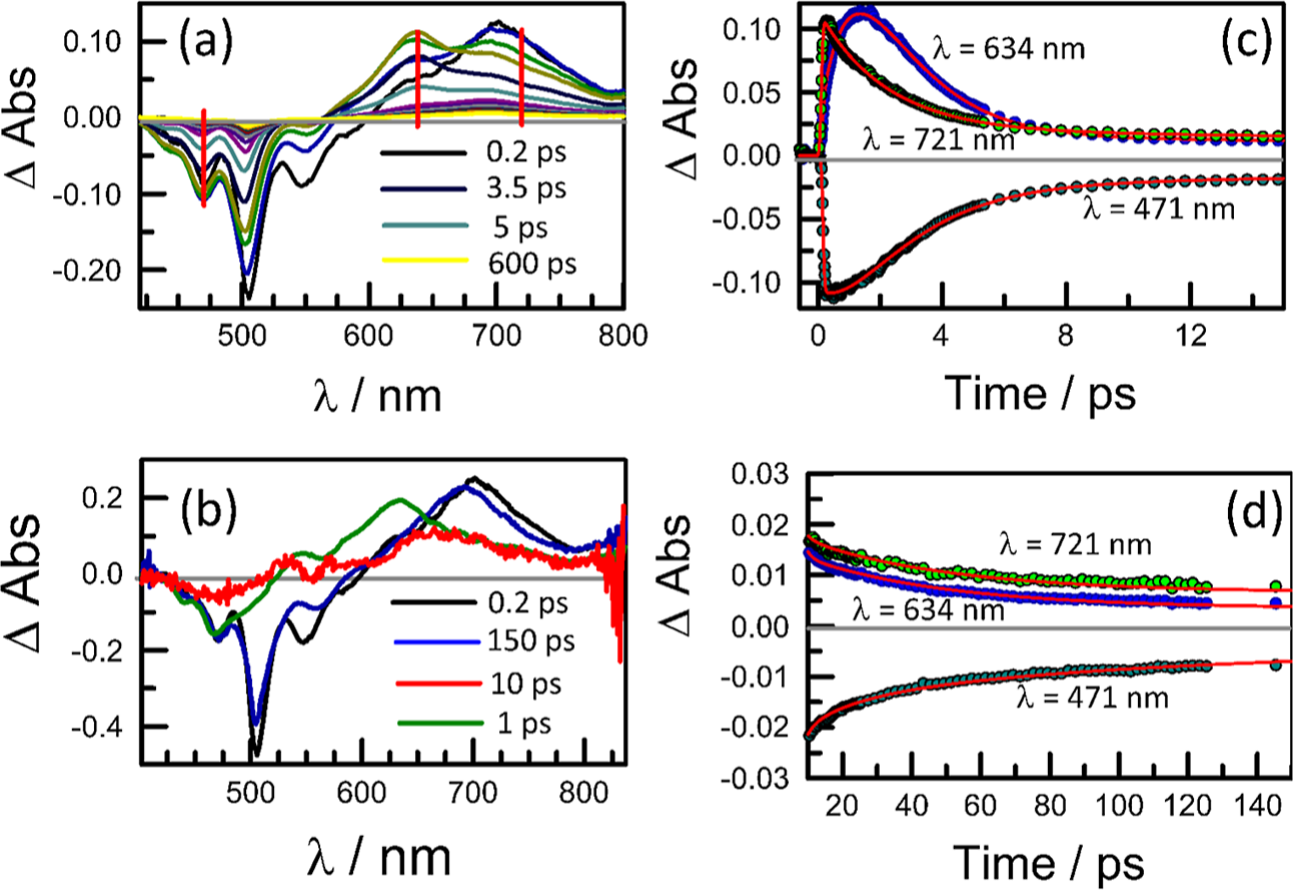
(a) Selection of transient differential absorption spectra recorded after excitation of **PMIDE**-**m** in THF (*C*_T_ = 100 μM; α_D_ = 0.62; *F*_D_ = 48%). The vertical lines indicate the wavelengths corresponding to the following kinetic plots. See Figure S32b for full color coding. Individual spectra were recorded at delay times of 0.2, 0.4, 0.8, 1.5, 3, 5, 10, 20, 40, 80, 150, 300, 600, and 1000 ps. (b) Differential absorption spectra derived for the principle transient species; S_FC_ (0.5 ps; black), S_1_ (5 ps; blue), the excitonic state (excited J-state) associated with the aggregate (1 ps; green), and the spin-correlated, triplet pair (10 ps; red). The latter curve is presented at 10× amplification. (c) Examples of kinetic profiles recorded following excitation of **PMIDE**-**m** in THF. The red curve corresponds to a nonlinear least-squares fit to the experimental data, with the lifetimes reported in the text. (d) Same examples as reported in (c) but on a longer time scale. See Figures S32–S34 for additional examples.

The transient absorption spectral landscape did not change noticeably with increasing concentration for **PMIDE**-**m** in MeCN. This was not the case with THF solutions, however, where self-association is more pronounced. For example, at a solute concentration where roughly 60% of **PMIDE**-**m** is present as a π-stacked aggregate, laser excitation at 500 nm gives rise to two independent sets of signals (Figure 4a). In the far-red region, absorption spectral changes remain as found for dilute THF and MeCN solutions. The initial S_FC_, which is formed in the excitation pulse, relaxes to S_1_ with a time constant of 2.8 ± 0.2 ps, the latter decaying to the ground state with a lifetime of 3.9 ± 0.6 ns (Figure 4d). These processes are conveniently monitored at wavelengths longer than 700 nm, although there is a minor contributor to the decay process with a lifetime of *ca*. 80 ps. There is an additional transient absorption signal, having a maximum at *ca*. 635 nm, which decays with a time constant of 3.7 ± 0.4 ps, that is not apparent in dilute solution. This decay step is much faster than deactivation of the thermally equilibrated S_1_ state, indicating its independence from the latter species.

Since the 635 nm transient is seen only at elevated concentrations, it must be associated with an aggregated species. Its differential absorption spectrum shows weak absorption in the far-red region and bleaching in the blue region but no stimulated fluorescence (Figure 4a,d). The spectrum bears a superficial similarity to that characterized for S_1_, but shifted toward higher energy. Interestingly, the derived absorption spectrum resembles that reported by Papadopolous et al.^22^ for a *meta*-phenyl bridged PMIDE-based bichromophore in MTHF at 140 K. These latter conditions should favor self-association and indeed it was observed that slow cooling of a solution of **PMIDE**-*m*-**d** in MTHF to 170 K caused splitting of the absorption spectrum (Figure S15). At this temperature, the solvent remains in the liquid phase but the absorption spectrum displays the characteristic features of the aggregate seen at high concentration. This observation lends further support to the notion that the 635 nm transient is related to an aggregate.

**Figure 5:**
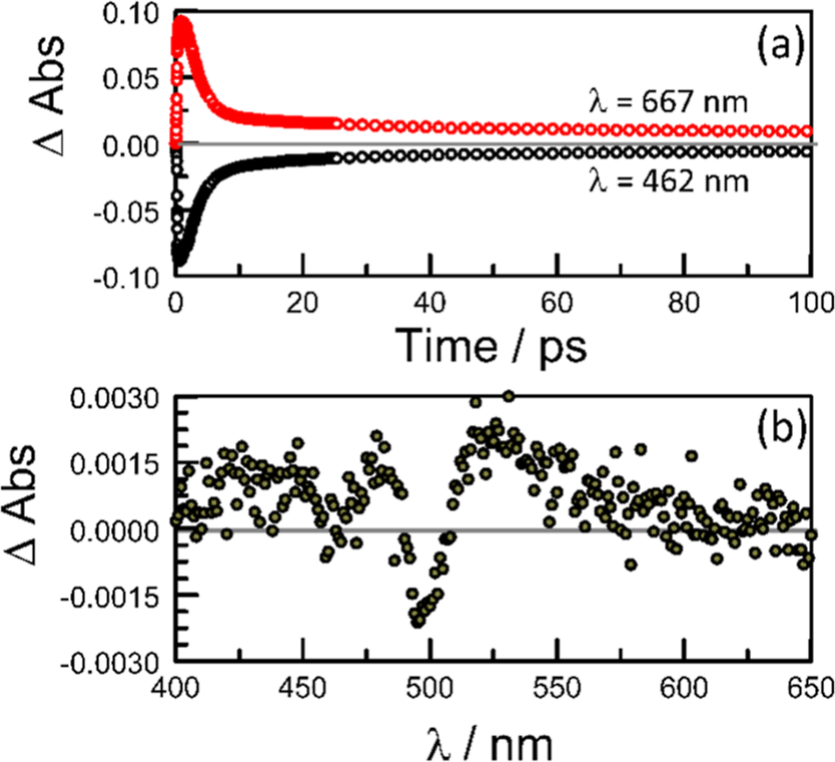
(a) Plots of ground-state recovery at 462 nm, where the aggregate is the principle absorber, and for the successive decay of the excited J-state and ^1^[T,T] at 667 nm. Excitation was at 500 nm with a 120 fs laser pulse. (b) Transient differential absorption spectrum recorded 50 ns after illumination of **PMIDE**-**m** in deoxygenated THF (*C*_T_ = 100 μM; α_D_ = 0.62; *F*_D_ = 48%) with a 4 ns laser pulse at 460 nm.

Excitation at 500 nm corresponds to absorption into the upper vibronic envelope of the J-state and the red-edge of the H-state (Figure 2b). Formation of the 635 nm transient occurs via two steps, both of which are very fast (Figure 4c). The first such step takes place within the excitation pulse but there is a slower change having a time constant of 1.8 ± 0.2 ps. The signal decays with a global lifetime of 4 ± 1 ps (Figure 4c). This latter decay, which is accompanied by partial recovery of ground-state absorption in the region of 470 nm (Figure 4a), forms an additional transient species that decays with a lifetime of 80 ± 10 ps (Figure 4a). This fast recovery of the ground-state bleach can be seen most clearly in the wavelength region around 470 nm, where the aggregate is the major absorber (Figure 5).

As such, we associate the 635 nm species with an excitonic state of the intermolecular dimer. Its rapid evolution can be attributed to direct excitation into the J-state, with a further contribution arising through internal conversion from the H-state. Giaimo et al.,^44^ while studying excimer-like emission from covalently linked PDI-based-dimers, observed fluorescence from the J-state. This emission was especially evident at higher temperatures, where fewer molecules exist in the geometry needed for excimer formation. Its lifetime, measured at 550 nm, was 50 ps. No accompanying emission could be detected in our case, although there is still residual fluorescence from the monomer that renders such detection hazardous. The rapid decay of the excited J-state, relative to that reported by Giaimo et al.^44^ , can be attributed to the stronger excitonic coupling within the dimer and/or an additional quenching step.

In competition with fast internal conversion, the excited J-state decays to form a transient species having weak absorption across the red region (Figure 4b). The absorption maximum lies at *ca*. 670 nm, with a broad shoulder stretching as far as 800 nm. The signal decays with a mean lifetime of 80 ± 10 ps. In the wavelength range around 470 nm, there is further recovery of the ground-state absorption, which occurs with a mean lifetime of 80 ± 15 ps (Figure 5a). This observation can be used to argue that this transient is also associated with the aggregate; certainly, it is not present in either MeCN or dilute THF solutions. On the crude assumption that neither of these aggregate-derived transients absorb appreciably at 470 nm, the kinetics for ground-state recovery indicate a yield for the longer-lived transient of *ca*. 13%.

The differential transient absorption spectrum derived for this 670 nm absorbing species bears a generic resemblance to that reported by Lin et al.^20^ for thin films of a sterically encumbered PMI and assigned to the spin-correlated, triplet pair. Also, Papadopolous et al.^21^ reported similar differential absorption spectra for related bichromophores at room temperature where ineffective triplet formation occurs. Consequently, we assign the 670 nm species to ^1^[T,T] localized on the aggregate. For **PMIDE**-**m** in THF, the energy of the J-state (*E*_J_ = 18,380 cm^–1^) is lower than that of the S_1_ state for the monomer (*E*_S_ = 19,230 cm^–1^) but slightly higher than that assigned to the spin-correlated, triplet pair (*E*_TT_ ≈ 17,800 cm^–1^). As such, SEF might be expected on energetic grounds.

The low yield of ^1^[T,T], estimated as being *ca*. 13% from the kinetic plots (Figure 5a), is a consequence of the short inherent lifetime of the precursor excited J-state. Excitation of the solution with a 4 ns laser pulse at 500 nm leads to positive but indistinct absorption in the blue region and a slight depletion of the ground-state absorption band at 500 nm (Figure 5b). Recovery of this transient bleaching occurs with a lifetime of *ca*. 90 ns (Figure S48). This might be indicative of formation of the isolated triplet, but if so, the yield is extremely low. As such, we conclude that ^1^[T,T] decays primarily by way of fusion to form a crop of the excited J-state. This situation arises because the spin-correlated triplet pair cannot diffuse apart.

**Scheme 1:**
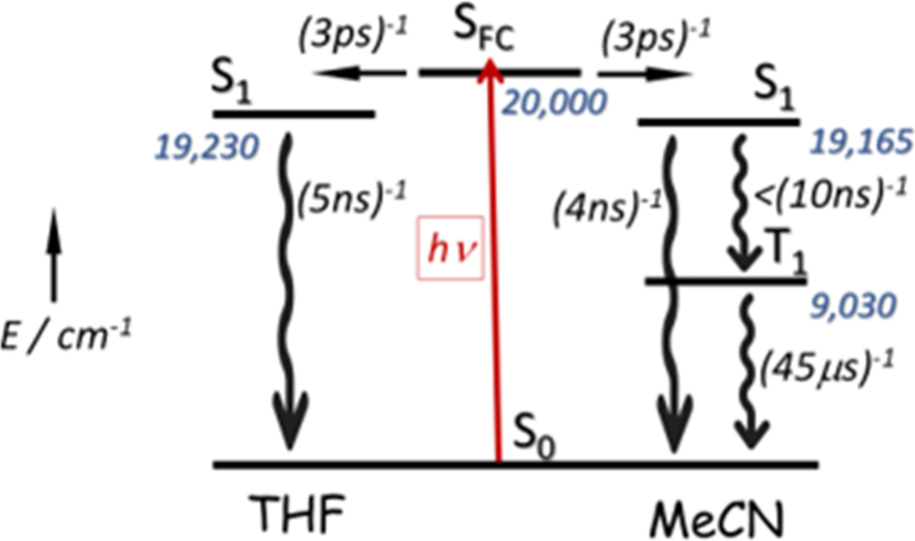
Outline of the Fundamental Processes that Follow Excitation of **PMIDE**-**m** in Dilute Solution, Emphasizing the Ineffective Triplet Formation Observed in MeCN Numbers given in blue refer to excitation energies in cm^–1^, while numbers given in black refer to rate constants for a particular process, as derived from the spectroscopic studies. The rate constant for intersystem crossing is estimated from the very low triplet yield and the S_1_ lifetime.

**Scheme 2:**
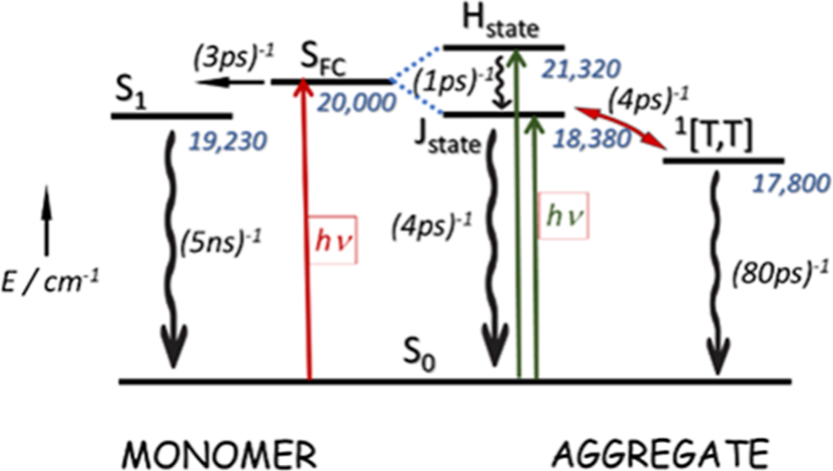
Reaction Sequence Proposed for Excitation of a System Having Monomer and Dimer in Equilibrium with both Species Absorbing Excitation Energy The two processes, associated with absorption by monomer and aggregate, are independent. Excitation of the monomer is believed to follow the same route as for dilute solution. Parallel excitation of the aggregate leads to formation of the excited J-state, either directly or *via* fast relaxation of the excited H-state. The spin-correlated, triplet pair, which is formed from the excited J-state, decays primarily via triplet–triplet fusion. Excitation energies are given in units of cm^–1^.

The photophysical properties of **PMIDE**-**m** are summarized in Schemes 1 and 2. In THF, the isolated monomer follows the two-state model indicated by Papadopoulos et al.,^21,22^ but there is an extra step in the more polar MeCN. Here, a small crop of the T_1_ state is observed on the microsecond time scale (Figures S46 and S47). The unfavorable *K*_2_ in MeCN means that it is difficult to convert a reasonable fraction of the monomer into an intermolecular dimer. Consequently, over our limited range, there is no real effect of concentration on the photophysics of **PMIDE**-**m** in MeCN. This is not the case in THF, however, where π-stacking occurs readily, and the aggregate absorbs strongly in the blue. Excitation of this aggregate leads to rapid (i.e., 4 ps) formation of ^1^[T,T], which cannot separate and instead undergoes fast deactivation by way of triplet–triplet annihilation. Thus, self-association promotes SEF but does not facilitate the evolution of a long-lived triplet species in the case of the dimer.

### Photophysical Properties of PMIDE-*m*-d

During initial preparation of this manuscript, a report by Papadopolous et al.^22^ described spectroscopic studies made with a *meta*-phenyl bridged bichromophore structurally similar to **PMIDE**-*m*-**d**. Our results, described briefly below, remain in good agreement with this earlier report, although there are a few disparities in the details. Here, we note that absorption and fluorescence spectra recorded for **PMIDE**-*m*-**d** closely resemble those described for **PMIDE**-**m** (Figure S6). In particular, there are no signs of excitonic interactions^37−39^ between the two PMIDE chromophores in THF or MeCN. Band maxima, and the underlying vibronic patterns, are as outlined above (Figure S7). The molar absorption coefficient (ε_MAX_) measured at the absorption peak is within experimental error of being twice that found for the monomer in a dilute THF solution. The Stokes shift, measured from reduced spectra after deconstruction into Gaussian components, is *ca*. 700 cm^–1^ (Figure S7), indicating a modest structural relaxation on excitation.^45^ The fluorescence quantum yield (ϕ_F_) is slightly lower than that determined for **PMIDE**-**m** (Table 2). Time-resolved fluorescence decay curves, after excitation at 460 nm for dilute solutions, conform to a lifetime of about 5 ns.

**Table 2:** Compilation of the Photophysical Properties Derived for the Two PMIDE-Based Bichromophores in Dilute THF Solution at Room Temperature; Values Found in MeCN Are Given in Parentheses

property	PMIDE-*m*-d	PMIDE-*p*-d
ν_ABS_/cm^–1^	19,940(20,080)^a^	19,905(20,060)^a^
ν_FLU_/cm^–1^	19,215(19,180)^b^	19,270(19,255)^b^
ε_MAX_^c^	98,250	96,880
Φ_F_	0.60 (0.58)	0.61 (0.59)
τ_S_/ns^d^	4.3 ± 0.1(4.5 ± 0.2)	4.6 ± 0.2(4.2 ± 0.1)
τ_R_/ps^e^	2.5 ± 0.5(3.5 ± 0.5)	3.0 ± 0.2(3.2 ± 0.3)
τ_EQ_/ps^f^	175 ± 12 (94 ± 6)	120 ± 15 (70 ± 6)
τ_D_/ns^g^	4.0 ± 0.5(4.5 ± 0.5)	4.5 ± 0.5(4.0 ± 0.5)

a 0,0 absorption transition.

b 0,0 fluorescence transition.

c Molar absorption coefficient at the band maximum in units of M^–1^ cm^–1^.

d Fluorescence lifetime determined by TC-SPC.

e Time constant for relaxation of the Franck–Condon state to the thermally equilibrated excited-singlet state.

f Time constant for establishing the equilibrium mixture.

g Lifetime for decay of the equilibrium mixture from transient absorption spectroscopy.

**Figure 6:**
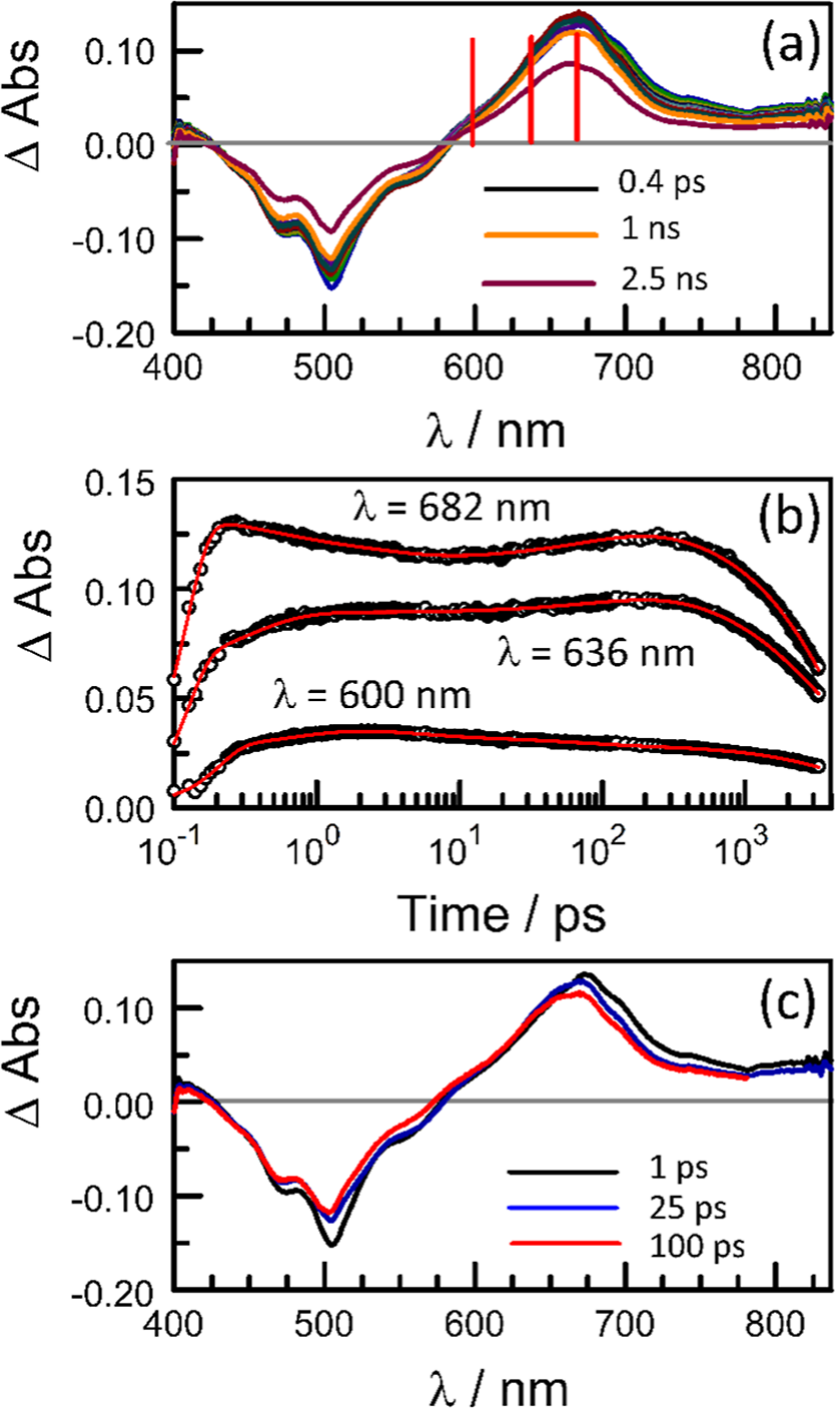
(a) Differential absorption spectra recorded after excitation at 500 nm of **PMIDE**-*m*-**d** in MeCN ((*C*_T_ = 40 μM; α_D_ = 0.08; *F*_D_ ≈ 3%). Individual spectra were recorded at delay times of 0.4, 0.6, 1, 2, 4, 8, 20, 50, 100, 200, 500, 1000, and 2500 ps. The vertical lines correspond to the wavelengths used for the kinetic studies. (b) Illustrative kinetic plots constructed at various wavelengths for the spectra indicated in (a). The experimental points are shown as open circles and the fits appear as red lines. The quoted lifetimes arise from global fitting over the full spectral range. (c) Derived differential absorption spectra assigned to S_FC_ (1 ps; black), S_1_ (25 ps; blue), and the equilibrium mixture (100 ps; red). See also Figures S35–S37, including full color coding.

Ultrafast pump–probe experiments made with **PMIDE**-*m*-**d** in a dilute solution showed the familiar transient differential absorption spectral features described above for the monomer. Immediately after excitation at 500 nm, the Franck–Condon state (S_FC_) can be recognized with absorption maxima at 680 and 710 nm, respectively, in MeCN and THF, and with a pronounced negative signal centered at around 510 nm (Figures 6 and S35–S37). This initial species undergoes fast solvent-assisted, structural relaxation over a few picoseconds; the wavelength-averaged time constant (τ_R_) is given in Table 2. Relaxation is accompanied by a blue shift of *ca*. 10 nm and a modest loss of absorbance across the far-red wavelength range. During this latter spectral evolution, there is a slight red shift and a decrease in intensity of the stimulated emission signal centered at 550 nm. This fast relaxation of S_FC_ should form the thermally equilibrated S_1_ state in quantitative yield in both solvents and is complete within *ca*. 10 ps. In turn, S_1_ decays over a few ns via first-order kinetics (Table 2). These spectral features are fully consistent with measurements made with related monomeric PMI derivatives prepared as thin films on alumina.^35^

The transient absorption spectra recorded for **PMIDE**-*m*-**d** exhibit small changes on the time scale of 100 ps or so (Figure S36). These changes are difficult to resolve in the far-red region but become clearer at shorter wavelengths (Figures 6 and S35–S37). SADS^46,47^ indicate the overall involvement of three species in both solvents. The far-red-absorbing species exhibit spectra similar to those for the monomer, such that identification is straightforward. In MeCN, the SADS derived for the third species remains similar to that assigned to S_1_ but with slight undulations around 580–670 nm and a reduced signal in the bleaching region (Figure 6c). This transient decays with the same lifetime as S_1_ and, therefore, is assigned to an equilibrium mixture comprising S_1_ and a third species. In order to establish an equilibrium, the participating states must have closely comparable excitation energies and this provides the rationale for identification.^48^ In THF solution, there is little difference between the SADS for S_1_ and the equilibrium mixture, suggesting a small contribution from the third species.

**Figure 7:**
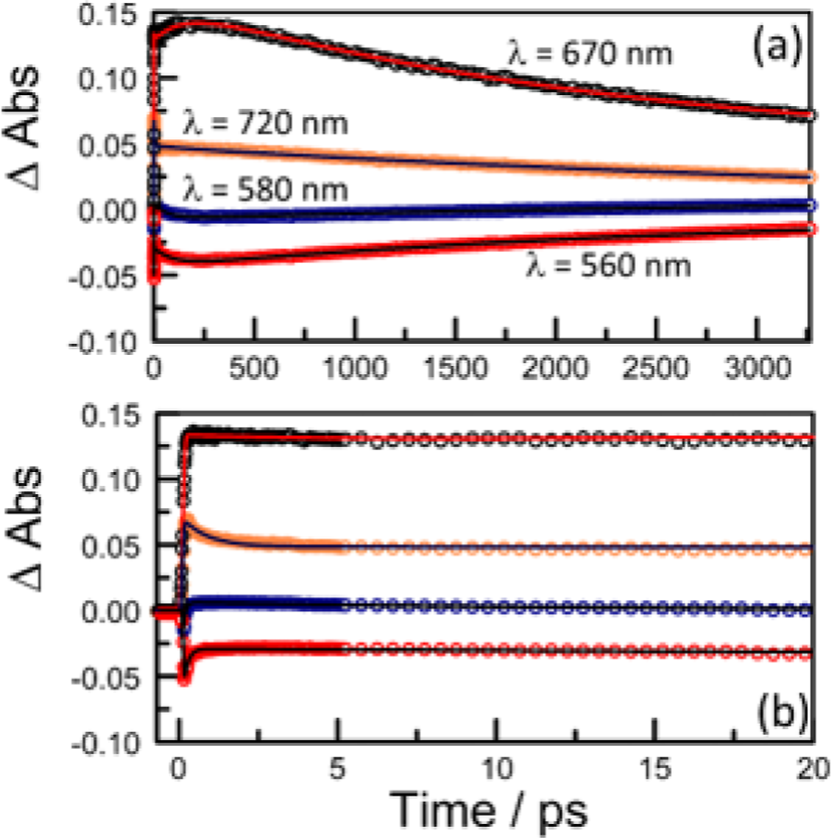
Further examples of kinetic plots recorded for **PMIDE**-*m*-**d** in MeCN (*C*_T_ = 40 μM; α_D_ = 0.08; *F*_D_ ≈ 3%) at room temperature covering (a) full temporal profile and (b) early times. In each case, the nonlinear least-squares fit is shown as a solid line with the experimental data shown as open circles. The monitoring wavelengths for (b) follow those for (a).

The equilibrium mixture decays with a mean lifetime (τ_D_) of 4.7 ± 0.5 ns in MeCN (Figure 7), which is comparable to that determined by time-correlated, single-photon counting at room temperature. The corresponding lifetime measured in THF is 4.0 ± 0.5 ns. There is no long-lived, isolated triplet state in either solvent, as evidenced by ns transient absorption spectroscopy. The averaged lifetime for the equilibration step (τ_EQ_) in MeCN, obtained from global analysis, is 94 ± 6 ps (Figure 7) while that in THF is slower (τ_EQ_ = 175 ± 12 ps). The excitation energy of S_1_, taken simply as the 0,0 transition for fluorescence, is 19,270 cm^–1^ (i.e., 2.38 eV); This will be a slight underestimate.

The prime candidate for the third intermediate is the spin-correlated, triplet pair, ^1^[T,T], which could reform S_1_ by way of triplet–triplet annihilation.^21,22^ However, the excitation energy for the triplet pair is *ca*. 18,000 cm^–1^ (i.e., 2.24 eV), which leaves a significant energy shortfall for reverse population of S_1_. Also, we would not have expected a significant solvent effect on the rates of equilibration had the equilibrium been between S_1_ and ^1^[T,T]. In order to be more definitive, the differential absorption spectrum for the third species was extracted from the corresponding SADS using a reiterative nonlinear, least-squares fit,^49^ employing the Levenberg–Marquardt algorithm.^50^ The underlying model assumes that the equilibrium is between S_1_ and this third species and that the spectrum for S_1_ is available from measurements made at shorter times. The resultant differential spectrum, obtained after considerable averaging of spectra recorded at different time delays, shows bleaching of the ground state and reasonably well-defined maxima at *ca*. 590 and *ca*. 660 nm. There is additional, but weaker, absorption stretching across the far-red region (Figure S53). The final spectrum does not resemble that assigned to ^1^[T,T] in the case of the **PMIDE**-**m** intermolecular dimer (cf. Figures 4b and S53).

A second candidate for the participating intermediate is a charge-transfer state (CTS) formed by light-induced electron transfer along the molecular axis. In fact, there have been several reports of light-induced symmetry breaking in somewhat related multichromophores built from PMI derivatives.^51,52^ Differential pulse voltammetry measurements made for **PMIDE**-**m** in MeCN indicate that the difference between the potential for the first reduction step (*E*_RED_ = −1.24 V vs Fc/Fc^+^) and the first oxidation step (*E*_OX_ = 1.23 V vs Fc/Fc^+^) is 2.47 eV (Figure S49); note that the oxidative step was not fully reversible in electrochemical terms. Allowing for electrostatic effects,^53^ the energy gap between the CTS and the ground state is estimated as being *ca*. 2.43 eV in MeCN and slightly higher in THF. Assuming the same reduction potentials apply to the bichromophores, this places the CTS at an energy slightly above that of the relaxed S_1_ state. Dependent on the reorganization energy,^53^ this small energy disparity should allow equilibration between the two states at ambient temperature. The slower rate of equilibration found for THF relative to MeCN also appears to be consistent with the third intermediate being an intramolecular CTS.

The differential absorption spectrum assigned to the third species (Figure S53) can be compared to the combination of spectra recorded for the π-radical anion and the corresponding π-radical cation (Figure S52). The spectrum for the former species was obtained by spectroelectrochemistry performed with **PMIDE**-**m** in deaerated MeCN (Figure S50). Reduction was made at −1.2 V vs Fc/Fc^+^ and kept to low conversion to avoid the formation of the π-dianion. The derived differential absorption spectrum has a pronounced peak at 648 nm, together with weaker transitions covering much of the far-red region (Figure S50). The spectrum is comparable to that reported by Pearce et al.^54^ for a related PMIDE derivative in CH_2_Cl_2_ and similar to that described by Kamire et al.^35^ for a PMIDE derivative after hole injection into NiO. On the assumption that the π-radical anion has negligible absorption at 500 nm, the molar absorption coefficient at the peak corresponds to 73,250 M^–1^ cm^–1^ in MeCN.

Although Pearce et al.^54^ successfully obtained the absorption spectrum for a PMIDE π-radical cation in CH_2_Cl_2_ using spectroelectrochemistry, we were less successful with oxidation of **PMIDE**-**m** in MeCN (or CH_2_Cl_2_) using this technique. Instead, one-electron oxidation was achieved by the photolysis of **PMIDE**-**m** in aerated CH_2_Cl_2_ containing CCl_4_ (20% v/v). Under these conditions, oxidation of the solute occurs through electron transfer to CCl_4_ and also through reaction of the trichloroperoxyl radical formed on ejection of a chloride ion from CCl_4_^–.^.^55^ The derived absorption spectrum (Figure S51) is similar to that reported by Pearce et al.^54^ and not unlike that observed by Lindquist et al.^56^ following charge injection into TiO_2_ from a PMIDE derivative. The absorption peak for the π-radical cation occurs at 581 nm, where the molar absorption coefficient is 56,500 M^–1^ cm^–1^, as calculated on the basis of negligible absorption at 500 nm.

Using the differential absorption spectra derived for the π-radical ions, the idealized spectrum for the CTS can be compiled by assuming zero interaction between the terminals (Figure S52); we would expect that the *meta* linkage would help minimize any such electronic interaction. In MeCN solution, agreement between the composed and measured spectra is deemed to be very good, despite the weak signal. The two maxima are clearly evident in the experimental spectrum, with peaks at 589 and 658 nm. The ratio of the peak intensities is more in favor of the π-radical cation than seen in the composed spectrum, but the disparity is not too serious (Figure S53). Unfortunately, it was not feasible to extract a reliable spectrum for the third component in THF solution, despite multiple averaging, because of excessive random noise (Figure S38). Finally, the low yield of the CTS precludes any comment on possible charge-recombination within the CTS forming a triplet state.^52^

**Scheme 3:**
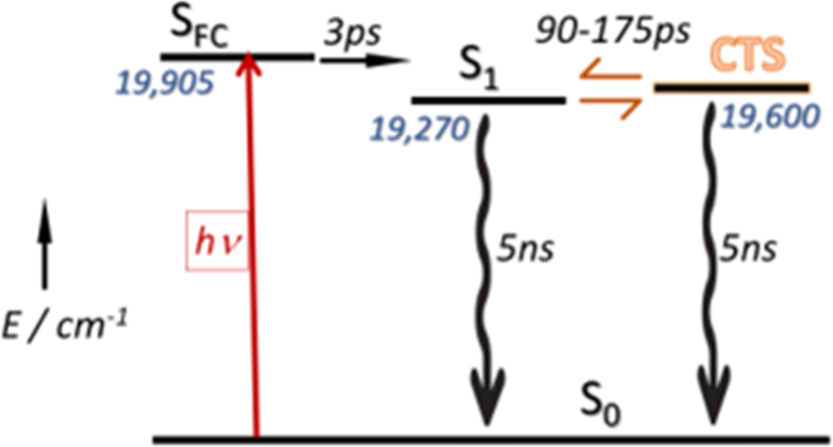
Energy Level Diagram Proposed for the Photo-Induced Processes Relevant to **PMIDE**-**m**-**d** and **PMIDE**-**p**-**d** Bichromophores in Dilute Solution The key step is equilibration between the relaxed S_1_ and an intramolecular charge-transfer state (CTS) lying at slightly higher energy. Excitation energies are for MeCN and are given in units of cm^–1^, while the approximate time constants are taken from the transient absorption spectral measurements.

Our understanding of the photophysics of the **PMIDE**-*m*-**d** bichromophore is sketched in Scheme 3. There is little or no electronic interaction between the two polycycles such that excitation forms a Franck–Condon (FC) state localized on one of the PMIDE units. The DFT calculations provide support for this situation in the form of localized transition dipole molecular orbitals (Figures S27 and S29). Fast relaxation of S_FC_ forms the thermally equilibrated S_1_ state within 5 ps or so, with the corresponding Stokes shift being *ca*. 700 cm^–1^. This step is accompanied by small alterations to the transient absorption envelope and a slight narrowing of the main absorption band centered around 700 nm (Figures 6 and S35–S37). Once formed, the S_1_ state acquires more charge-transfer character, leading to further minor changes in the absorption spectrum. This latter process is described in terms of an equilibrium with a CTS of comparable excitation energy. This step is unique to the bichromophore. There is a small solvent effect, although this was not studied in detail, and there should be a corresponding temperature dependence. The S_1_ state dominates the equilibrium mixture and gives rise to a fluorescence spectrum indistinct from that of the monomer.

Steady-state absorption spectral studies^37−39^ made with **PMIDE**-*m*-**d** in MeCN did not show signs of self-association at concentrations less than 80 μM (Figure S12). Likewise, the corresponding fluorescence spectra show self-absorption^57^ but only emission from the monomer (Figure S16). As found for **PMIDE**-**m**, the polar solvent did not facilitate self-association. Surprisingly, aggregation was also inhibited in THF, occurring with *K*_2_ = 6.7 × 10^2^ M^–1^. Presumably, the “bent” geometry, together with rotation around the connections, hinders the adoption of a π-stacked structure. Optical spectra recorded at high (i.e., 40 μM) concentrations show broadening but no obvious splitting of the main absorption transition in THF solution (Figure S12). Apart from self-absorption, the fluorescence spectrum is independent of concentration in THF at room temperature (Figures S17 and S18). The same situation was observed for ultrafast transient absorption spectroscopy where neither the band shape nor decay rate was much affected upon increasing solute concentration.

### Photophysical Properties of PMIDE-*p*-d

The *para*-derivative, **PMIDE**-*p*-**d**, exhibits the π–π* transitions and vibronic signatures expected from studies made with the other members of this series and also with related compounds (Figures S8 and S9). Absorption and emission maxima remain closely comparable to those of **PMIDE**-**m** in dilute solution (Table 2) while spectroscopic measurements gave no indication of excitonic coupling between the two PMIDE units within the bichromophore.^58^ As with the monomer, there is only superficial mirror symmetry between absorption and fluorescence spectra (Figure S8), while the Stokes shift remains similar to that of the monomer. The fluorescence quantum yield measured for the bichromophore is slightly lower than that observed for the monomer in a dilute solution (Table 2). Time-resolved fluorescence decay curves were well represented as monoexponential processes, with a lifetime of 4.2 ± 0.1 ns in MeCN at room temperature (Table 2). Overall, there are no obvious differences between the two bichromophores in terms of their general photophysical properties.

**Figure 8:**
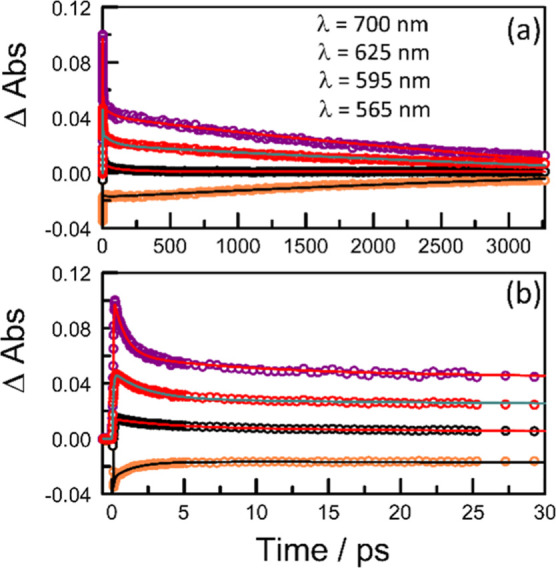
Examples of kinetic plots recorded for **PMIDE**-*p*-**d** in MeCN ((*C*_T_ = 50 μM; α_D_ = 0.15; *F*_D_ ≈ 4%) at room temperature covering (a) full temporal profile and (b) early times. In each case, the nonlinear least-squares fit is shown as a solid line with the experimental data shown as open circles. The monitoring wavelengths for (b) follow those for (a).

Laser excitation of **PMIDE**-*p*-**d** in dilute MeCN or THF solution gave transient absorption spectral changes similar to those noted above for **PMIDE**-*m*-**d** (Figures S39–S42). Thus, excitation forms the Franck–Condon state, with an absorption maximum at 705 and 730 nm, respectively, in MeCN and THF. This species transforms to the relaxed S_1_ state over a few ps, evidenced by a modest blue shift and small loss of absorbance, while the latter state decays on the ns time scale (Table 2). The emitting S_1_ state shows absorption maxima at 680 and 700 nm, respectively, in MeCN and THF. The accompanying SADS^46,47^ indicate that a third species is present in both solvents (Figures S41 and S42), and this situation was confirmed by kinetic measurements. Indeed, global analysis indicates three kinetic steps, with mean lifetimes of 3.2 ± 0.3 ps, 70 ± 6 ps, and 4.0 ± 0.2 ns in MeCN (Figure 8). The fast process is readily assigned to relaxation of the FC state, which is most evident in the far-red region, where S_FC_ is the principle absorber. The slowest process corresponds to the lifetime measured by fluorescence spectroscopy. The intermediate step involves a small spectral change across the red region (Figures S41 and S42), which is again assigned to equilibration with the intramolecular CTS (Scheme 3). Global analysis for measurements made with **PMIDE**-*p*-**d** in THF at room temperature indicates mean lifetimes of 3.0 ± 0.2 ps and 4.5 ± 0.5 ns, corresponding to relaxation of S_FC_ and fluorescence from S_1_. Monitoring in the vicinity of 565 nm returns a lifetime of 120 ± 15 ps for the equilibration step. Notably, excitation with a 4 ns laser pulse at 460 nm does not give rise to a persistent signal on the microsecond time scale.

Equilibration between S_1_ and the CTS is somewhat faster for the *para*-isomer in both solvents, presumably reflecting an increase in the degree of through-bond electronic coupling.^59^ This might be expected for an electron transfer reaction since resonance forms^60^ can be drawn for the *para*-phenyl bridge, but not for the corresponding *meta*-phenyl, to help stabilize the intermediary virtual states. The differential absorption spectra derived by spectral deconstruction for the CTS are similar for both bichromophores and are only weakly affected by a change in solvent (Figure S53).

Increasing concentration had little effect on the absorption spectrum for **PMIDE**-**p**-**d** in MeCN, but there were clear changes in THF, consistent with aggregation of the solute (Figure S19). In this case, spectral broadening is accompanied by the appearance of a new transition in the blue region and the development of a weak transition on the red edge of the monomer absorption band. Deconstructing the spectrum into Gaussian components^61^ (Figure S21) allowed estimation of the 0,0 transitions for the H- and J-states, respectively, as 21,485 (i.e., 465 nm) and 18,290 cm^–1^ (i.e., 547 nm). The excitonic coupling strength, therefore, corresponds to *ca*. 1600 cm^–1^, which is somewhat exaggerated relative to the monomer in THF. Absorption to the H-state dominates the spectrum for the aggregate which indicates a head-to-head geometry with near parallel alignment of the transition dipole moment vectors (Figure S22).^39^ Indeed, the idealized dimer structure computed by DFT has near perfect alignment of the two **PMIDE**-*p*-**d** molecules, but with a minor offset and a separation of 3.5 Å (Figure S30). The calculated absorption spectrum for the dimer in vacuo has significant absorption transitions located at 20,160 cm^–1^ (*f* = 1.28) and 17,580 cm^–1^ (*f* = 0.11). The dimer is essentially nonemissive (Figures S23–S26).

Formation of a head-to-head dimer places the two remaining PMIDE units in an ideal position to form the second head-to-head dimer. This high level of cooperativity will minimize the significance of the 1:1 structure in favor of the 2:2 species. Over the concentration range 1 × 10^–7^ to 2 × 10^–5^ M in MeCN, the absorption spectral changes follow a linear pattern but deviate at higher concentrations (Figure S20). This was not the case in THF solution (Figure S20) where nonlinear concentration effects are observed, even at a quite low concentration. Restricting attention to formation of a dimer, spectroscopic analysis allows estimation^37−39^ of *K*_2_ as being 8 ± 2 × 10^3^ M^–1^. Fluorescence spectra show the familiar hallmarks of self-absorption^57^ with increasing solute concentration but without evolution of a new emission profile (Figures S23–S26).

**Figure 9:**
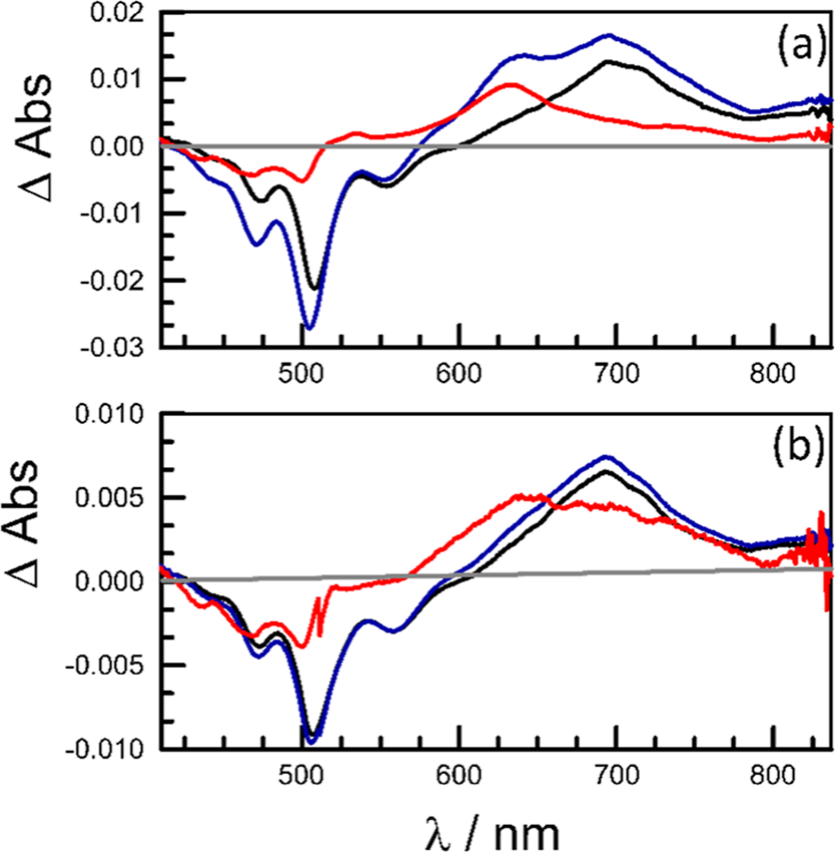
Examples of transient differential absorption spectra recorded after excitation of **PMIDE**-*p*-**d** in THF (*C*_T_ = 60 μM; α_D_ = 0.44; *F*_D_ ≈ 32%) at 490 nm. (a) Experimental spectrum at 1 ps (blue curve), known spectrum for S_FC_ (black curve) and derived spectrum for the excited J-state (red curve). (b) Experimental spectrum at 20 ps (blue curve), known spectrum for S_1_ (black curve) and derived spectrum for the third component (red curve); the latter is shown at 5× magnification.

Ultrafast pump–probe experiments in MeCN at solute concentrations in the range of 40–75 μM showed no significant differences, other than a variation in signal intensity. Diluting the sample with THF led to increased levels of excitation scattering without affecting the relaxation kinetics. At higher concentrations in THF, where we expect to find a mixture of free and aggregated bichromophore, there were clear differences in the transient absorption spectral profile on short time scales (Figure 9). For example, the differential absorption spectrum recorded at a delay time of 1 ps exhibits the features characterized for S_FC_, notably strong absorption in the far-red with a peak at 705 nm, but with an overlapping feature having a maximum at 640 nm (Figure 9a). Deconstruction of the overall spectrum into two components, one being fixed as S_FC_, allowed extraction of the second spectrum with a maximum at 635 nm. The derived spectrum (Figure 9a) resembles that attributed earlier to the excited J-state for the intermolecular aggregate (Figure 4b). In all likelihood, the same assignment holds here.

An equivalent procedure was applied to the spectrum attained with a delay time of 20 ps (Figure 9b). Here, S_1_ dominates the profile, having a maximum at 695 nm and features associated with ground-state depletion and stimulated emission across the range of 450–600 nm. Comparison of this spectrum with that assigned to S_1_, from studies made at lower concentration, shows a minor mismatch, notably across the far-red region (Figure 9b). This indicates the presence of an additional species that perturbs the spectral profile. Again, iterative reconstruction allowed us to establish that this species absorbs from 600 to 800 nm, but without a clear maximum (Figure 9b). The spectrum lacks a contribution from stimulated emission, while the ground-state depletion does not match the absorption profile of the monomer. The spectrum is not too dissimilar from that attributed earlier to ^1^[T,T], and we can conclude that the scenario for the aggregated forms of **PMIDE-m** and **PMIDE**-*p*-**d** share a common pathway.

The only reasonable alternative explanation for the reaction kinetics seen at high concentrations assigns the additional steps to structural relaxation processes for the intermolecular dimer. Indeed, Lewis et al.^62^ reported relevant observations made with an intermolecular dimer formed by association of two perylene diimide functionalized polynucleotide hairpins. In the presence of salt, laser excitation of the dimer caused fast (i.e., *ca*. 40 ps) evolution of a transient species absorbing around 650 nm. This latter species decayed with a lifetime of 1–2 ns and was assigned to an excimer formed from close association of the two aryl residues.^60^ The fast component, characterized by absorption band narrowing, could be attributed to structural changes as the excimer evolved from the ground-state species. The putative intermediary role of an excimer in the formation of the spin-correlated triplet pair remains an intriguing but unresolved issue.

### Concluding Remarks

Knowledge of the respective energy levels for the relaxed excited-singlet state (*E*_S_ ≈ 2.4 eV) and for the isolated triplet state (*E*_T_ ≈ 1.1 eV) for PMI diesters (PMIDEs) has fueled speculation that appropriate bichromophores might be effective agents for SEF.^21,22,53^ Calculations made at the DFT level indicate that the triplet state is localized on a single chromophore, thereby enhancing the SEF motivation. The two phenyl-linked PMIDE bichromophores studied here, however, do not exhibit any spectroscopic features^38,39^ suggestive of intramolecular excitonic coupling between the terminal polycycles. Excitation in solution forms a Franck–Condon state that transforms rapidly to the relaxed S_1_ state, discarding *ca*. 700 cm^–1^ of vibrational energy in the process. After relaxation, the S_1_ state enters into equilibration with a CTS, although the contribution of the latter is minimal. The equilibrium decays over a few nanoseconds in fluid solution at room temperature, without meaningful population of the triplet manifold (Scheme 3).

The situation is changed dramatically for face-to-face dimers formed by self-association of the PMIDE monomer in a weakly polar solvent. Here, the transition dipole moment vectors are almost perfectly aligned^56^ and the two chromophores are strongly coupled (Figure 2b). A consequence of this coupling is that excitonic splitting of the main absorption transition lowers the energy of the J-state and thereby reduces access to the CTS. In turn, this opens the door for population of the spin-correlated, triplet pair, as happens in crystals and thin films.^20^ In fact, the **PMIDE-m** aggregate can be considered as the smallest domain likely to persist in thin films, and the rates of formation and decorrelation of the triplet pair are not too dissimilar in these two systems. The complexity of the aggregate state hinders attempts to measure the yield of triplet formation, which is estimated by an indirect method to be *ca*. 13% of the precursor excited J-state. Also, a large domain is needed for decorrelation of the triplet pair to form isolated triplets, but this route is not available to the smallest aggregates.

Notably, SEF has been reported for a close derivative of **PMIDE**-**m**-**d**,^22^ although the yield was very low, and for the corresponding 2,7-naphthyl bridged bichromophore.^21^ In the latter case, where the triplet yield is also low at room temperature, efficient triplet–triplet annihilation reduced the lifetime of the spin-correlated triplet pair. Papadopolous et al.^21,22^ have stressed the importance of rotational freedom in determining the triplet yield in these bichromophores, and it appears that small structural modifications have an effect on the subtle balance affecting partitioning of S_1_ toward the CTS or ^1^[T,T] or indeed on interconversion between these two states. On our part, we have only seen ^1^[T,T], where there is strong electronic coupling between the chromophores. Fortunately, PMIDEs undergo facile π-stacking in certain solvents,^37^ such as THF, where excitonic interactions are pronounced. This seems to be the most viable route to the future development of systems displaying triplet yields.

## Supporting Information Available

Includes details for the synthesis and characterization of new compounds, spectroscopic data for the effects of increased concentration, examples of steady-state optical spectra and data fitting, details and additional materials supporting the computational studies, and further examples of spectra and reaction kinetics obtained through ultrafast transient absorption spectroscopy (PDF)

## Abbreviations

DFT: density functional theory
MeCN: acetonitrile
THF: tetrahydrofuran

## References

[ref1] Moyez A.; Dhar A.; Sarkar P.; Jung H. S.; Roy S. A Review of the Multiple Exciton Generation in Photovoltaics. Rev. Adv. Sci. Eng. 2016, 5, 51–64. 10.1166/rase.2016.1108.

[ref2] Miyata K.; Conrad-Burton F. S.; Geyer F. L.; Zhu X.-Y. Triplet Pair States in Singlet Fission. Chem. Rev. 2019, 119, 4261–4292. 10.1021/acs.chemrev.8b00572. 30721032

[ref3] Casillas R.; Papadopoulos I.; Ullrich T.; Thiel D.; Kunzmann A.; Guldi D. M. Molecular Insights and Concepts to Engineer Singlet Fission Energy Conversion Devices. Energy Environ. Sci. 2020, 13, 2741–2804. 10.1039/D0EE00495B.

[ref4] Rao A.; Friend R. H. Harnessing Singlet Exciton Fission to Break the Shockley–Queisser Limit. Nat. Rev. Mater. 2017, 2, 17063 10.1038/natrevmats.2017.63.

[ref5] Gray V.; Weiss L.; Rao A. Singlet Fission: Mechanisms and Molecular Design. In Emerging Strategies to Reduce Transmission and Thermalization Losses in Solar Cells; Lissau J. S., Madsen M., Eds.; Springer: Cham, 2022. 10.1007/978-3-030-7035

[ref6] de Sousa L. E.; dos Santos Born L.; de Oliveira Neto P. H.; de Silva P. Triplet-to-Singlet Exciton Transfer in Hyperfluorescent OLED Materials. J. Mater. Chem. C 2022, 10, 4914–4922. 10.1039/d1tc05596h.

[ref7] Smith M. B.; Michl J. Singlet Fission. Chem. Rev. 2010, 110, 6891–6936. 10.1021/cr1002613. 21053979

[ref8] Smith M. B.; Michl J. Recent Advances in Singlet Fission. Annu. Rev. Phys. Chem. 2013, 64, 361–386. 10.1146/annurev-physchem-040412-110130. 23298243

[ref9] Dover C. B.; Gallaher J. K.; Frazer L.; Tapping P. C.; Petty A. J. II; Crossley M. J.; Anthony J. E.; Kee T. W.; Schmidt T. W. Endothermic Singlet Fission is Hindered by Excimer Formation. Nat. Chem. 2018, 10, 305–310. 10.1038/nchem.2926. 29461531

[ref10] Finton D. M.; Wolf E. A.; Zoutenbier V. S.; Ward K. A.; Biaggio I. Routes to Singlet Exciton Fission in Rubrene Crystals and Amorphous Films. AIP Adv. 2019, 9, 095027 10.1063/1.5118942.

[ref11] Seiler H.; Krynski M.; Zahn D.; Hammer S.; Windsor Y. W.; Vasileiadis T.; Pflaum J.; Ernstorfer R.; Rossi M.; Schwoerer H. Nuclear Dynamics of Singlet Exciton Fission in Pentacene Single Crystals. Sci. Adv. 2021, 7, eabg0869 10.1126/sciadv.abg0869. 34172443 PMC8232917

[ref12] Broch K.; Dieterle J.; Branchi F.; Hestand N. J.; Olivier Y.; Tamura H.; Cruz C.; Nichols V. M.; Hinderhofer A.; Beljonne D.; Spano F. C.; Cerullo G.; Bardeen C. J.; Schreiber F. Robust Singlet Fission in Pentacene Thin Films with Tuned Charge Transfer Interactions. Nat. Commun. 2018, 9, 954 10.1038/s41467-018-03300-1. 29507287 PMC5838205

[ref13] Eaton S. W.; Shoer L. E.; Karlen S. D.; Dyar S. M.; Margulies E. A.; Veldkamp B. S.; Ramanan C.; Hartzler D. A.; Savikhin S.; Marks T. J.; Wasielewski M. R. Singlet Exciton Fission in Polycrystalline Thin Films of a Slip-Stacked Perylenediimide. J. Am. Chem. Soc. 2013, 135, 14701–14712. 10.1021/ja4053174. 24011336

[ref14] Aulin Y. V.; Felter K. M.; Günbas D. D.; Dubey R. K.; Jager W. F.; Grozema F. C. Morphology-Independent Efficient Singlet Exciton Fission in Perylene Diimide Thin Films. ChemPlusChem 2018, 83, 230–238. 10.1002/cplu.201700449. 31957287

[ref15] Ni W.; Sun L.; Gurzadyan G. G. Ultrafast Spectroscopy Reveals Singlet Fission, Ionization and Excimer Formation in Perylene Film. Sci. Rep. 2021, 11, 5220 10.1038/s41598-021-83791-z. 33664304 PMC7933242

[ref16] Ito S.; Nagami T.; Nakano M. Molecular Design for Efficient Singlet Fission. J. Photochem. Photobiol., C 2018, 34, 85–120. 10.1016/j.jphotochemrev.2018.01.002.

[ref17] Walker B.; Musser A.; Beljonne D.; Friend R. H. Singlet Exciton Fission in Solution. Nat. Chem. 2013, 5, 1019–1024. 10.1038/nchem.1801. 24256865

[ref18] Hecht M.; Würthner F. Supramolecularly Engineered J-Aggregates Based on Perylene Bisimide Dyes. Acc. Chem. Res. 2021, 54, 642–653. 10.1021/acs.accounts.0c00590. 33289387

[ref19] Greene M. in High Performance Pigments; Faulkner E. B.; Schwartz R. J., Eds.; Wiley VCH: Weinheim, 2009; p 261

[ref20] Lin C.-J.; Qi Y.; Brown P. J.; Williams M. L.; Palmer J. R.; Myong M.; Zhao X.; Young R. M.; Wasielewski M. R. Singlet Fission in Perylene Monoimide Single Crystals and Polycrystalline Films. J. Phys. Chem. Lett. 2023, 14, 2573–2579. 10.1021/acs.jpclett.2c03621. 36880847

[ref21] Papadopoulos I.; Gutiérrez-Moreno D.; McCosker P. M.; Casillas R.; Keller P. A.; Sastre-Santos A.; Clark T.; Fernández-Lázaro F.; Guldi D. M. Perylene-Monoimides: Singlet Fission Down-Conversion Competes with Up-Conversion by Geminate Triplet–Triplet Recombination. J. Phys. Chem. A 2020, 124, 5727–5736. 10.1021/acs.jpca.0c04091. 32567862

[ref22] Papadopoulos I.; Gutiérrez-Moreno D.; Bo Y.; Casillas R.; Greißel P. M.; Clark T.; Fernández-Lázaro F.; Guldi D. M. Altering Singlet Fission Pathways in Perylene-Dimers; Perylene-Diimide versus Perylene-Monoimide. Nanoscale 2022, 14, 5194–5203. 10.1039/D1NR08523A. 35315470

[ref23] Armin A.; Li W.; Sandberg O. J.; Xiao Z.; Ding L.; Nelson J.; Neher D.; Vandewal K.; Shoaee S.; Wang T.; Ade H.; Heumüller T.; Brabec C.; Meredith P. A History and Perspective of Non-Fullerene Electron Acceptors for Organic Solar Cells. Adv. Energy Mater. 2021, 11, 2003570 10.1002/aenm.202003570.

[ref24] Schweda B.; Reinfelds M.; Hofinger J.; Bäumel G.; Rath T.; Kaschnitz P.; Fischer R. C.; Flock M.; Amenitsch H.; Scharber M. C.; Trimmel G. Phenylene-Bridged Perylene Monoimides as Acceptors for Organic Solar Cells: A Study on the Structure–Property Relationship. Chem.—Eur. J. 2022, 28, e202200276 10.1002/chem.202200276. 35218252 PMC9313791

[ref25] Cremer J.; Bäuerle P. Perylene–Oligothiophene–Perylene Triads for Photovoltaic Applications. Eur. J. Org. Chem. 2005, 2005, 3715–3723. 10.1002/ejoc.200500147. 15750640

[ref26] Wang W.; Han J. J.; Wang L.-Q.; Li L.-S.; Shaw W. J.; Li A. D. Q. Dynamic π-π Stacked Molecular Assemblies Emit from Green to Red Colors. Nano Lett. 2003, 3, 455–458. 10.1021/nl025976j.

[ref27] Gültekin D. D. Aromatic Stacking and the Self-Assembly of Perylene Monoimide Diester Homodimers. J. Photochem. Photobiol., A 2022, 426, 113769 10.1016/j.jphotochem.2022.113769.

[ref28] Stephens P. J.; Devlin F. J.; Chabalowski C. F.; Frisch M. J. Ab Initio Calculation of Vibrational Absorption and Circular Dichroism Spectra Using Density Functional Force Fields. J. Chem. Phys. 1994, 98, 11623–11627. 10.1021/j100096a001.

[ref29] Tirado-Rives J.; Jorgensen W. L. Performance of B3LYP Density Functional Methods for a Large Set of Organic Molecules. J. Chem. Theory Comput. 2008, 4, 297–306. 10.1021/ct700248k. 26620661

[ref30] Yanai T.; Tew D. P.; Handy N. C. A New Hybrid Exchange–Correlation Functional using the Coulomb-Attenuating Method (CAM-B3LYP). Chem. Phys. Lett. 2004, 393, 51–57. 10.1016/j.cplett.2004.06.011.

[ref31] Syamala P. P.; Soberats B.; Görl D.; Gekle S.; Würthner F. Thermodynamic Insights into the Entropically Driven Self-Assembly of Amphiphilic Dyes in Water. Chem. Sci. 2019, 10, 9358–9366. 10.1039/C9SC03103K. 32110300 PMC7017873

[ref32] Ultrafast Systems, 8330; Consumer Ct, Sarasota, FL 34240, USA, 2024, Version 4.5.14 – January.

[ref33] Yarnell J. E.; Chakraborty A.; Myahkostupov M.; Wright K. M.; Castellano F. N. Long-lived Triplet Excited State in a Platinum(II) Perylene Monoimide Complex. Dalton Trans. 2018, 47, 15071–15081. 10.1039/C8DT02496K. 30303214

[ref34] Roy R.; Khan A.; Chatterjee O.; Bhunia S.; Koner A. L. Perylene Monoimide as a Versatile Fluoroprobe: The Past, Present, and Future. Org. Mater. 2021, 3, 417–454. 10.1055/a-1551-6930.

[ref35] Kamire R. J.; Majewski M. B.; Hoffeditz W. L.; Phelan B. T.; Farha O. K.; Hupp J. T.; Wasielewski M. R. Photodriven Hydrogen Evolution by Molecular Catalysts using Al_2_O_3_-Protected Perylene-3,4-dicarboximide on NiO Electrodes. Chem. Sci. 2017, 8, 541–549. 10.1039/C6SC02477G. 28616134 PMC5458681

[ref36] Lindquist R. J.; Lefler K. M.; Brown K. E.; Dyar S. M.; Margulies E. A.; Young R. M.; Wasielewski M. R. Energy Flow Dynamics within Cofacial and Slip-Stacked Perylene-3,4-dicarboximide Dimer Models of π-Aggregates. J. Am. Chem. Soc. 2014, 136, 14912–14923. 10.1021/ja507653p. 25245598

[ref37] Mativetsky J. M.; Kastler M.; Savage R. C.; Gentilini D.; Palma M.; Pisula W.; Müllen K.; Samorì P. Self-Assembly of a Donor-Acceptor Dyad Across Multiple Length Scales: Functional Architectures for Organic Electronics. Adv. Funct. Mater. 2009, 19, 2486–2494. 10.1002/adfm.200900366.

[ref38] Clark A. E.; Qin C.-Y.; Li A. D. Q. Beyond Exciton Theory: A Time-Dependent DFT and Franck-Condon Study of Perylene Diimide and Its Chromophoric Dimer. J. Am. Chem. Soc. 2007, 129, 7586–7595. 10.1021/ja0687724. 17518466

[ref39] Hestand N. J.; Spano F. C. Expanded Theory of H- and J-molecular Aggregates: The Effects of Vibronic Coupling and Intermolecular Charge Transfer. Chem. Rev. 2018, 118, 7069–7163. 10.1021/acs.chemrev.7b00581. 29664617

[ref40] Goldstein R. F.; Stryer L. Cooperative Polymerization Reactions: Analytical Approximations, Numerical Examples, and Experimental Strategy. Biophys. J. 1986, 50, 583–599. 10.1016/S0006-3495(86)83498-1. 3779001 PMC1329836

[ref41] Zhang F.; Ma Y.-S.; Chi Y.-H.; Yu H.; Li Y.; Jiang T.; Wei X.; Shi J. Self-assembly, Optical and Electrical Properties of Perylene Diimide Dyes Bearing Unsymmetrical Substituents at Bay Position. Sci. Rep. 2018, 8, 8208 10.1038/s41598-018-26502-5. 29844454 PMC5974014

[ref42] Haines C.; Chen M.; Ghiggino K. P. The Effect of Perylenediimide Aggregation on the Light Collection Efficiency of Luminescent Concentrators. Sol. Energy Mater. Sol. Cells 2012, 105, 287–292. 10.1016/j.solmat.2012.06.030.

[ref43] Yoo H.; Yang J.; Yousef A.; Wasielewski M. R.; Kim D. Excimer Formation Dynamics of Intramolecular pi-Stacked Perylenediimides Probed by Single-Molecule Fluorescence Spectroscopy. J. Am. Chem. Soc. 2010, 132, 3939–3944. 10.1021/ja910724x. 20184367

[ref44] Giaimo J. M.; Lockard J. V.; Sinks L. E.; Scott A. M.; Wilson T. M.; Wasielewski M. R. Excited Singlet States of Covalently Bound, Cofacial Dimers and Trimers of Perylene-3,4:9,10-bis(dicarboximide)s. J. Phys. Chem. A 2008, 112, 2322–2330. 10.1021/jp710847q. 18298107

[ref45] Petrenko T.; Neese F. Analysis and Prediction of Absorption Band Shapes, Fluorescence Band Shapes, Resonance Raman Intensities, and Excitation Profiles Using the Time-Dependent Theory of Electronic Spectroscopy. J. Chem. Phys. 2007, 127, 164319 10.1063/1.2770706. 17979350

[ref46] Roland T.; Heyer E.; Liu L.; Ruff A.; Ludwigs S.; Ziessel R.; Haacke S. A Detailed Analysis of Multiple Photoreactions in a Light-Harvesting Molecular Triad with Overlapping Spectra by Utrafast Spectroscopy. J. Phys. Chem. C 2014, 118, 24290–24301. 10.1021/jp507474r.

[ref47] Labrador T.; Dukovic G. Simultaneous Determination of Spectral Signatures and Decay Kinetics of Excited State Species in Semiconductor Nanocrystals Probed by Transient Absorption Spectroscopy. J. Phys. Chem. C 2020, 124, 8439–8447. 10.1021/acs.jpcc.0c01701.

[ref48] Harriman A.; Heitz V.; Ebersole M.; Van Willigen H. Intramolecular Electron and Energy Transfer within a Bisporphyrin in a Low Temperature Glass. J. Phys. Chem. 1994, 98, 4982–4989. 10.1021/j100070a007.

[ref49] Karnaukhov A. V.; Karnaukhova E. V.; Williamson J. R. Numerical Matrices Method for Nonlinear System Identification and Description of Dynamics of Biochemical Reaction Networks. Biophys. J. 2007, 92, 3459–3473. 10.1529/biophysj.106.093344. 17350997 PMC1853128

[ref50] More J. J. The Levenberg-Marquardt Algorithm: Implementation and Theory. In Numerical Analysis; Watson G. A., Ed.; Lecture Notes in Mathematics; Springer: Berlin, 1978; Vol. 630, pp 105–116. 10.1007/BFb0067700

[ref51] Roy R.; Chawla S.; Sharma V.; Pal A. K.; Silori Y.; Datta A.; De A. K.; Koner A. L. Ultrafast Symmetry-breaking Charge Separation in Perylenemonoimide-embedded Multi-chromophores: Impact of Regioisomerism. Chem. Sci. 2024, 15, 6363–6377. 10.1039/D3SC05325C. 38699268 PMC11062123

[ref52] Mazumder A.; Vinod K.; Maret P. D.; Das P. P.; Hariharan M. Symmetry-Breaking Charge Separation Mediated Triplet Population in a Perylenediimide Trimer at the Single-Molecule Level. J. Phys. Chem. Lett. 2024, 15, 5896–5904. 10.1021/acs.jpclett.4c01201. 38805687

[ref53] Wasielewski M. R. Photoinduced Electron Transfer in Supramolecular Systems for Artificial Photosynthesis. Chem. Rev. 1992, 92, 435–461. 10.1021/cr00011a005.

[ref54] Pearce N.; Davies E. S.; Champness N. R. Electrochemical and Spectroelectrochemical Investigations of Perylene *peri*-Tetracarbonyl Species. Dyes Pigm. 2020, 183, 108735 10.1016/j.dyepig.2020.108735.

[ref55] Alfassi Z. B.; Harriman A.; Mosseri S.; Neta P. Rates and Mechanisms of Oxidation of ZnTPP by CCl_3_O_2_ Radicals in Various Solvents. Int. J. Chem. Kinet. 1986, 18, 1315–1321. 10.1002/kin.550181203.

[ref56] Lindquist R. J.; Phelan B. T.; Reynal A.; Margulies E. A.; Shoer L. E.; Durrant J. R.; Wasielewski M. R. Strongly Oxidizing Perylene-3,4-dicarboximides for Use in Water Oxidation Photoelectrochemical Cells. J. Mater. Chem. A 2016, 4, 2880–2893. 10.1039/C5TA05790F.

[ref57] Clementi C.; Miliani C.; Verri G.; Sotiropoulou S.; Romani A.; Brunetti B. G.; Sgamellotti A. Application of the Kubelka-Munk Correction for Self-Absorption of Fluorescence Emission in Carmine Lake Paint Layers. Appl. Spectrosc. 2009, 63, 1323–1330. 10.1366/000370209790109058. 20030975

[ref58] Christ T.; Petzke F.; Bordat P.; Herrmann A.; Reuther E.; Müllen K.; Basché T. Investigation of Molecular Dimers by Ensemble and Single Molecule Spectroscopy. J. Lumin. 2002, 98, 23–33. 10.1016/S0022-2313(02)00247-8.

[ref59] Lin H.-C.; Jin B.-Y. Charge-Transfer Interactions in Organic Functional Materials. Materials 2010, 3, 4214–4251. 10.3390/ma3084214. 28883326 PMC5445831

[ref60] Karafiloglou P.; Launay J.-P. Local Description of the Through Phenyl Transfer of a Negative Charge within Resonance Theory: Topological Effects in Xylylene Radical Anions. Chem. Phys. 1999, 250, 1–12. 10.1016/S0301-0104(99)00278-5.

[ref61] Woodford O. J.; Ziessel R.; Harriman A.; Wills C.; Alsimaree A. A.; Knight J. G. Optical Spectroscopic Properties Recorded for Simple BOPHY Dyes in Condensed Media: The Mirror-Symmetry Factor. Spectrochim. Acta, Part A 2019, 208, 57–64. 10.1016/j.saa.2018.09.047. 30292151

[ref62] Hariharan M.; Zheng Y.; Long H.; Zeidan T. A.; Schatz G. C.; Vura-Weis J.; Wasielewski M. R.; Zuo X.-B.; Tiede D. M.; Lewis F. D. Hydrophobic Dimerization and Thermal Dissociation of Perylenediimide-Linked DNA Hairpins. J. Am. Chem. Soc. 2009, 131, 5920–5929. 10.1021/ja900347t. 19382814

