# Supplementary material for: Mono- and Bichromophores Formed from Perylene Monoimide Diesters: Competition between Intramolecular Charge Transfer and Intermolecular Singlet Exciton Fission
Source: J Phys Chem A. 2024 Oct 23;128(44):9614–26. doi: 10.1021/acs.jpca.4c05424 (PMC11551949; doi:10.1021/acs.jpca.4c05424)
Supplement: Supplementary file 1 — jp4c05424_si_001.pdf [file jp4c05424_si_001.pdf]

Mono- and Bichromophores Formed from Perylene Monoimide Diesters: Competition between Intramolecular Charge Transfer and Intermolecular Singlet Exciton Fission

Demet Demirci Gültekin,[§] Serkan Şen,[¶] Ayhan Elmalı,[ζ] Ahmet Karatay,[*,ζ] Muhammet Erkan Köse,[*,ξ] Anthony Harriman,[*,‡] and Özgür Altan Bozdemir[*,†]

[§]Department of Metallurgical Program, Aşkale Vocational College, Ataturk University, 25500, Erzurum, Turkey. [¶]Department of Chemistry, Faculty of Science, Ordu University, 52200, Ordu, Turkey. [ζ]Department of Physics Engineering, Ankara University, 06100, Ankara, Turkey. [ξ]Department of Chemistry, Kocaeli University, Izmit, 41001, Kocaeli, Turkey. [‡]Molecular Photonics Laboratory, Bedson Building, Newcastle University, Newcastle upon Tyne, NE1 7RU, UK. [†]Department of Chemistry, Atatürk University, 25240, Erzurum, Turkey.

SUPPORTING INFORMATION

Total number of pages: 80

Table of contents:

S1. General methods S2

S2. Optical spectroscopy S6

S3. Solute aggregation S15

S4. Computational studies S27

S5. Ultrafast transient absorption spectroscopy S56

S6. Time-resolved fluorescence S64

S7. Nanosecond transient absorption spectroscopy S67

S8. Charge-transfer state S70

S9. ^{1}H NMR, ^{13}C NMR, and mass spectra S75

S10. References S79

S1. General methods

Samples of **PMIDE-m**, **PMIDE-*m*-d**, and **PMIDE-*p*-d** were synthesized following a reported procedure and fully characterized by NMR and MS .[S1] Absorption and fluorescence spectra, respectively, were recorded with a Hitachi U3310 spectrophotometer and a Hitachi F4500 spectrofluorimeter. All measurements were made at 20°C using quartz cells. Absorption spectra were corrected for any nonlinearity in the baseline while emission spectra were corrected for any wavelength dependence. Excitation spectra were recorded with the Hitachi F4500. The absorption spectra were recorded in terms of wavelength (λ) and converted to wavenumber (ν) before being being reduced according to Equation S1.[S2] Here, ε(ν) refers to the molar absorption coefficient as a function of wavenumber. The corresponding fluorescence spectra were likewise recorded in nanometers and converted to wavenumbers. In this case, the reduced spectra were determined according to Equation S2 where F(ν) refers to the fluorescence intensity as a function of wavenumber. The molar absorption coefficient was measured for weighted samples of solute dissolved in a fixed volume of THF. The solution was subjected to mild sonication before being passed through a membrane filter.

$$A(\nu) = \frac{\epsilon(\nu)}{\nu} \qquad (S1)$$

$$E(\nu) = \frac{F(\nu)}{\nu^3} \qquad S(2)$$

The reduced optical spectra were deconstructed into Gaussian components in order to expose the underlying vibronic patterns. The minimum number of Gaussian peaks needed to fully represent the entire spectrum was used. The full-width at half-maximum (FWHM) was kept constant for each analysis but was adjusted to give the best statistical fit according to the quality of the weighted residuals. Analysis was made with the commercial software package PEAKFIT.™ Fluorescence quantum yields were determined for dilute (e.g., 10^{-7} M) solutions using a StellaNet IS6 integrating sphere. Excitation was provided by a stabilized LED emitting at 470 nm. The protocol recommended by Beeby *et al*.[S3] was followed. Fluorescence lifetimes were measured with a PTI EasyLife instrument operated in the time-correlated, single photon counting mode. Excitation was provided by a high-intensity, pulsed LED emitting at 470 nm. Fluorescence was isolated from scattered excitation light using a 500 nm glass cut-off filter before entering the monochromator. After deconvolution, the time resolution of this setup

was *ca*. 300 ps. Data analysis was made by conventional means,[S4] using the chi-squared parameter (χ^2) and the randomness of the weighted residuals to judge the quality of the fit.[S5]

Nanosecond laser flash photolysis experiments were performed using an Applied Photophysics LK50 instrument operated with a Q-switched Nd:YAG laser (Quantel Brilliant B) running at 355 nm with a repetition rate of 10 Hz and a pulse width of *ca*. 4 ns. The 355-nm output was directed through an OPA to produce pulses at 470 nm. Samples were saturated with dried N_2 for one hour before starting the experiment and were purged continuously throughout data collection. The sample was flowed under gravity through a 1 cm glass cell. The monitoring beam was provided by a pulsed Xe lamp filtered to remove UV light. Kinetic measurements were made at fixed wavelengths, with 100 individual laser shots being averaged before analysis. Spectral measurements were made using the point-by-point method, with 5 individual laser shots being averaged at each wavelength.[S6]

A preliminary account of self-association of these PMIDE derivatives has appeared in the literature.[S1] For these studies, absorption spectrometry was performed using a Shimadzu spectrophotometer while steady-state fluorescence measurements were conducted using a Shimadzu RF-5301PC spectrofluorometer. Additional fluorescence studies were made with a variable path length quartz cell[S7] capable of providing optical pathways in the region of 20 microns. This setup was used to check for self-absorption at high concentration. Absorption spectral features for intermolecular dimers were extracted from the mixtures using the commercial software SPECFIT.™ For the bichromophores, the concentration dependence observed for the absorption spectra was analyzed by conventional methods[S8] in order to estimate the fraction of aggregate present under conditions used for the transient absorption spectroscopy. Since it was not possible to operate at high solute concentrations with our fs laser spectrometer setup, aggregation of the bichromophores is restricted to dimerization. As such, the absorbance at any wavelength can be expressed in the form of Equation S3, where C_T is the molar concentration of the solute. By plotting the measured absorbance ($A(\lambda)$) as a function of dye concentration at any wavelength, the molar absorptivities ($\varepsilon(\lambda)M$) of the bichromophore and the corresponding dimer ($\varepsilon(\lambda)D$), as well as K_2, were calculated using a non-linear, least-squares fitting routine.

$$A(\lambda) = \varepsilon_D(\lambda)\left[\frac{C_T}{2} - \frac{-1 \pm \sqrt{1+8K_2C_T}}{8K_2}\right] + \varepsilon_M(\lambda)\left[\frac{C_T}{2} - \frac{-1 \pm \sqrt{1+8K_2C_T}}{4K_2}\right] \qquad (S3)$$

The mol fraction of dimer (α_D) can be estimated quantitatively from Equation S4 while the percentage of incident excitation light (F_D) absorbed by the dimer is given by Equation S5.

$$\alpha_D = 1 - \frac{2K_2C_T + 1 - \sqrt{4K_2C_T + 1}}{2K_2^2C_T^2} \qquad (S4)$$

$$F_D = \frac{\alpha_D\varepsilon_D}{\alpha_D\varepsilon_D + \varepsilon_M(1-\alpha_D)} \qquad (S5)$$

Femtosecond transient absorption spectroscopy measurements were carried out in both acetonitrile and THF solutions. A Ti: Sapphire laser amplifier optical parametric amplifier system with 52 fs pulse duration and 1 kHz repetition rate (Spectra Physics, Spitfire Pro XP, TOPAS) and a commercial pump probe experimental setup (Spectra Physics, Helios) with a white light continuum probe was used for the experiments. Pulse duration was measured as 120 fs by cross-correlation inside the pump probe setup. The wavelength of the pump beam was chosen as 500 nm according to the steady-state absorption spectra of studied compounds. In most cases, the absorbance of the solution at 500 nm was adjusted to be 1.0. In certain experiments, the solute concentration was varied over a narrow range in order to examine the effects of aggregation on the transient spectra. Experimental data were analyzed using Surface Xplorer software.™

Differential pulse voltammetry experiments were made with **PMIDE-p-d** in freshly distilled CH_2Cl_2 after displacement of the dissolved air with argon. The solute concentration was *ca*. 0.5 mM and the solution contained tetrabutylammonium hexafluorophosphate (0.1 M) as supporting electrolyte. The electrochemical cell consisted of a glassy carbon working electrode, a platinum wire counter electrode, and an Ag-wire as *pseudo* reference electrode driven by an HCH Instruments Electrochemical Analyzer. The subsequent voltammograms were referenced to an internal ferrocene/ferrocenium (Fc/Fc^+) redox couple and were recorded for both positive and negative sweeps. The working electrode was polished between scans using 2 µM alumina paste. Spectroelectrochemical studies were made with **PMIDE-p-d** (1 mM) in deoxygenated MeCN containing $TBAPF_6$ (0.1 M) using a Perkin–Elmer Lambda 35 spectrophotometer with the aid of an optically transparent thin-layer electrochemical cell

(OTTLE) supplied by SpecAc. The OTTLE consisted of a platinum-mesh working electrode, a platinum-wire counter electrode, and a silver wire reference electrode and was controlled by the HCH Instruments Electrochemical Analyzer. Solutions were deoxygenated by purging with nitrogen for fifteen minutes prior to the experiment. Potentials were switched after electrolysis in order to confirm the full reversibility of the system.

S2. Optical spectroscopy

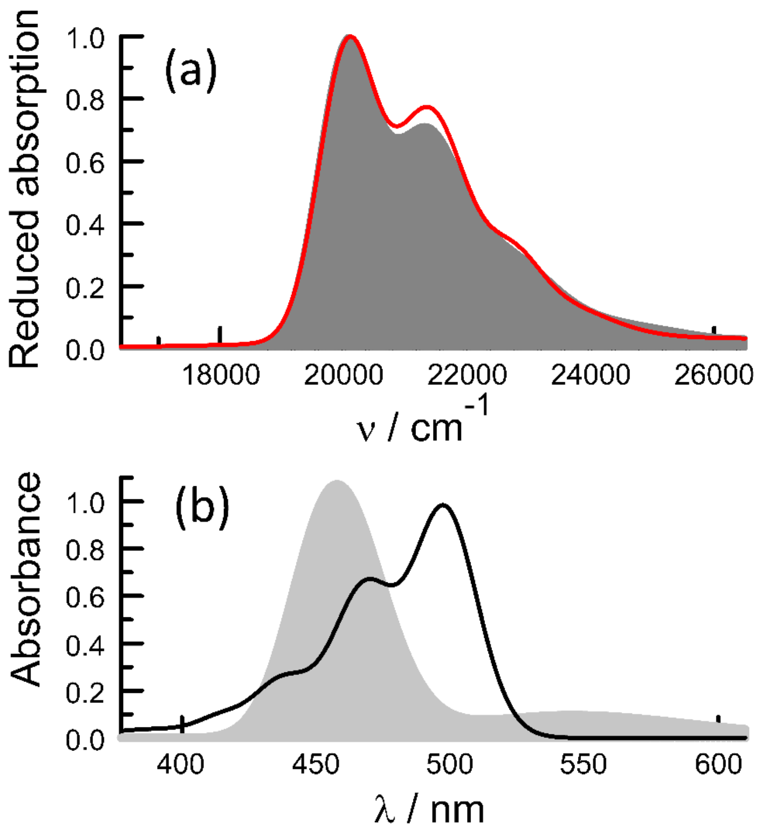

Figure S1. (a) Comparison of reduced absorption spectra recorded at low concentration (i.e., *ca*. 2×10^{-7} M) shown as a red curve and high concentration (i.e., 2×10^{-5} M) shown as a shaded curve. The sample was **PMIDE-m** dissolved in MeCN. The close similarity is taken to indicate the relative unimportance of aggregation in this solvent. (b) Normalized absorption spectra for the monomer (*ca*. 2×10^{-7} M, black curve) and dimer (5×10^{-4} M, shaded curve) for **PMIDE-m** in MeCN at room temperature. For the latter spectrum, the contribution from residual monomer was deleted by spectral curve fitting routines. In both cases, spectra are normalized at the respective maxima.

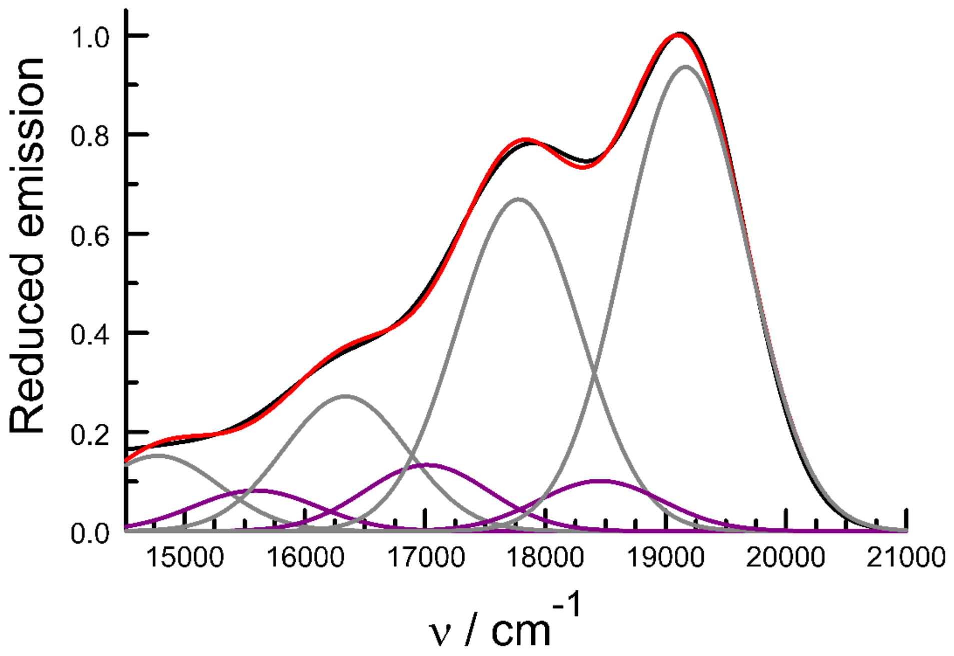

Figure S2. Example of output from the Gaussian deconstruction procedure applied to the reduced fluorescence spectrum recorded for **PMIDE-m** in MeCN. The experimental spectrum is shown as a black curve with the simulated spectrum appearing as a red curve. Individual Gaussian components correspond to a medium-frequency mode (grey curves) or to a low-frequency mode (purple curves). The 0,0 transition and the vibration spacing are derived from the statistical fit. The FWHM is kept constant.

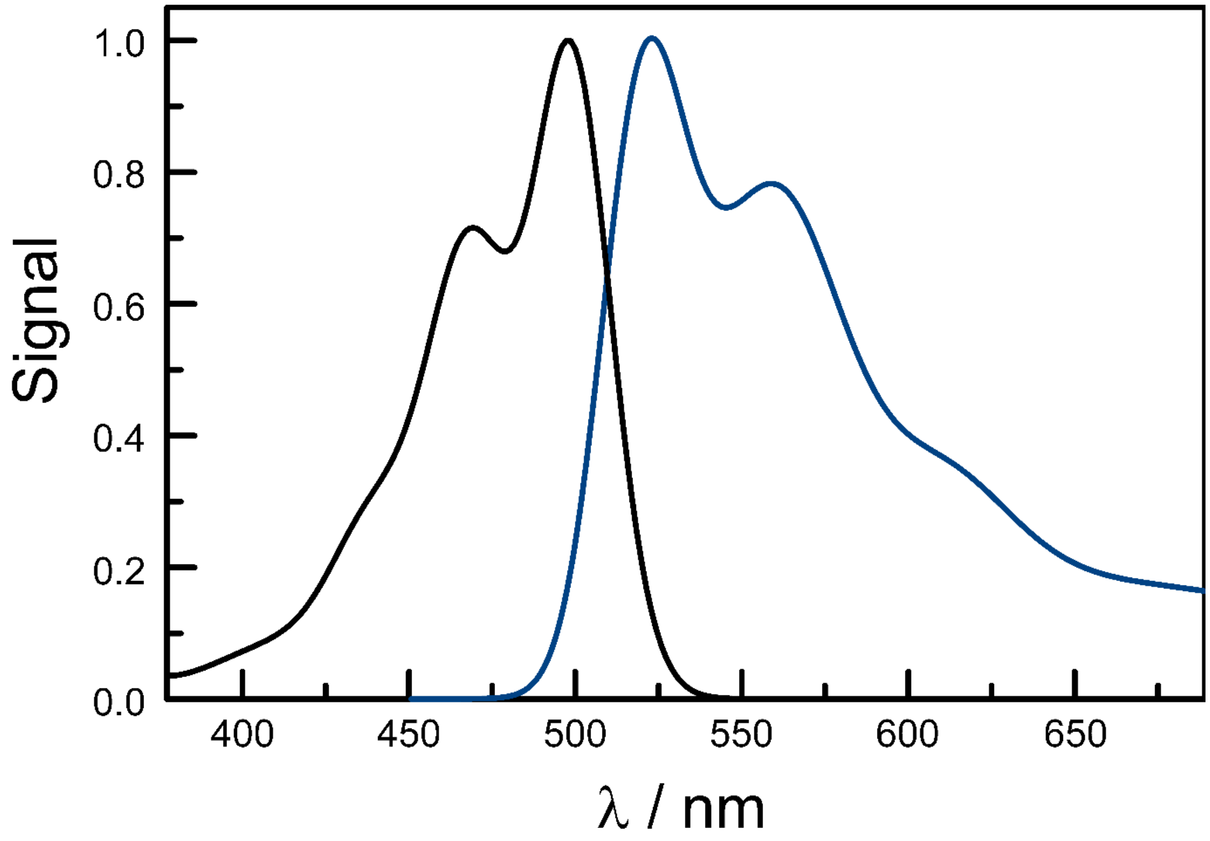

Figure S3. Absorption (black curve) and fluorescence (blue curve) spectra recorded for **PMIDE-m** in MeCN at room temperature. The spectra are normalized at the respective maxima. The solute concentration was ca. 2×10^{-7} M and the excitation wavelength was 440 nm.

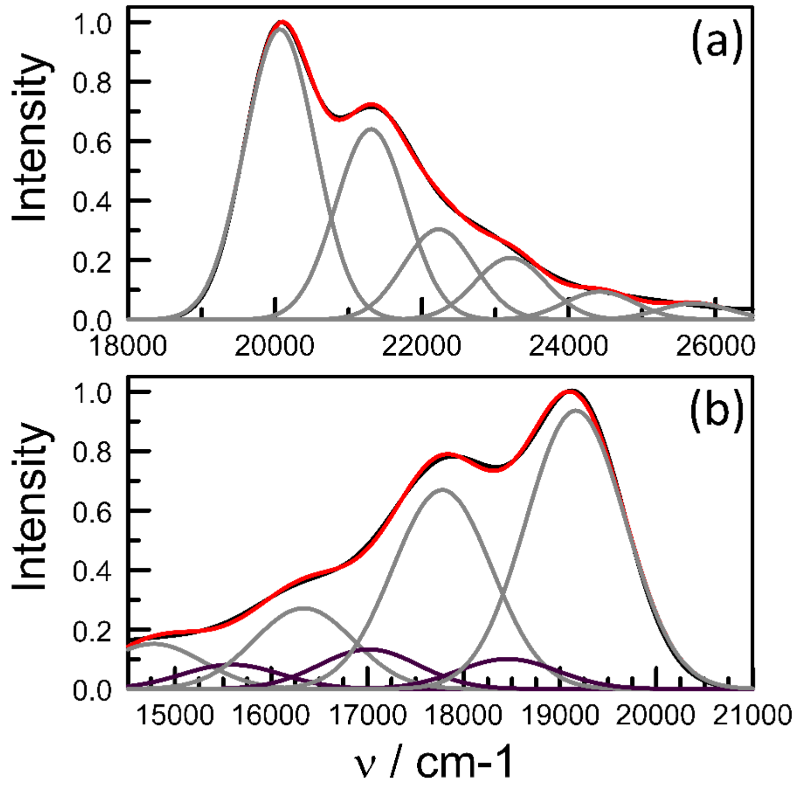

Figure S4. Comparison of (a) reduced absorption and (b) reduced fluorescence spectra recorded for dilute solutions of **PMIDE-m** in MeCN. In each case, the experimental spectrum is shown as a black curve while the simulated spectrum is given as a red curve. The individual Gaussian components are illustrated as grey curves. For the fluorescence spectrum, the low-frequency vibronic modes needed to effect a good statistical fit are shown in purple.

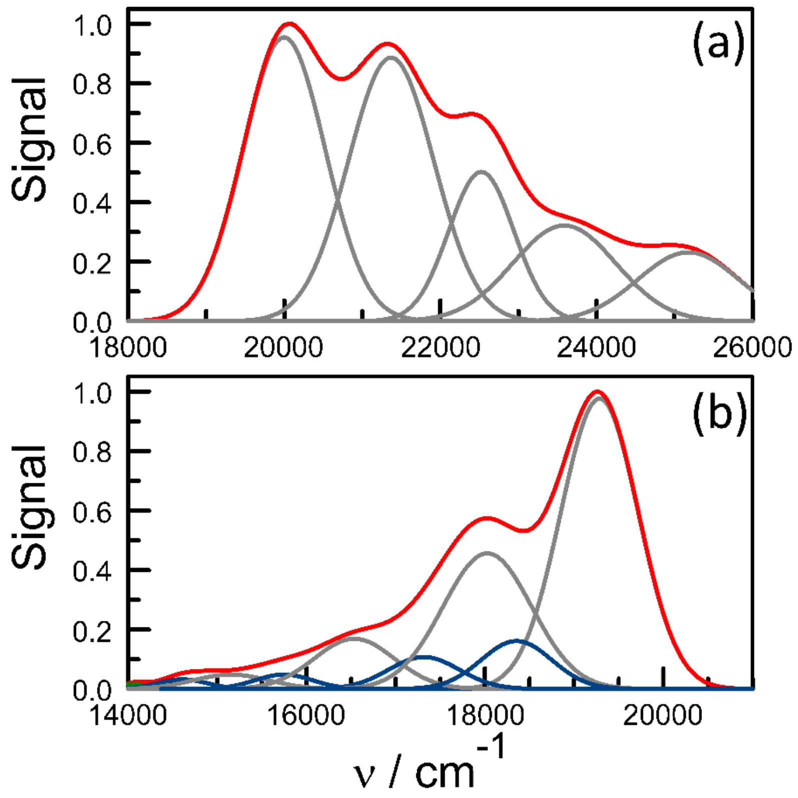

Figure S5. Comparison of (a) reduced absorption and (b) reduced fluorescence spectra recorded for dilute solutions of **PMIDE-m** in THF. In each case, the experimental spectrum is shown as a black curve while the simulated spectrum is given as a red curve. The individual Gaussian components are illustrated as grey curves. For the fluorescence spectrum, the low-frequency vibronic modes needed to effect a good statistical fit are shown in blue.

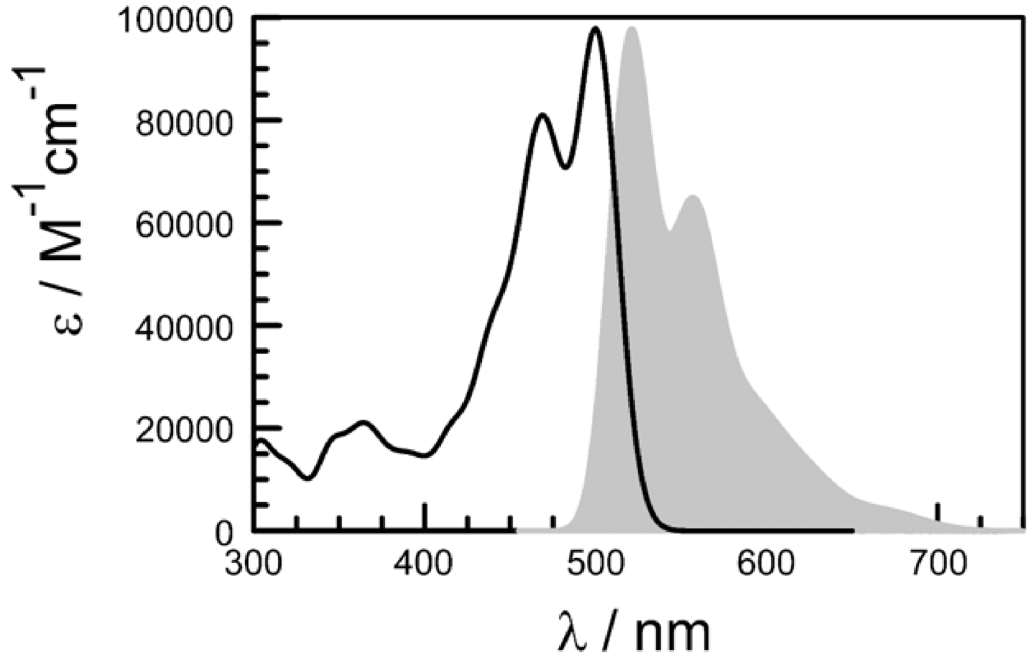

Figure S6. Examples of absorption (black curve) and fluorescence (shaded curve) spectra recorded for **PMIDE-*m*-d** in THF at room temperature. The spectra are normalized at the respective maxima and the solute concentration was *ca*. 5×10^{-7} M. The excitation wavelength used for the fluorescence experiment was 450 nm and a glass cut-off filter was used to help eliminate scattered excitation light from emission. Note the significant overlap, despite the modest Stokes shift, which strongly favors self-absorption at higher concentrations.

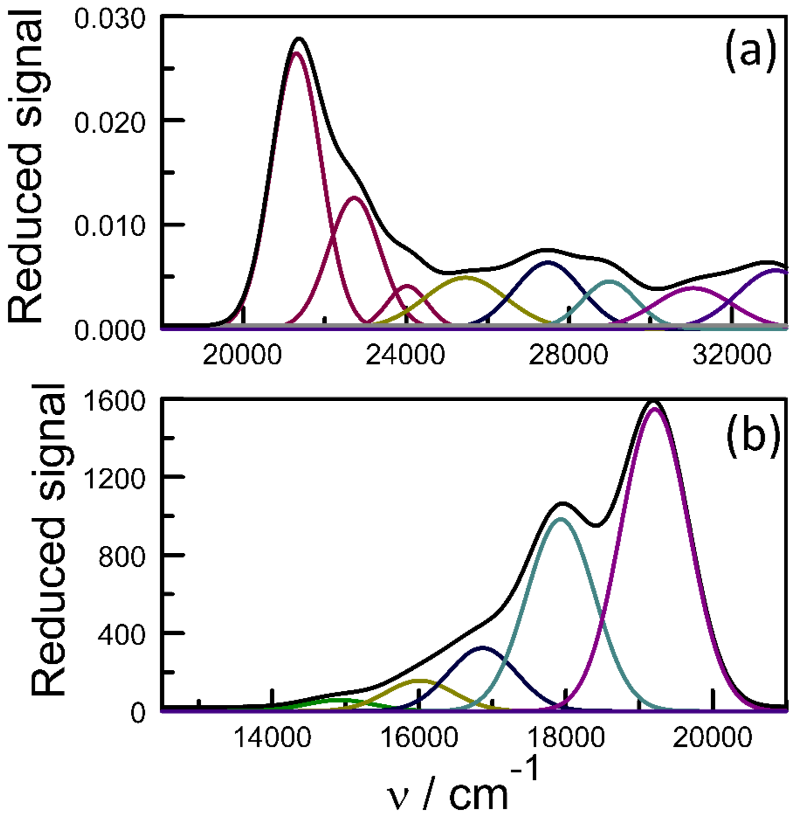

Figure S7. Examples of the Gaussian deconstruction used to determine the 0,0 transitions for (a) absorption and (b) fluorescence spectra recorded for **PMIDE-*m*-d** in MeCN. The spectra were reduced before fitting.

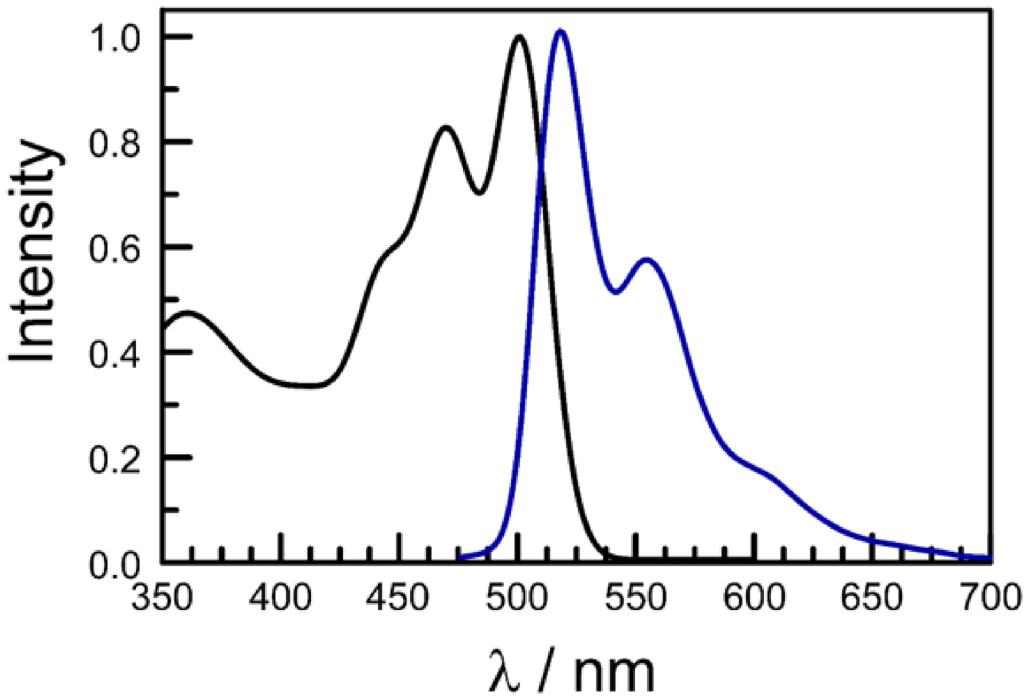

Figure S8. Overlay of normalized absorption (black curve) and fluorescence (blue curve) spectra recorded for **PMIDE-*p*-d** in MeCN at room temperature. The solute concentration was *ca*. 5×10^{-7} M.

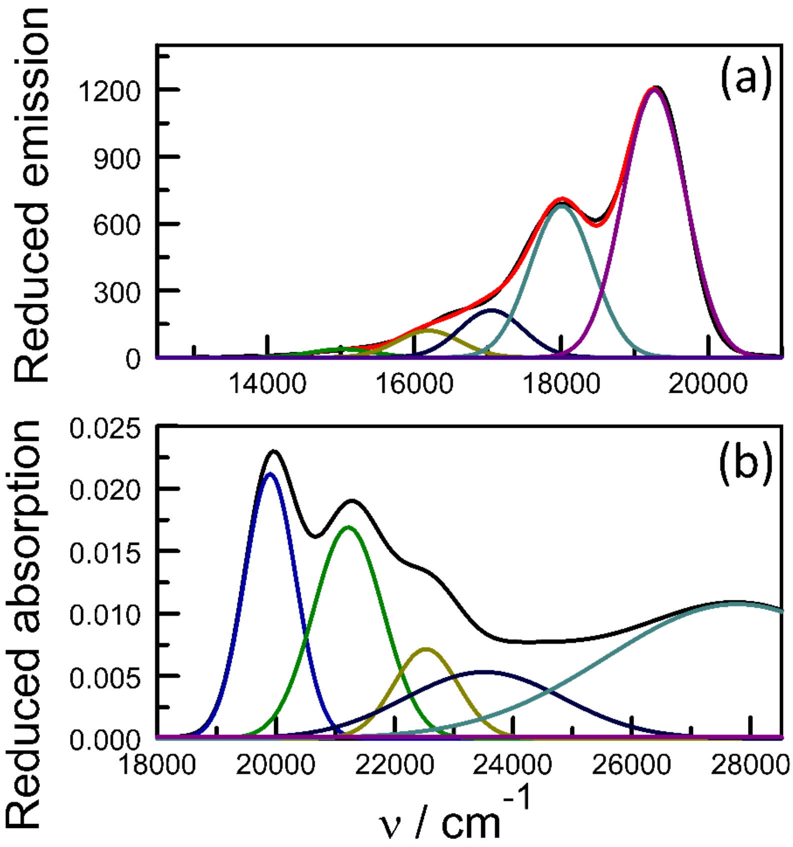

Figure S9. Examples of the Gaussian deconstruction used to determine the 0,0 transitions for (a) fluorescence and (b) absorption spectra recorded for **PMIDE-*p*-d** in MeCN. The spectra were reduced before fitting.

S3. Solute aggregation

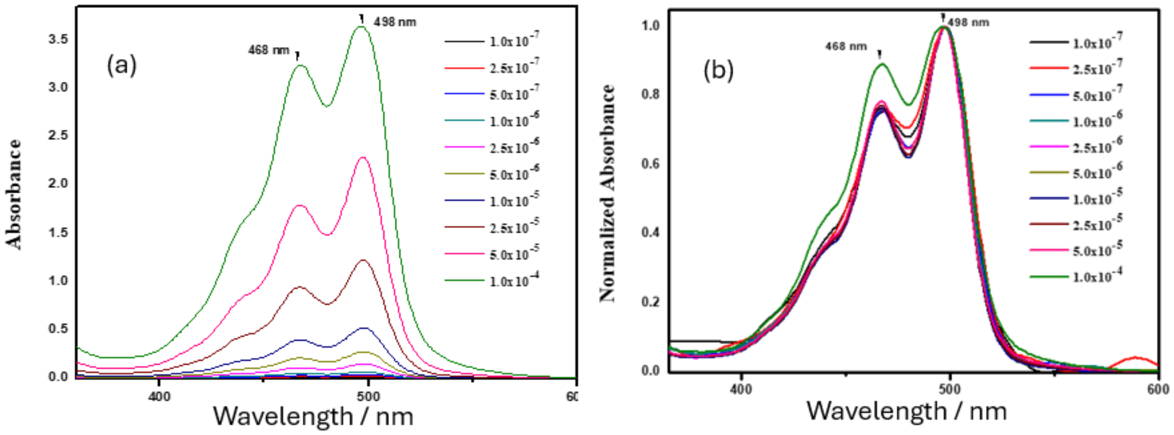

Figure S10. (a) The effect of increasing solute concentration on the absorption spectral profile for **PMIDE-m** in MeCN. (b) The same data but with the absorbance normalized at 498 nm. Molar concentrations are given on the figure. A range of optical pathlengths was used.

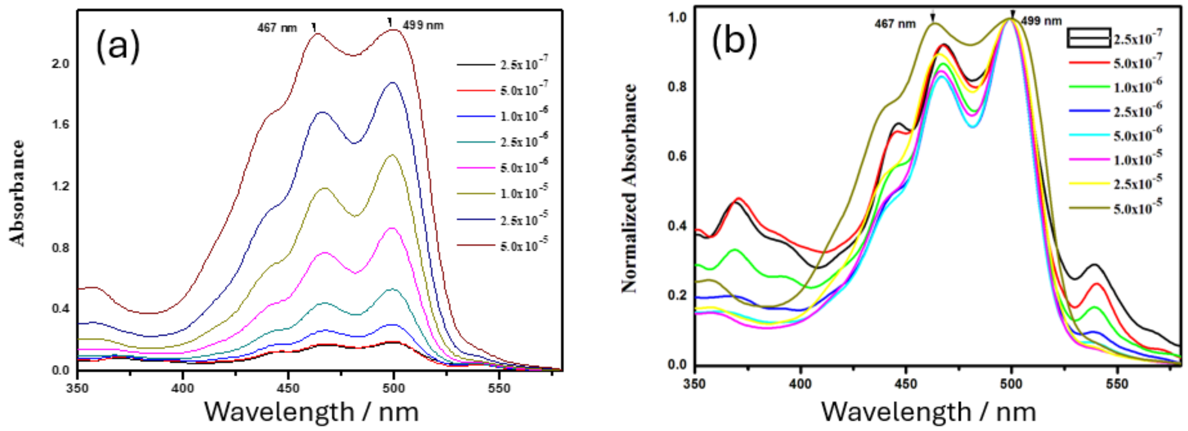

Figure S11. (a) The effect of increasing solute concentration on the absorption spectral profile for **PMIDE-m** in THF. (b) The same data but with the absorbance normalized at 499 nm. Molar concentrations are indicated on the figure next to the color coding.

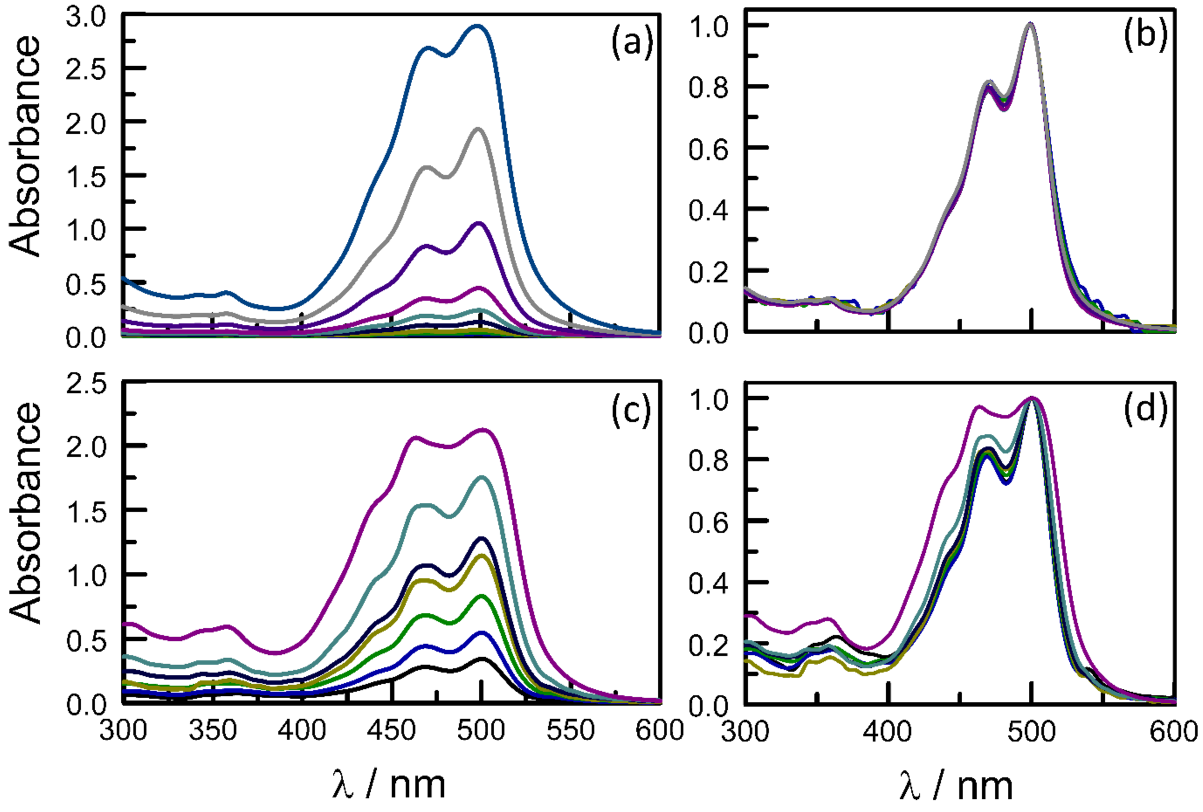

Figure S12. Effect of increasing solute concentration on the absorption spectral profile recorded for **PMIDE-*m*-d** in (a) MeCN and (b) the same data presented with the absorbance normalized at 501 nm. (c) Effect of increasing solute concentration on the absorption spectral profile recorded for **PMIDE-*m*-d** in THF and (d) the same data presented with the absorbance normalized at 501 nm. Molar concentrations range from 1×10^{-7} M to 1×10^{-4} M. The same color scheme applies to regular spectra and normalized spectra.

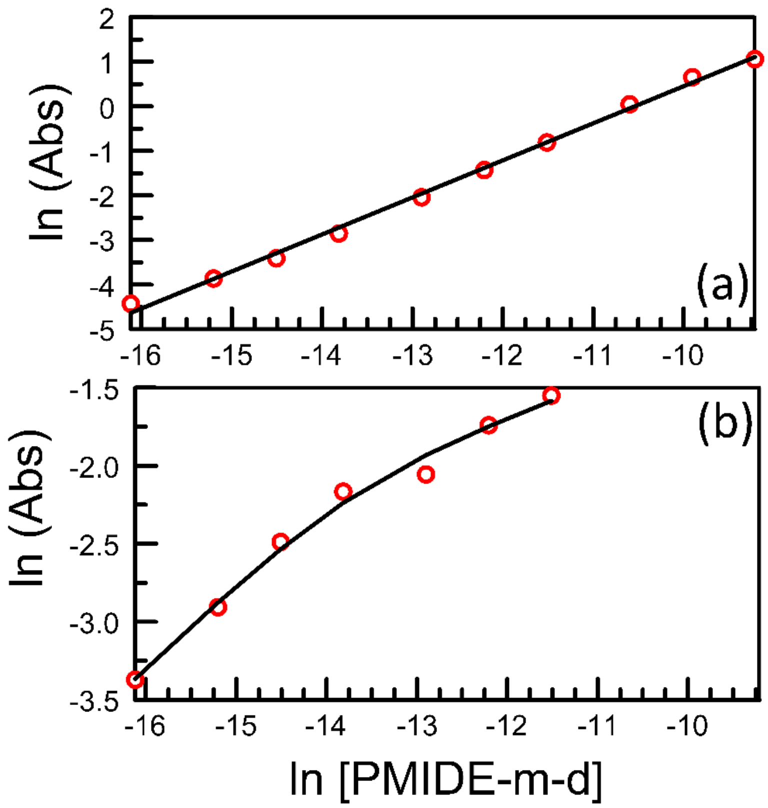

Figure S13. Relationship between the absorbance at 500 nm and the total solute concentration for **PMIDE-*m*-d** in (a) MeCN and (b) THF at room temperature. The data are shown in the form of a ln-ln plot. For MeCN ($K_2 = 2.6 \pm 1.0 \times 10^2$ M^{-1}), there is little aggregation under these conditions. For THF, the fit to Equation S3 gives an approximate value for K_2 of 6.7 $\pm 1.0 \times 10^2$ M^{-1}.

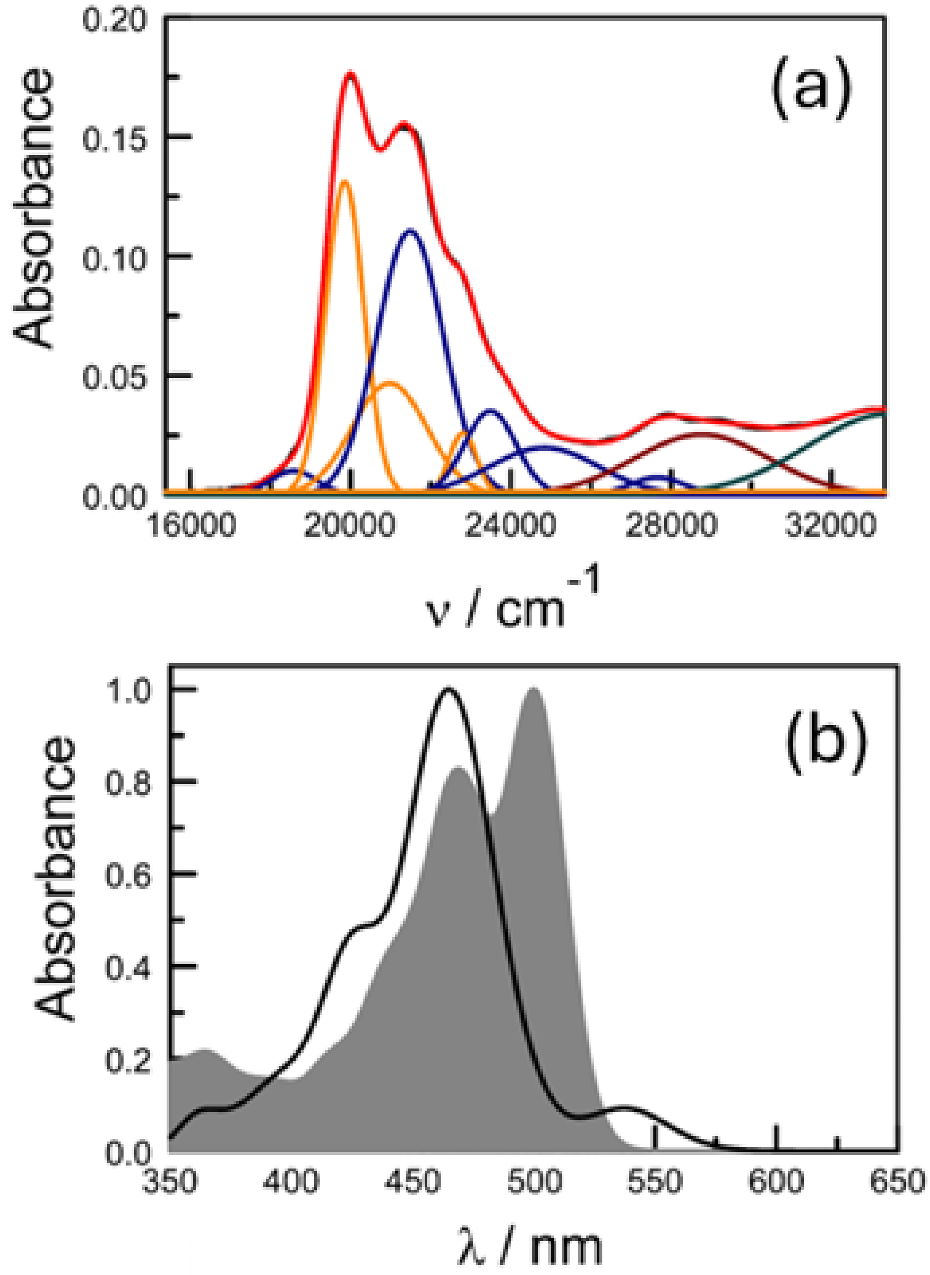

Figure S14. (a) Spectral deconstruction of the absorption profile recorded for **PMIDE-*m*-d** in THF (5×10^{-4} M) to separate transitions associated with the monomer (orange curves) and dimer (blue curves). Transitions to higher-energy states of the monomer are shown in brown and green. The transitions due to the dimer, seen only at high concentration, are summed to give the final absorption spectrum of the dimer. (b) Absorption spectrum derived for the intermolecular dimer formed at high concentrations of **PMIDE-*m*-d** in THF. The dimer spectrum (black curve) is superimposed over the spectrum of the monomer (grey curve). Absorption peaks for the dimer are at 539 and 465 nm. The spectra are normalized at the peak maxima.

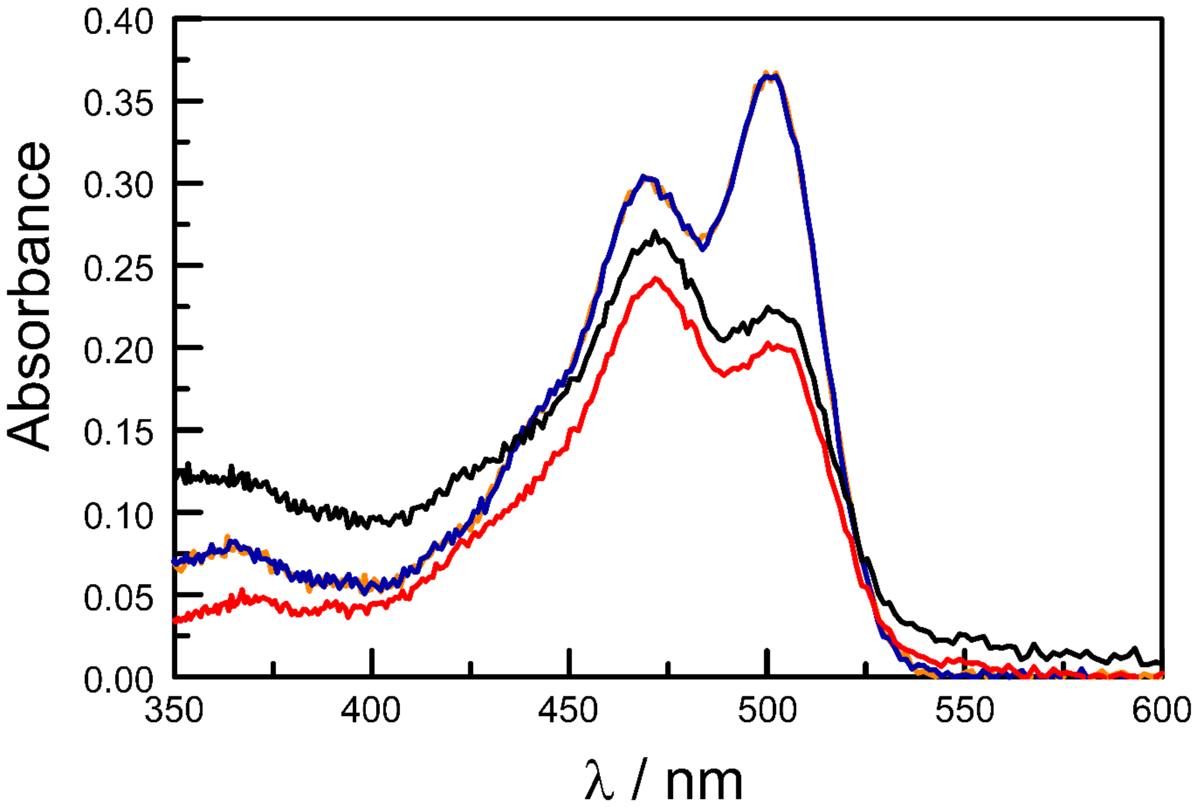

Figure S15. Absorption spectrum recorded for **PMIDE-m-d** (concentration 40 µM) in 2-methyltetrahydrofuran at 170K (black curve), The corresponding spectrum after elimination of the background scatter is shown as a red curve. The spectra recorded at room temperature before cooling (blue curve) and after returning to room temperature (orange curve) are also shown. For the experiment, the solution was placed between two glass circular plates fitted with a teflon spacer giving a pathlength of *ca*. 2 mm and sealed with quick-drying epoxy cement. The cell was placed in an Oxford Instruments Optistat DN cryostat and cooled slowly to 170K, where the solvent is still fluid. After 1 hr standing, the absorption spectrum was recorded using optical fibres to interface to the spectrophotometer.

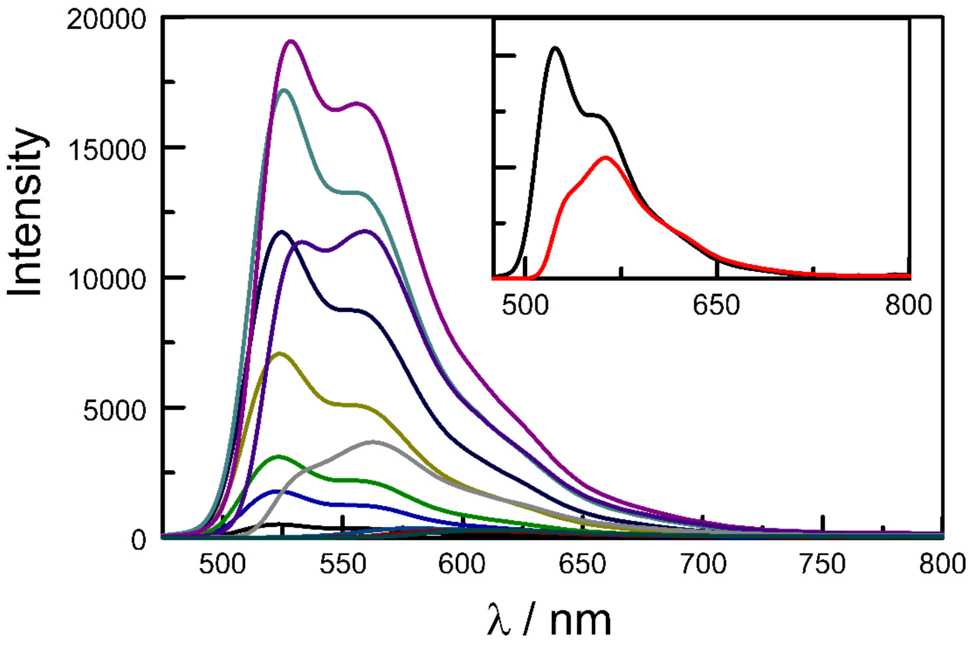

Figure S16. Effect of solute concentration on the fluorescence intensity for **PMIDE-*m*-d** in MeCN at room temperature. The excitation wavelength was 440 nm. The integrated yield increases linearly with increasing concentration until reaching a plateau. Further increases in concentration lead to a decrease in emission yield, in accord with the inner filter effect. The insert compares emission spectra recorded at solute concentrations of 1 µM (black curve) and 1×10^{-4} M (red curve). The observed nonlinearity is due to self-absorption (i.e., the inner filter effect) at higher concentrations. The concentration range is from 1×10^{-7} M to 5×10^{-4} M.

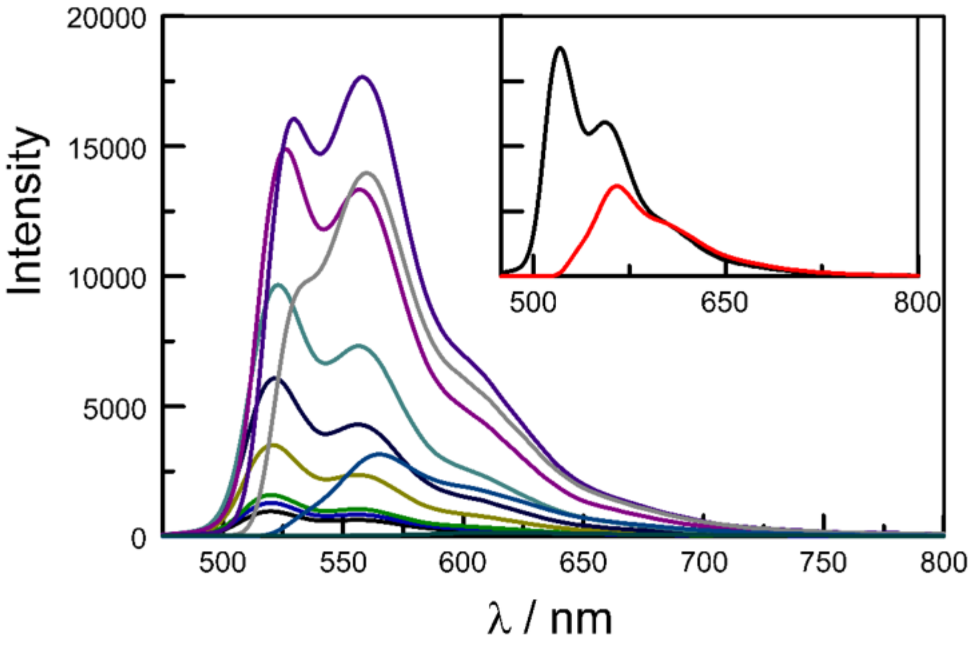

Figure S17. Effect of solute concentration on the fluorescence intensity for **PMIDE-*m*-d** in THF at room temperature. The excitation wavelength was 440 nm. The integrated yield increases linearly with increasing concentration until reaching a plateau. Further increases in concentration lead to a decrease in emission yield, in accord with the inner filter effect. The insert compares emission spectra recorded at solute concentrations of 1 µM (black curve) and 2.5×10^{-4} M (red curve). The observed nonlinearity is due to self-absorption (i.e., the inner filter effect) at higher concentrations. The concentration range is from 1×10^{-7} M to 5×10^{-4} M.

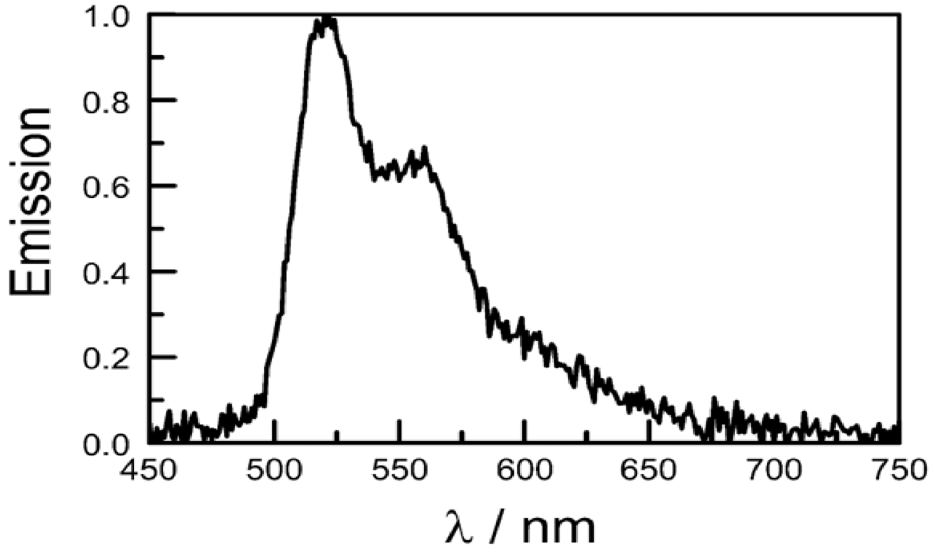

Figure S18. Example of a fluorescence spectrum recorded for **PMIDE-*m*-d** in THF solution at a solute concentration of 2.5×10^{-4} M using a variable path length cell. The cell was adjusted to give an absorbance of 0.05 at the excitation wavelength of 440 nm. The characteristic spectrum of the monomer is recovered under these conditions because of the absence of self-absorption.

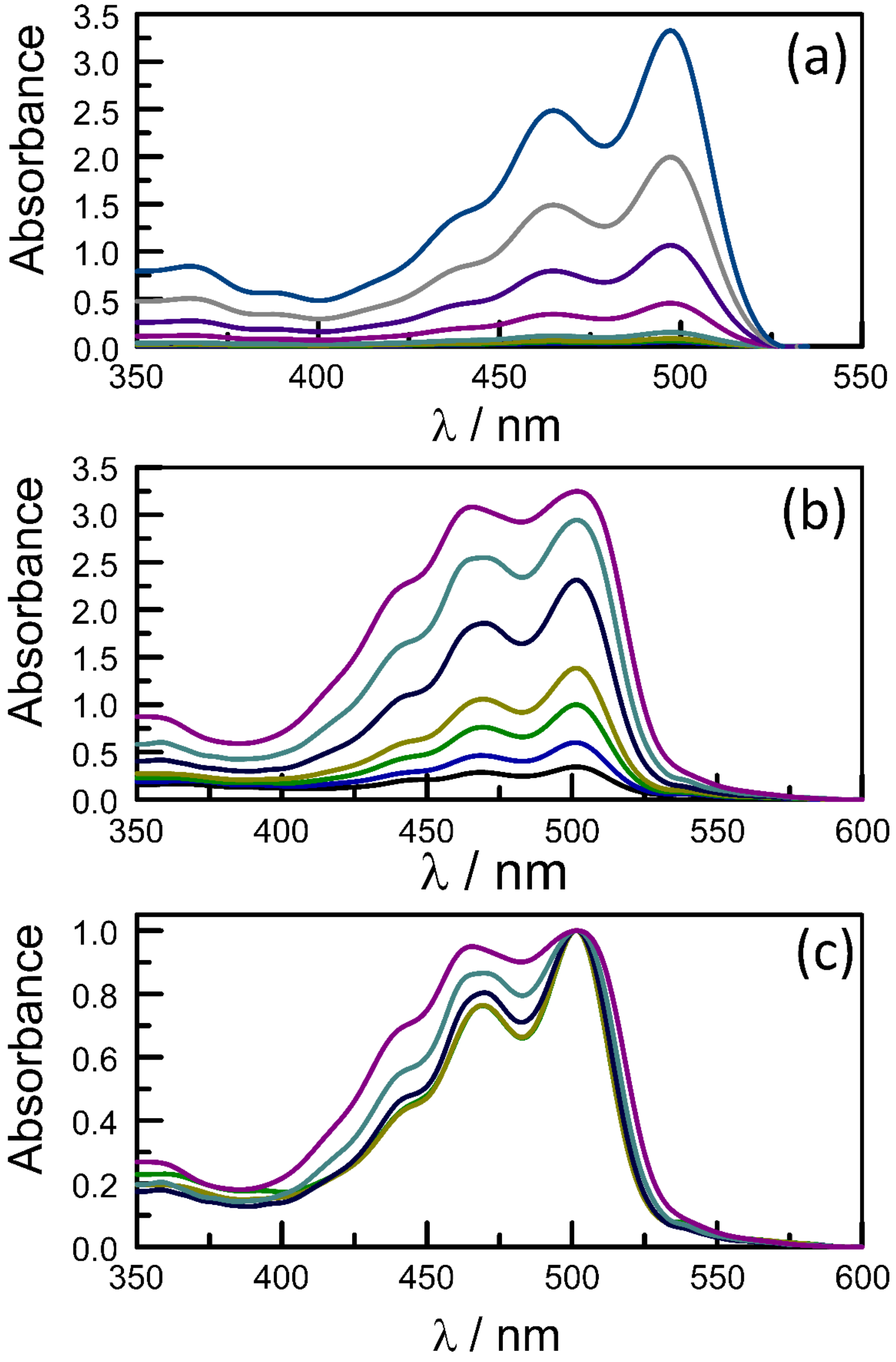

Figure S19. Effect of increasing solute concentration on the absorption spectral profile recorded for **PMIDE-*p*-d** in (a) MeCN and (b) THF at room temperature. Molar concentrations range from 1×10^{-7} M to 1×10^{-4} M for MeCN and from 1×10^{-7} M to 1×10^{-4} M for THF. (c) Data obtained for **PMIDE-p-d** in THF presented after normalization at 600 nm, concentrating on the high concentration region and retaining the color scheme used in panel (b).

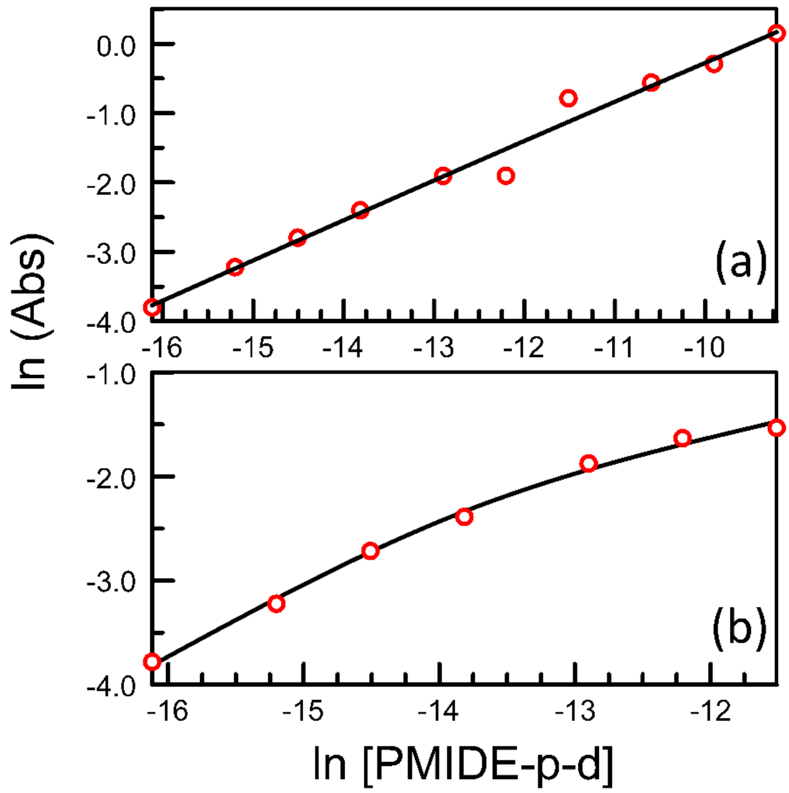

Figure S20. Relationship between the absorbance at 500 nm and the total solute concentration for **PMIDE-*p*-d** in (a) MeCN and (b) THF at room temperature. The data are shown in the form of a ln-ln plot. For MeCN, there is only a small degree of aggregation under these conditions, corresponding to a K_2 value of $1.6 \pm 0.6 \times 10^3$ M^{-1}. For THF, the fit to Equation S3 gives an approximate value for K_2 of $8 \pm 2 \times 10^3$ M^{-1}.

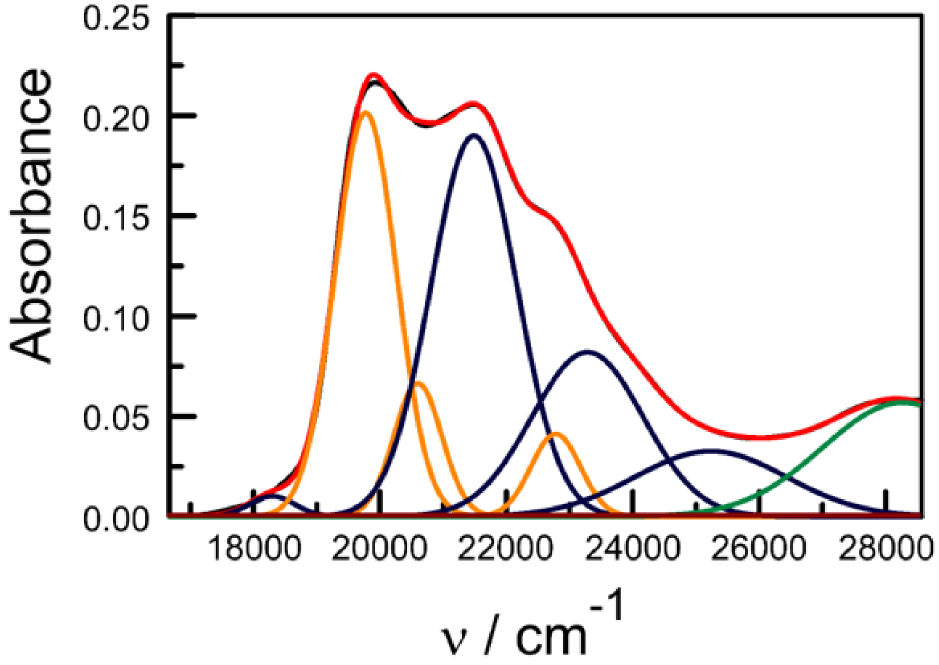

Figure S21. Spectral deconstruction of the absorption profile recorded for **PMIDE-p-d** in THF (5×10^{-4} M) to separate transitions associated with the monomer (orange curves) and dimer (blue curves). Transition to a higher-energy state of the monomer is shown in green. The transitions due to the dimer, seen only at high concentration, are summed to give the final absorption spectrum of the dimer.

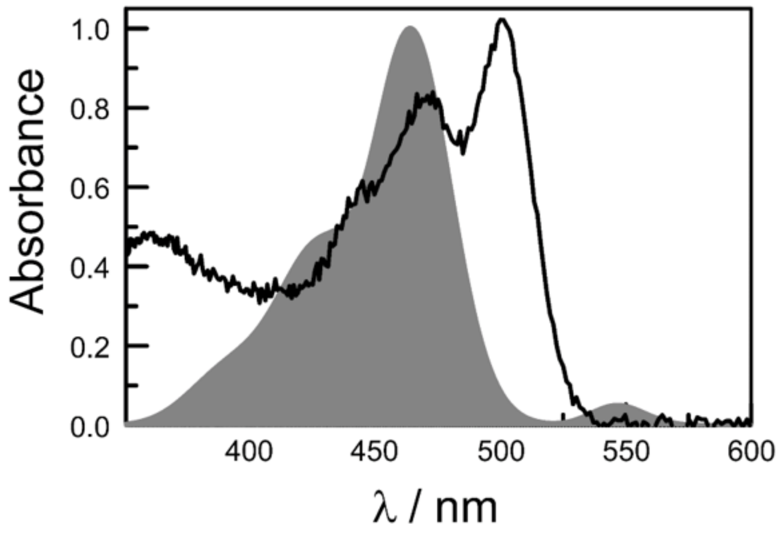

Figure S22. Absorption spectrum derived for the intermolecular dimer formed at high concentrations of **PMIDE-*p*-d** in THF. The dimer spectrum (shaded curve) is superimposed over the spectrum of the monomer (black curve). Absorption peaks for the dimer are at 547 and 464 nm. The spectra have been normalized at their respective maxima.

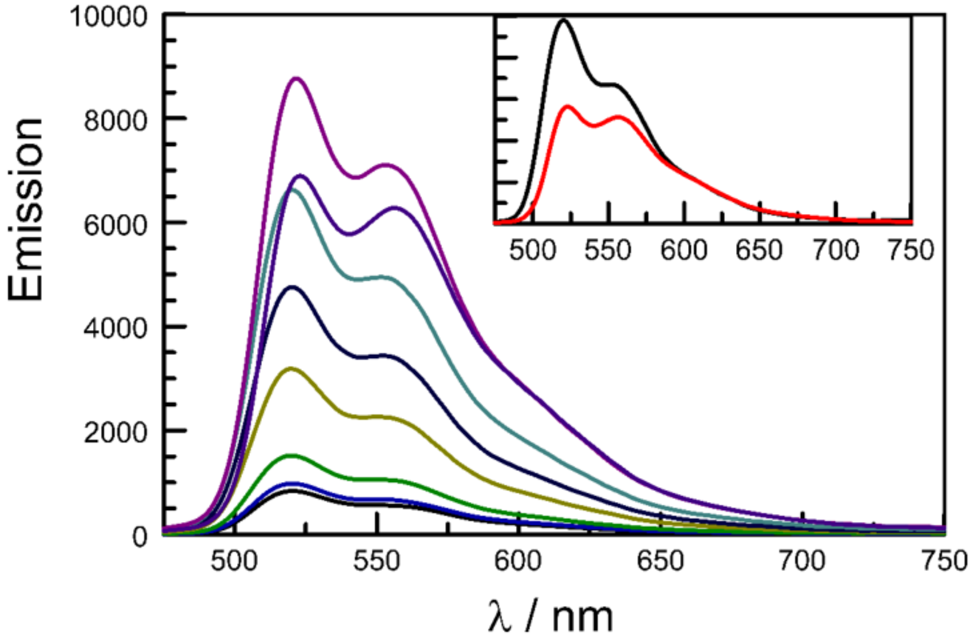

Figure S23. Effect of solute concentration on the fluorescence intensity for **PMIDE-*p*-d** in MeCN at room temperature. The excitation wavelength was 440 nm. The integrated yield increases linearly with increasing concentration until reaching a plateau. Further increases in concentration lead to a decrease in emission yield, in accord with the inner filter effect. The insert compares emission spectra recorded at solute concentrations of 1 μM (black curve) and 1×10^{-4} M (red curve). The observed nonlinearity is due to self-absorption (i.e., the inner filter effect) at higher concentrations. The concentration range is from 1×10^{-7} M to 5×10^{-4} M.

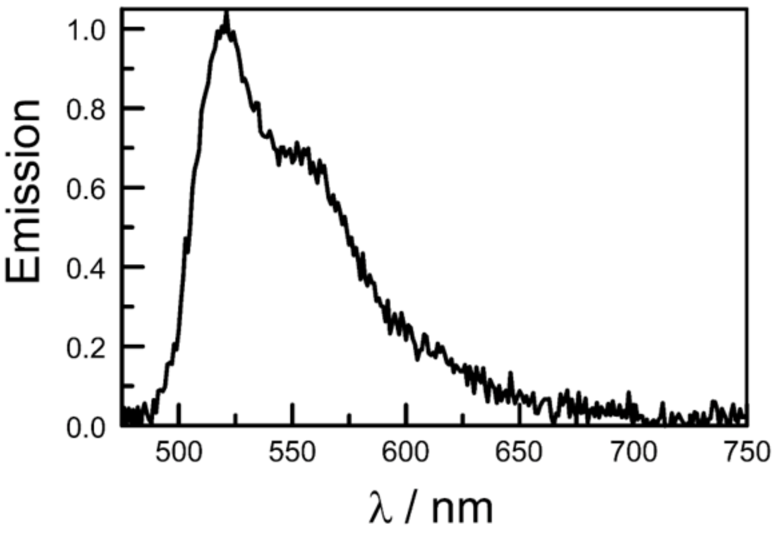

Figure S24. Example of a fluorescence spectrum recorded for **PMIDE-*p*-d** in MeCN solution at a solute concentration of 2.5×10^{-4} M using a variable path length cell. The cell was adjusted to give an absorbance of 0.05 at the excitation wavelength of 440 nm. The characteristic spectrum of the monomer is recovered under these conditions because of the absence of self-absorption.

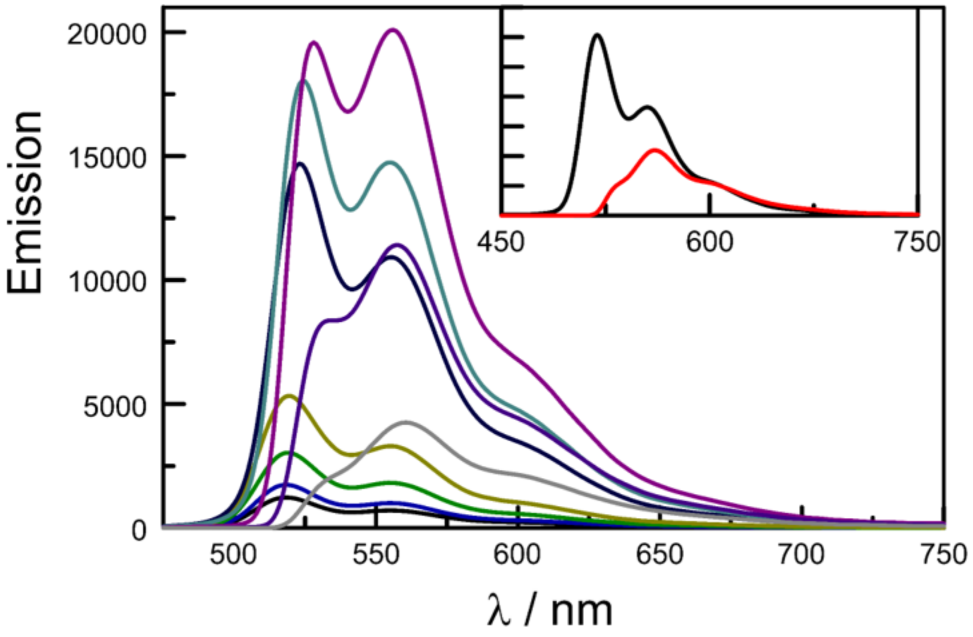

Figure S25. Effect of solute concentration on the fluorescence intensity for **PMIDE-*p*-d** in THF at room temperature. The excitation wavelength was 440 nm. The integrated yield increases linearly with increasing concentration until reaching a plateau. Further increases in concentration lead to a decrease in emission yield, in accord with the inner filter effect. The insert compares emission spectra recorded at solute concentrations of 1 μM (black curve) and 1×10^{-4} M (red curve). The observed nonlinearity is due to self-absorption (i.e., the inner filter effect) at higher concentrations. The concentration range is from 1×10^{-7} M to 5×10^{-4} M.

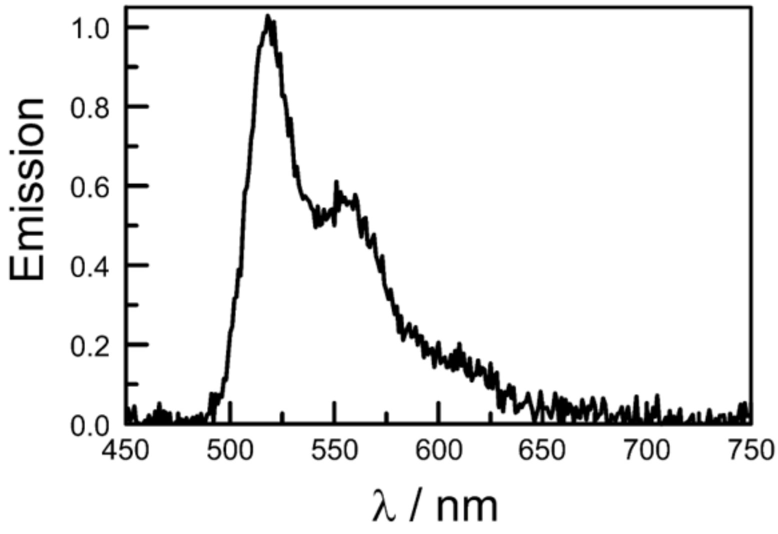

Figure S26. Example of a fluorescence spectrum recorded for **PMIDE-*p*-d** in THF solution at a solute concentration of 2.5×10^{-4} M using a variable path length cell. The cell was adjusted to give an absorbance of 0.05 at the excitation wavelength of 440 nm. The characteristic spectrum of the monomer is recovered under these conditions because of the absence of self-absorption.

S4. Computational studies

Density functional theory (DFT) was performed to reveal the ground state geometries of **PMIDE-m**, **PMIDE-*m*-d**, and **PMIDE-*p*-d**. All geometry optimizations and frequency calculations were carried out using the B3LYP hybrid functional[S9] along with the 6-311G(d,p) basis set.[S10] The absence of imaginary frequencies for the optimized geometries was taken as confirmation of the minima on the potential energy surfaces. Time-dependent density functional theory (TD-DFT) calculations for singlet-excited states were performed with the B3LYP functional, whereas the CAM-B3LYP functional[S11] was exploited to determine the triplet excitation energies on B3LYP-optimized geometries. CAM-B3LYP was chosen to calculate the triplet states since a similar approach for PMI derivatives has been used successfully in a recent study.[S12] These calculations were performed with the Gaussian 09 software package.[S13]

Table S1. Calculated vertical transition energies for **PMIDE-m**, **PMIDE-*m*-d** and **PMIDE-*p*-d** for excited-singlet and excited-triplet states. Oscillator strengths (f) for triplet states are equal to zero.

**Compound**	**S_1 (eV) (f)**	**S_2 (eV) (f)**	**T_1 (eV)**	**T_2 (eV)**	**T_2 (eV)**	**T_4 (eV)**
**PMIDE-m**	2.49 (0.64)	3.09 (0)	1.11	2.71	2.77	3.34
**PMIDE-*m*-d**	2.45 (1.11)	2.51 (0.28)	1.11	1.11	2.71	2.71
**PMIDE-*p*-d**	2.45 (0.89)	2.45 (0.56)	1.11	1.11	2.71	2.71

Optimized ground-state geometries of both **PMIDE-*m*-d** and **PMIDE-*p*-d** show that the central phenyl rings do not lie on the same plane as those attached to the PMIDE groups. The central phenyl group has a torsion angle of 64° for **PMIDE-*m*-d** and 69° for **PMIDE-*p*-d**. Such large twisting angles break the effective conjugation between the attached chromophores and hence inhibit π-electron delocalization throughout the molecule. In addition, the *meta*-connection is expected to prohibit conjugation around the core for conjugated systems. However, such delocalization is possible for the *para*-linkage but, in our case, the large

twisting angle leads to poor electronic communication between the PMIDE groups perylene on the same molecule.

The geometries of both bichromophores have small effects on their photophysical properties as can be clearly seen from the computed optical data. The calculated S_1 and T_1 energy levels for **PMIDE-m** are very similar in magnitude with those of the bichromophores (Table S1). This means that each PMIDE group acts as a distinct chromophore and the central phenyl group serves only as a linker and is not involved in π-electron delocalization. The result is degenerate states for both singlet and triplet excitations. Indeed, frontier molecular orbital plots constructed for S_1 and S_2 states of the bichromophores show that there are no molecular orbital coefficients present on the phenyl linkers (Figure S27). The triplet states appear as multiple transitions with many paired excitations in the TD-DFT output, which renders interpretation somewhat difficult. Therefore, natural transition orbitals (NTOs) were generated from the canonical molecular orbitals in order to get a localized picture of the transition density matrix.[S14] For vertical transitions of the triplet states, these NTOs show that electronic transitions are localized on the PMIDE groups in each molecule (Figures S28 and S29).

Where comparison is possible, the calculated transition energies are in good accord with the experiment. The calculated S_1 excitation energy of **PMIDE-m** is 2.49 eV, in perfect agreement with the experimental result measured in THF. The calculated S_1 excitation energy for **PMIDE-*m*-d** and **PMIDE-*p*-**d is 2.45 eV, again in very good accordance with the experimental findings (2.49 eV and 2.50 eV, respectively). The experimental T_1 excitation energy for a PMIDE bichromophore interconnected with a naphthalene linker has been measured as 1.12 eV by low-temperature phosphorescence spectroscopy.[S12] Although we do not have data on the phosphorescence spectrum of our molecules, it seems reasonable that the T1 excitation energy should be around 1.1 eV. If this would be the case, the calculated T_1 excitation energies (Table S1) are as expected.

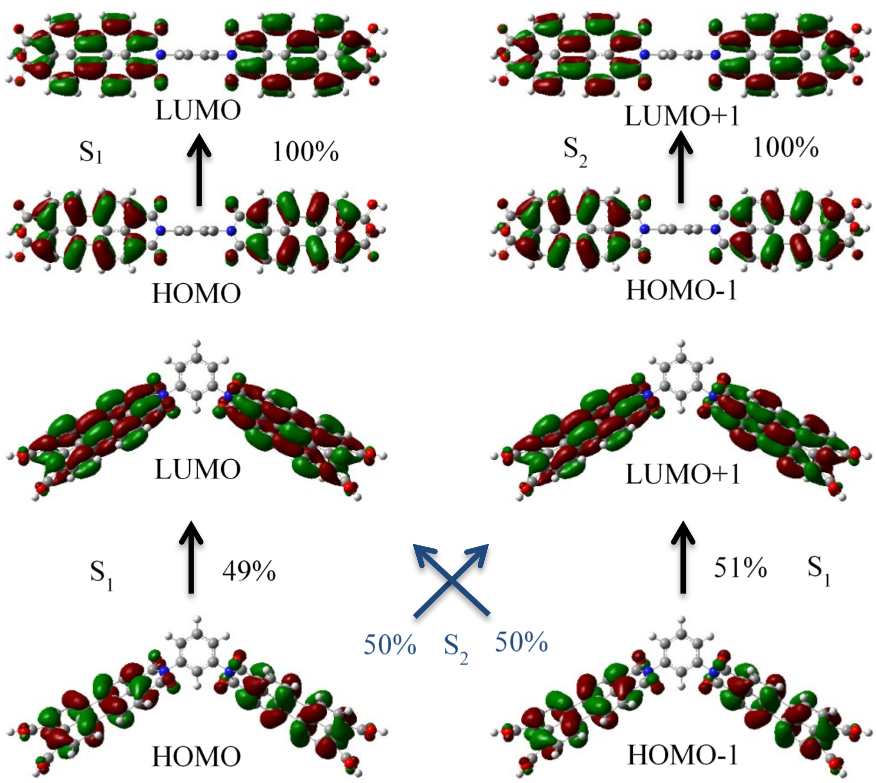

Figure S27. Frontier molecular orbitals for S_1 and S_2 vertical transitions for **PMIDE-*m*-d**, and **PMIDE-*p*-d** are shown with percent contributions from paired singlet excitations. Isovalue = 0.02.

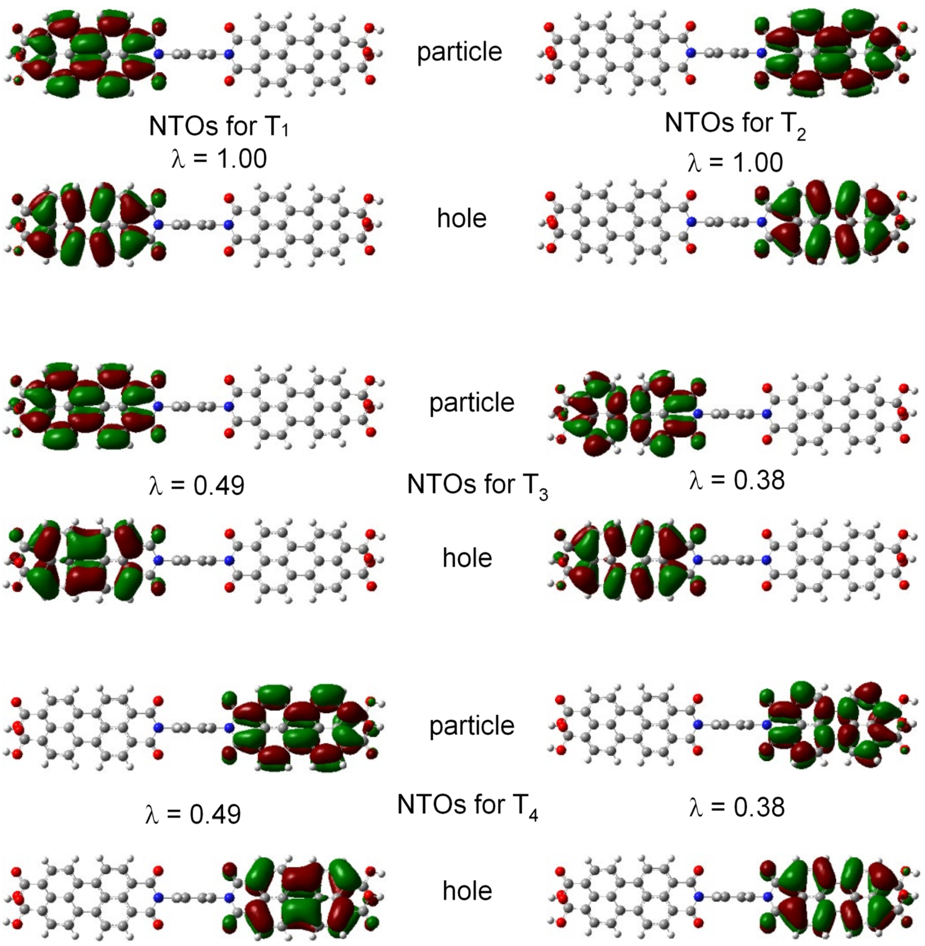

Figure S28. Major natural transition orbitals (NTOs) for triplet states of **PMIDE-*p*-d**. where λ refers to the occupation number for the relevant transition. Isovalue = 0.02.

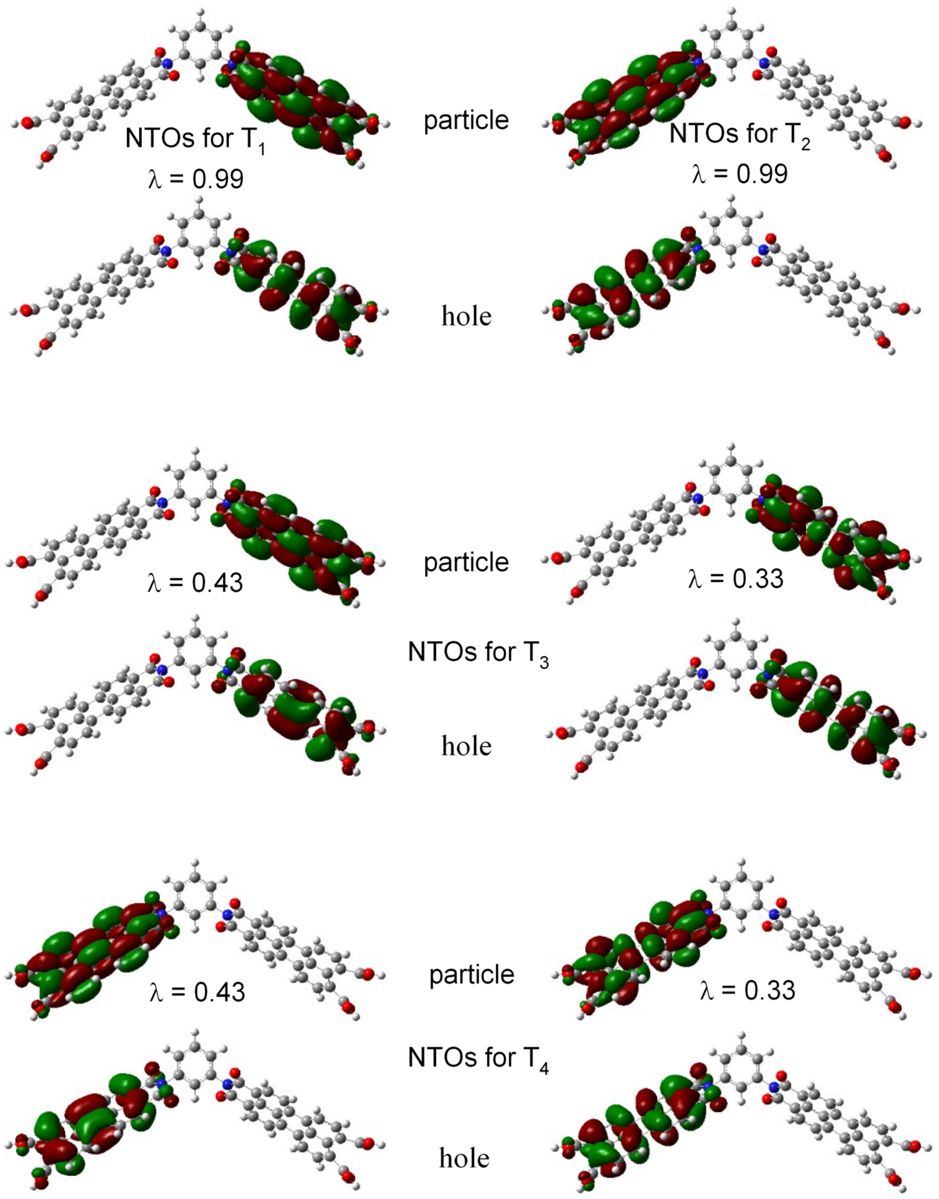

Figure S29. Major natural transition orbitals (NTOs) for triplet states of **PMIDE-*m*-d**, where λ refers to the occupation number for the relevant transition. Isovalue = 0.02.

Geometry optimizations of parallel-stacked **PMIDE-*m*-d** and **PMIDE-*p*-d** dimers were performed using the B3LYP-D3/6-31G(d) level of theory with the Gaussian 16 software package.[S13] TD-DFT calculations were performed on the dimer structures as well as on each fragment in the dimer geometry with the B3LYP hybrid functional and the 6-311G(d,p) basis set. The interaction energy between fragments (A and B) in these dimers were calculated using the Counterpoise method[S15] for which the basis set superposition error is taken into account in order to obtain corrected interaction energies using the formula below:

$$\Delta E_{int}^{CP} = E_{AB}^{AB} - E_{A}^{AB} - E_{B}^{AB} \quad \text{(S8)}$$

Here, superscript *AB* means that the whole basis set is utilized by using ghost atoms with no nuclei and electrons but empty basis set functions centered on them.

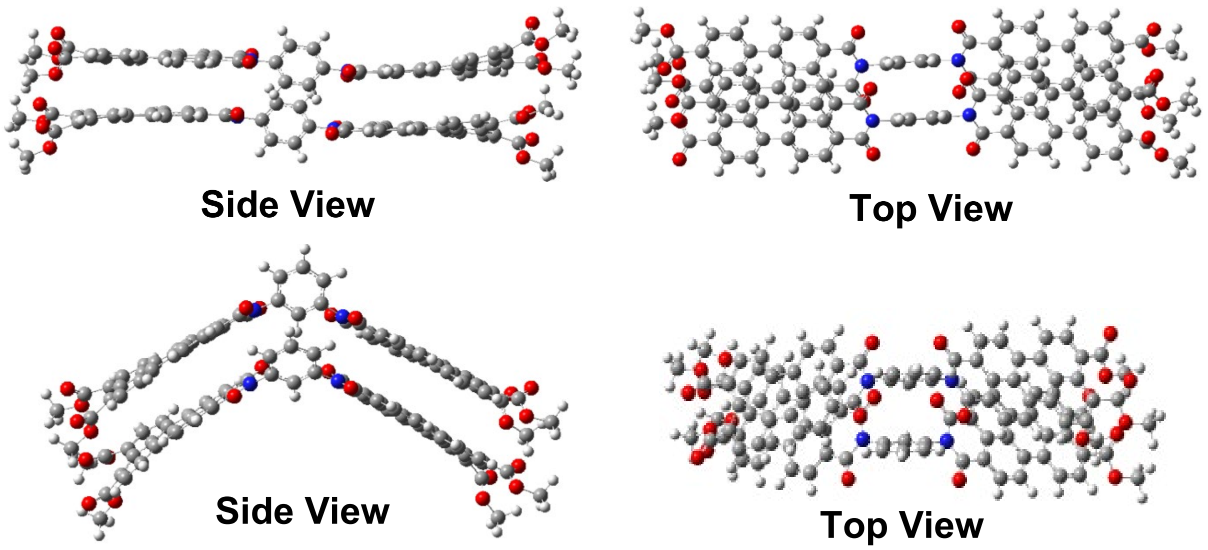

Figure S30. Optimized geometries of **PMIDE-*p*-d** (upper panel) and **PMIDE-*m*-d** (lower panel) with the B3LYP-D3/6-31G(d) method. Note that solubilizing chains are truncated to methoxy groups to save computational time in calculations.

The optimized geometries of dimers are given in Figure S30. The average distance between fragments has been found as 3.6 Å for **PMIDE-*m*-d**, slightly larger than that of **PMIDE-*p*-d** (3.5 Å). Since both dimer geometries display similar separation distances between the fragments, the interaction energy of **PMIDE-*p*-d** is only slightly larger than that of **PMIDE-*m*-d** (by 0.14 kcal/mole). Calculations hint that **PMIDE-*p*-d** might have slightly more tendency to form dimeric species than **PMIDE-*m*-d** under identical conditions, most probably due to favorable topology for effective dispersion interactions between the atoms on different fragments. Nonetheless, these results should be interpreted with caution since solvent effects are not included in the geometry optimizations.

Calculated vertical transition energies on dimer geometries show that the main singlet transition occurs at around 2.50 eV (see Table S2), very similar to those energies calculated

for optimized geometries of the corresponding bichromophores. TD-DFT calculations conducted on the individual fragments for the dimeric structures reveal that the singlet transition energies are not significantly different than those of the bichromophore vertical transition energies. However, TD-DFT calculations show the presence of a low-lying excited state at ~2.20 eV for both dimers with appreciable oscillator strength. Such transitions, which are absent in individual bichromophores, correspond to excitation to the J-state of the dimer. The experiment excitation energy is 2.27 eV in THF solution. The TD-DFT calculations indicate the presence of a further transition at higher energy that should correspond to excitation to the H-state. The computed excitation energy is 2.5 eV compared to the experimental value of 2.67 eV in THF solution.

Table S2. Calculated parameters for dimeric species of PMIDE bichromophores and their fragments held in dimer geometries.

	**PMIDE-*m*-d**	**PMIDE-*p*-d**
Average Distance between PMIs in Dimers	3.6 Å	3.5 Å
Interaction Energy (ΔE_{int}^{CP})	32.10 kcal/mole	32.24 kcal/mole
Singlet transition energies with significant f (dimer)	2.19 eV (f = 0.13)<br>2.21 eV (f = 0.09)<br>2.49 eV (f = 0.14)<br>2.52 eV (f = 1.14)<br>2.54 eV (f = 0.14)	2.18 eV (f = 0.11)<br>2.50 eV (f = 1.28)<br>2.50 eV (f = 0.84)
Singlet transition energies with significant f (fragment A only)	2.42 eV (f = 0.41)<br>2.42 eV (f = 0.55)<br>2.49 eV (f = 0.48)	2.42 eV (f = 0.08)<br>2.43 eV (f = 1.46)
Singlet transition energies with significant f (fragment B only)	2.44 eV (f = 1.15)<br>2.51 eV (f = 0.32)	2.41 eV (f = 0.13)<br>2.41 eV (f = 1.38)

Table S3. Cartesian coordinates for the dimer formed by association of two **PMIDE-*m*-d** molecules *in vacuo*.

6	-4.024704000	0.240722000	1.117333000
6	-5.093540000	1.092420000	1.418661000
6	-6.066450000	1.416233000	0.472727000
6	-5.928649000	0.900376000	-0.851598000
6	-4.850125000	0.013816000	-1.147105000
6	-3.904511000	-0.310616000	-0.145692000
6	-7.224098000	2.264273000	0.790479000
6	-6.845003000	1.260320000	-1.884844000
6	-7.870248000	2.277702000	-1.607691000
6	-8.070175000	2.740516000	-0.264718000
6	-8.645036000	2.823488000	-2.623933000
6	-6.709600000	0.648598000	-3.132692000
6	-5.670832000	-0.248349000	-3.405894000
6	-4.727919000	-0.551563000	-2.437408000
1	-5.573870000	-0.704588000	-4.385466000
1	-3.286125000	-0.015316000	1.868694000
6	-7.517195000	2.629243000	2.097658000
6	-8.624393000	3.426512000	2.396277000
6	-9.429224000	3.948450000	1.397881000
6	-9.112078000	3.690976000	0.023841000
1	-8.875431000	3.622220000	3.432023000
6	-9.547517000	3.857960000	-2.365358000
1	-10.079845000	4.332176000	-3.183096000
6	-9.764117000	4.323462000	-1.081554000
1	-7.420541000	0.858479000	-3.922857000
1	-8.515047000	2.502953000	-3.650503000
1	-5.149079000	1.500075000	2.421037000
1	-6.922262000	2.251962000	2.920342000
6	-3.594487000	-1.445083000	-2.779541000
6	-2.779896000	-1.228794000	-0.429077000
7	-2.633849000	-1.656926000	-1.766804000
6	-1.445074000	-2.417193000	-2.075816000
6	-1.522716000	-3.733848000	-2.520565000
6	-0.209983000	-1.808952000	-1.870827000
6	-0.347882000	-4.460866000	-2.702664000
6	0.955450000	-2.547144000	-2.057946000
6	0.896288000	-3.881154000	-2.462329000
1	-2.485172000	-4.190895000	-2.698356000
1	-0.160769000	-0.784659000	-1.523618000
1	-0.407963000	-5.500090000	-3.007317000
8	-3.477367000	-1.961241000	-3.879645000
8	-2.001904000	-1.592716000	0.440749000
6	-10.613458000	5.549212000	-0.998961000
8	-11.557539000	5.778237000	-1.726034000

8	-10.140878000	6.434403000	-0.090629000
6	-10.694001000	4.613266000	1.824368000
8	-11.764901000	4.546570000	1.248502000
8	-10.545133000	5.267571000	2.997288000
6	5.367623000	-1.088548000	-3.671436000
6	6.519738000	-0.309059000	-3.527942000
6	6.734707000	0.493057000	-2.404278000
6	5.763510000	0.470099000	-1.358879000
6	4.593996000	-0.335504000	-1.508639000
6	4.400637000	-1.099908000	-2.681505000
6	7.910842000	1.367094000	-2.273408000
6	5.942048000	1.231593000	-0.164197000
6	7.147722000	2.060444000	-0.013781000
6	8.090966000	2.159171000	-1.091112000
6	7.400276000	2.765858000	1.157089000
6	4.948154000	1.168733000	0.815105000
6	3.811925000	0.366172000	0.668723000
6	3.628336000	-0.387251000	-0.477717000
1	3.068052000	0.308270000	1.455219000
1	5.207062000	-1.686296000	-4.562329000
6	8.864555000	1.445589000	-3.280630000
6	10.008209000	2.232754000	-3.140201000
6	10.218402000	3.010394000	-2.013931000
6	9.223656000	3.045779000	-0.981683000
1	10.761827000	2.216640000	-3.918389000
6	8.441614000	3.693021000	1.229099000
1	8.568439000	4.304745000	2.115824000
6	9.292338000	3.905081000	0.160205000
1	5.041969000	1.745509000	1.726696000
1	6.765429000	2.642196000	2.025361000
1	7.243692000	-0.331335000	-4.333449000
1	8.757503000	0.869138000	-4.191083000
6	2.432453000	-1.252660000	-0.594542000
6	3.166492000	-1.893307000	-2.891365000
7	2.223444000	-1.888664000	-1.838156000
8	1.650253000	-1.402634000	0.333502000
8	2.963585000	-2.510359000	-3.924324000
6	10.125793000	5.139141000	0.266458000
8	10.579038000	5.583664000	1.300199000
8	10.198760000	5.794129000	-0.917673000
6	11.558406000	3.651598000	-1.878466000
8	12.169435000	3.820544000	-0.840750000
8	12.079298000	3.982844000	-3.082124000
1	1.806464000	-4.452964000	-2.573723000
6	-4.670273000	-2.622537000	2.969242000
6	-5.900187000	-1.966320000	2.845575000
6	-6.656688000	-2.025862000	1.673541000

6	-6.154421000	-2.799122000	0.584554000
6	-4.928166000	-3.515056000	0.739969000
6	-4.176986000	-3.391595000	1.930634000
6	-7.934619000	-1.313131000	1.518033000
6	-6.848140000	-2.864025000	-0.661507000
6	-8.052992000	-2.044159000	-0.853534000
6	-8.597906000	-1.297654000	0.243909000
6	-8.684048000	-1.972089000	-2.088803000
6	-6.342629000	-3.696632000	-1.661675000
6	-5.175113000	-4.445766000	-1.479422000
6	-4.454880000	-4.347431000	-0.301956000
1	-4.812214000	-5.103284000	-2.261882000
1	-4.078704000	-2.534212000	3.874438000
6	-8.515600000	-0.632668000	2.579239000
6	-9.746007000	0.015557000	2.441077000
6	-10.413074000	0.048141000	1.230909000
6	-9.812163000	-0.538287000	0.068959000
1	-10.207000000	0.476695000	3.307130000
6	-9.800677000	-1.159510000	-2.284684000
1	-10.247432000	-1.076297000	-3.269483000
6	-10.354253000	-0.423380000	-1.252408000
1	-6.853962000	-3.786698000	-2.611899000
1	-8.290686000	-2.510150000	-2.942088000
1	-6.240652000	-1.373903000	3.686497000
1	-8.046328000	-0.628720000	3.555640000
6	-3.204549000	-5.126637000	-0.147630000
6	-2.851102000	-4.043550000	2.072374000
7	-2.422130000	-4.835623000	0.982298000
6	-1.109364000	-5.431059000	1.060925000
6	-0.990783000	-6.816157000	1.158809000
6	0.014655000	-4.606635000	1.018438000
6	0.275382000	-7.394125000	1.218092000
6	1.271211000	-5.202887000	1.107449000
6	1.410278000	-6.587150000	1.203113000
1	-1.884994000	-7.428867000	1.173022000
1	-0.087023000	-3.530792000	0.927668000
1	0.377230000	-8.473153000	1.283847000
8	-2.852945000	-5.958433000	-0.972506000
8	-2.165744000	-3.922148000	3.071289000
6	-11.448176000	0.503025000	-1.690560000
8	-12.190266000	0.265424000	-2.621206000
8	-11.446779000	1.670239000	-1.019984000
6	-11.836481000	0.502969000	1.265670000
8	-12.758092000	-0.078918000	0.730622000
8	-11.996376000	1.585317000	2.057759000
6	5.554630000	-3.770874000	-0.844434000
6	6.736377000	-3.024312000	-0.778278000

6	7.023730000	-2.192848000	0.306273000
6	6.087526000	-2.133721000	1.381734000
6	4.870343000	-2.875487000	1.295704000
6	4.619607000	-3.701517000	0.174670000
6	8.244461000	-1.373140000	0.372899000
6	6.338757000	-1.332721000	2.534875000
6	7.627705000	-0.635803000	2.657706000
6	8.559034000	-0.640511000	1.567065000
6	7.974169000	0.029047000	3.827072000
6	5.343558000	-1.244198000	3.510746000
6	4.141221000	-1.951915000	3.406716000
6	3.902430000	-2.775476000	2.320833000
1	3.379496000	-1.874815000	4.175356000
1	5.349345000	-4.411529000	-1.695466000
6	9.111901000	-1.288644000	-0.707161000
6	10.329616000	-0.612339000	-0.607427000
6	10.714976000	0.016638000	0.563299000
6	9.803864000	0.087686000	1.669056000
1	11.009744000	-0.615287000	-1.450779000
6	9.165092000	0.748319000	3.923946000
1	9.405543000	1.278656000	4.838904000
6	10.054806000	0.831557000	2.867051000
1	5.482688000	-0.610529000	4.378540000
1	7.320299000	0.006597000	4.690554000
1	7.433278000	-3.110005000	-1.602689000
1	8.869297000	-1.766958000	-1.647784000
6	2.637223000	-3.545151000	2.262569000
6	3.384089000	-4.517785000	0.086227000
7	2.460254000	-4.382746000	1.134151000
8	1.802214000	-3.496476000	3.146843000
8	3.182179000	-5.282660000	-0.848255000
6	11.190793000	1.777470000	3.130095000
8	11.722896000	1.877318000	4.216180000
8	11.461129000	2.580568000	2.086802000
6	12.152424000	0.413493000	0.657330000
8	12.859508000	0.230678000	1.627367000
8	12.609483000	0.904524000	-0.515295000
1	2.402618000	-7.021505000	1.258218000
6	-13.357558000	2.019636000	2.225174000
1	-13.304264000	2.895180000	2.870300000
1	-13.789044000	2.294352000	1.261230000
1	-13.958207000	1.227271000	2.680263000
6	-12.533958000	2.561916000	-1.351806000
1	-13.482435000	2.029108000	-1.249139000
1	-12.453652000	3.384099000	-0.646747000
1	-12.425430000	2.923590000	-2.376191000
6	-11.743059000	5.848732000	3.536201000

1	-11.435006000	6.351535000	4.453273000
1	-12.174927000	6.563162000	2.830492000
1	-12.484841000	5.074969000	3.754167000
6	-10.955931000	7.604252000	0.088849000
1	-11.965516000	7.310425000	0.390063000
1	-10.466323000	8.181878000	0.873680000
1	-11.012832000	8.180263000	-0.838468000
6	12.576712000	3.472408000	2.302201000
1	13.466489000	2.881866000	2.536421000
1	12.685386000	4.018387000	1.371921000
6	13.999221000	1.267081000	-0.527091000
1	14.224682000	1.513622000	-1.565474000
1	14.160336000	2.138986000	0.110177000
1	14.619841000	0.436559000	-0.181436000
6	13.407144000	4.529077000	-3.044562000
1	13.669091000	4.729183000	-4.083788000
1	13.424868000	5.452102000	-2.458534000
1	14.107651000	3.816521000	-2.600122000
6	11.029559000	6.965392000	-0.906283000
1	10.996091000	7.356213000	-1.923936000
1	10.648565000	7.702841000	-0.194983000
1	12.051564000	6.693306000	-0.626321000
1	12.356539000	4.152118000	3.127180000

Table S4. Cartesian coordinates for the dimer formed by association of two **PMIDE-*p*-d** molecules *in vacuo*.

6	-5.209999000	0.640627000	-2.504635000
6	-6.608422000	0.626818000	-2.511795000
6	-7.357660000	1.322872000	-1.560689000
6	-6.662749000	2.088308000	-0.576967000
6	-5.235207000	2.079221000	-0.560574000
6	-4.518422000	1.352133000	-1.538181000
6	-8.828183000	1.286404000	-1.533117000
6	-7.368715000	2.851490000	0.399665000
6	-8.836857000	2.911780000	0.339367000
6	-9.554957000	2.113843000	-0.612017000
6	-9.555781000	3.742395000	1.189642000
6	-6.630294000	3.519386000	1.379661000
6	-5.232110000	3.487834000	1.402248000
6	-4.528869000	2.785909000	0.438457000
1	-4.675348000	4.012845000	2.171154000
1	-4.645176000	0.088870000	-3.248276000
6	-9.540682000	0.456397000	-2.387331000
6	-10.934711000	0.504523000	-2.449186000
6	-11.665336000	1.362965000	-1.646906000

6	-10.999953000	2.140835000	-0.641839000
1	-11.456241000	-0.112392000	-3.171056000
6	-10.950366000	3.752205000	1.182704000
1	-11.491969000	4.389679000	1.872999000
6	-11.680711000	2.944445000	0.328639000
1	-7.133996000	4.078522000	2.158979000
1	-9.045590000	4.396992000	1.885880000
1	-7.102128000	0.051991000	-3.285159000
1	-9.025170000	-0.232949000	-3.044040000
6	-3.048078000	2.783081000	0.483285000
6	-3.037539000	1.339062000	-1.552130000
7	-2.382097000	2.018159000	-0.507489000
6	-0.942511000	1.920922000	-0.418895000
6	-0.146762000	2.272893000	-1.509711000
6	-0.364247000	1.449718000	0.759650000
6	1.234584000	2.128784000	-1.425221000
6	1.018945000	1.325172000	0.849517000
6	1.811607000	1.648073000	-0.249387000
1	-0.604999000	2.630795000	-2.423415000
1	-0.996185000	1.152941000	1.586658000
1	1.862795000	2.375274000	-2.274637000
1	1.472261000	0.948080000	1.755328000
8	-2.424306000	3.411940000	1.320167000
8	-2.414500000	0.755145000	-2.429686000
6	-13.158177000	2.994358000	0.592241000
8	-13.735088000	4.015876000	0.902507000
8	-13.736926000	1.781701000	0.575663000
6	-13.097069000	1.563918000	-2.024447000
8	-13.659958000	2.639259000	-2.059508000
8	-13.668635000	0.410468000	-2.433464000
6	6.219606000	2.967197000	1.506421000
6	7.613267000	3.001638000	1.381216000
6	8.284411000	2.277492000	0.392294000
6	7.517855000	1.420319000	-0.453589000
6	6.094836000	1.407209000	-0.334773000
6	5.453879000	2.202927000	0.642253000
6	9.733158000	2.400824000	0.161393000
6	8.148164000	0.579791000	-1.419921000
6	9.613533000	0.594204000	-1.537111000
6	10.378251000	1.567590000	-0.814393000
6	10.284301000	-0.300958000	-2.360922000
6	7.340582000	-0.209168000	-2.241756000
6	5.946328000	-0.204806000	-2.132310000
6	5.318583000	0.590866000	-1.189275000
1	5.336887000	-0.829626000	-2.775345000
1	5.714530000	3.558422000	2.263082000
6	10.499077000	3.328560000	0.855750000

6	11.842429000	3.546541000	0.530636000
6	12.473292000	2.806771000	-0.452695000
6	11.791582000	1.705495000	-1.067353000
1	12.384277000	4.354239000	1.010843000
6	11.668996000	-0.241773000	-2.520617000
1	12.176882000	-0.980138000	-3.130065000
6	12.432345000	0.738211000	-1.907981000
1	7.785970000	-0.841526000	-2.999711000
1	9.746191000	-1.091675000	-2.868677000
1	8.165221000	3.634793000	2.065973000
1	10.051161000	3.961124000	1.612579000
6	3.840902000	0.579643000	-1.099101000
6	3.975107000	2.258799000	0.740294000
7	3.250387000	1.489599000	-0.200279000
8	3.159099000	-0.170405000	-1.785679000
8	3.402496000	2.946468000	1.568965000
6	13.905957000	0.573535000	-2.135173000
8	14.370797000	0.001638000	-3.098662000
8	14.667366000	1.034586000	-1.122883000
6	13.745753000	3.367455000	-0.999461000
8	13.964012000	3.535974000	-2.179701000
8	14.584950000	3.779682000	-0.017509000
6	-6.158255000	-2.969993000	-1.641709000
6	-7.555843000	-2.933401000	-1.597647000
6	-8.246672000	-2.220111000	-0.614653000
6	-7.490892000	-1.467366000	0.333550000
6	-6.064053000	-1.505539000	0.279094000
6	-5.408570000	-2.278319000	-0.706610000
6	-9.714418000	-2.238701000	-0.511338000
6	-8.133815000	-0.675479000	1.332993000
6	-9.603110000	-0.655375000	1.399279000
6	-10.373587000	-1.479749000	0.512165000
6	-10.276282000	0.141004000	2.318509000
6	-7.333258000	0.038736000	2.227250000
6	-5.936442000	0.004985000	2.162536000
6	-5.296384000	-0.760084000	1.202924000
1	-5.334707000	0.581633000	2.855893000
1	-5.639615000	-3.550039000	-2.397677000
6	-10.488511000	-2.988340000	-1.388424000
6	-11.882406000	-2.975052000	-1.322680000
6	-12.555082000	-2.257301000	-0.347915000
6	-11.808887000	-1.539376000	0.644173000
1	-12.453409000	-3.519938000	-2.064876000
6	-11.653336000	0.013246000	2.510311000
1	-12.149366000	0.570099000	3.298019000
6	-12.406457000	-0.868685000	1.757885000
1	-7.785674000	0.642779000	3.003708000

1	-9.737596000	0.844975000	2.940115000
1	-8.096043000	-3.499254000	-2.347021000
1	-10.025695000	-3.580881000	-2.167830000
6	-3.815514000	-0.779059000	1.157877000
6	-3.929959000	-2.390501000	-0.741985000
7	-3.217420000	-1.666158000	0.240754000
6	-1.782433000	-1.845969000	0.309533000
6	-0.973845000	-1.516496000	-0.776097000
6	-1.226102000	-2.351535000	1.484536000
6	0.406380000	-1.656137000	-0.671245000
6	0.153141000	-2.506170000	1.585239000
6	0.964631000	-2.143249000	0.510274000
1	-1.413360000	-1.118410000	-1.679925000
1	-1.867191000	-2.603766000	2.322405000
1	1.052235000	-1.351843000	-1.484524000
1	0.597791000	-2.876401000	2.500762000
8	-3.139770000	-0.071102000	1.892787000
8	-3.351058000	-3.084378000	-1.560748000
6	-13.770954000	-1.138300000	2.300409000
8	-14.465173000	-0.323160000	2.870672000
8	-14.090168000	-2.452420000	2.212410000
6	-14.036913000	-2.154957000	-0.486412000
8	-14.718556000	-1.191445000	-0.193794000
8	-14.564671000	-3.264445000	-1.052716000
6	5.152454000	-0.657799000	2.590202000
6	6.547852000	-0.567173000	2.591705000
6	7.330658000	-1.261543000	1.667907000
6	6.681004000	-2.105255000	0.718157000
6	5.255160000	-2.162378000	0.696637000
6	4.502353000	-1.438272000	1.649286000
6	8.795289000	-1.149419000	1.639788000
6	7.427534000	-2.883596000	-0.216389000
6	8.896610000	-2.883985000	-0.135370000
6	9.563778000	-2.010630000	0.788361000
6	9.667792000	-3.713668000	-0.940595000
6	6.724980000	-3.629281000	-1.165824000
6	5.326114000	-3.647046000	-1.206791000
6	4.585697000	-2.933038000	-0.280401000
1	4.798488000	-4.224565000	-1.958458000
1	4.558019000	-0.111149000	3.314220000
6	9.460318000	-0.212237000	2.419180000
6	10.848276000	-0.081582000	2.367315000
6	11.629711000	-0.934137000	1.604343000
6	11.002919000	-1.986052000	0.853297000
1	11.323935000	0.729168000	2.903694000
6	11.053487000	-3.793154000	-0.776465000
1	11.629057000	-4.521301000	-1.338514000

6	11.719486000	-2.998923000	0.138164000
1	7.257713000	-4.214095000	-1.906278000
1	9.201907000	-4.373619000	-1.662512000
1	7.012191000	0.063588000	3.339333000
1	8.905105000	0.489037000	3.029051000
6	3.104692000	-2.977737000	-0.343989000
6	3.022930000	-1.506122000	1.668258000
7	2.403518000	-2.219807000	0.626052000
8	2.511020000	-3.638322000	-1.178012000
8	2.372316000	-0.952981000	2.545059000
6	13.137044000	-3.405563000	0.392783000
8	13.887231000	-3.844011000	-0.451646000
8	13.426639000	-3.378254000	1.715823000
6	13.069892000	-0.584077000	1.437183000
8	13.781419000	-0.951478000	0.521372000
8	13.497856000	0.262257000	2.399302000
6	15.774803000	4.440293000	-0.483590000
1	16.330950000	4.708740000	0.415249000
1	16.360997000	3.767205000	-1.115068000
1	15.519081000	5.332429000	-1.061575000
6	16.067231000	0.747402000	-1.237605000
1	16.528099000	1.201857000	-0.359864000
1	16.223902000	-0.333998000	-1.237799000
1	16.472767000	1.177666000	-2.156968000
6	14.800552000	0.838582000	2.200560000
1	15.053767000	1.314358000	3.148865000
1	15.529919000	0.064505000	1.949580000
1	14.758262000	1.579354000	1.398373000
6	14.792444000	-3.686642000	2.032649000
1	14.863908000	-3.617576000	3.118879000
1	15.052437000	-4.691992000	1.690675000
1	15.455957000	-2.961290000	1.552518000
6	-15.410580000	-2.782285000	2.670280000
1	-16.154896000	-2.234925000	2.084277000
1	-15.511200000	-3.857699000	2.519172000
1	-15.528855000	-2.526394000	3.726425000
6	-15.978916000	-3.212829000	-1.296846000
1	-16.226770000	-2.387685000	-1.970383000
1	-16.230858000	-4.170633000	-1.752711000
1	-16.524737000	-3.077529000	-0.358991000
6	-15.165486000	1.805653000	0.785916000
1	-15.629460000	2.431118000	0.018515000
1	-15.479166000	0.770535000	0.710204000
1	-15.389857000	2.206987000	1.775646000
6	-15.038153000	0.519177000	-2.851693000
1	-15.154731000	1.307999000	-3.598986000
1	-15.289994000	-0.454659000	-3.273702000

1	-15.672876000	0.731184000	-1.988683000

Table S5. Cartesian coordinates for fragment 1 of the dimer formed by association of two **PMIDE-*m*-d** molecules *in vacuo*.

6	-4.098792000	0.914033000	-2.172721000
6	-5.220130000	0.079748000	-2.100831000
6	-6.127570000	0.155133000	-1.043916000
6	-5.866373000	1.076797000	0.014973000
6	-4.734493000	1.941268000	-0.074827000
6	-3.858576000	1.849514000	-1.182356000
6	-7.338527000	-0.675646000	-0.983288000
6	-6.712017000	1.144053000	1.162677000
6	-7.794443000	0.159486000	1.310966000
6	-8.116940000	-0.714701000	0.220308000
6	-8.507588000	0.037490000	2.497333000
6	-6.455505000	2.130204000	2.117335000
6	-5.364577000	2.999432000	2.005485000
6	-4.489586000	2.898994000	0.936237000
1	-5.173434000	3.751637000	2.763621000
1	-3.412130000	0.855212000	-3.009760000
6	-7.747241000	-1.439987000	-2.067983000
6	-8.904476000	-2.219804000	-2.009736000
6	-9.648028000	-2.323506000	-0.846403000
6	-9.214266000	-1.639179000	0.336140000
1	-9.243435000	-2.736418000	-2.899853000
6	-9.466924000	-0.964617000	2.661413000
1	-9.951355000	-1.101446000	3.622430000
6	-9.801488000	-1.820368000	1.628205000
1	-7.109803000	2.243709000	2.973406000
1	-8.285105000	0.675927000	3.343729000
1	-5.369920000	-0.637093000	-2.899410000
1	-7.204584000	-1.399843000	-3.004469000
6	-3.299079000	3.782235000	0.884488000
6	-2.680510000	2.736709000	-1.297087000
7	-2.413440000	3.579940000	-0.196280000
6	-1.175556000	4.323348000	-0.237142000
6	-1.167051000	5.714991000	-0.262971000
6	0.015173000	3.605063000	-0.297618000
6	0.045695000	6.385330000	-0.410770000
6	1.219380000	4.288182000	-0.443937000
6	1.243797000	5.681382000	-0.514672000
1	-2.094176000	6.264889000	-0.193145000
1	-0.002684000	2.522729000	-0.277986000
1	0.049963000	7.468056000	-0.474755000
8	-3.076075000	4.630030000	1.734123000

8	-1.959713000	2.736902000	-2.284386000
6	-10.701471000	-2.946320000	2.019266000
8	-11.593708000	-2.857343000	2.836807000
8	-10.336258000	-4.113464000	1.439305000
6	-10.967151000	-3.011976000	-0.943633000
8	-11.986152000	-2.688044000	-0.360879000
8	-10.936249000	-4.030832000	-1.830761000
6	5.684869000	3.182712000	1.298322000
6	6.791144000	2.329450000	1.358847000
6	6.886043000	1.184128000	0.564207000
6	5.837390000	0.915090000	-0.365607000
6	4.715285000	1.796092000	-0.428078000
6	4.644316000	2.921179000	0.424248000
6	8.013580000	0.244767000	0.667282000
6	5.891993000	-0.213669000	-1.238495000
6	7.049169000	-1.119013000	-1.170927000
6	8.069553000	-0.908659000	-0.183676000
6	7.181544000	-2.191732000	-2.045044000
6	4.827462000	-0.421543000	-2.118479000
6	3.738132000	0.453367000	-2.185107000
6	3.674397000	1.558628000	-1.354437000
1	2.937607000	0.289966000	-2.897664000
1	5.617989000	4.054257000	1.940746000
6	9.039671000	0.449878000	1.581226000
6	10.137461000	-0.409031000	1.647752000
6	10.228345000	-1.531933000	0.842631000
6	9.155008000	-1.849878000	-0.053565000
1	10.949905000	-0.179497000	2.326741000
6	8.177366000	-3.152707000	-1.860474000
1	8.210426000	-4.033856000	-2.492183000
6	9.100539000	-3.045847000	-0.836966000
1	4.827039000	-1.276143000	-2.783406000
1	6.485751000	-2.327737000	-2.863331000
1	7.576420000	2.575837000	2.063178000
1	9.026807000	1.304624000	2.245999000
6	2.525752000	2.486399000	-1.466557000
6	3.462074000	3.815011000	0.426252000
7	2.439781000	3.515639000	-0.503212000
8	1.679496000	2.364286000	-2.340934000
8	3.364911000	4.755170000	1.197996000
6	9.874716000	-4.293597000	-0.567502000
8	10.227869000	-5.087690000	-1.413877000
8	10.015028000	-4.515112000	0.762063000
6	11.527768000	-2.264403000	0.852236000
8	12.048448000	-2.810853000	-0.101199000
8	12.128343000	-2.203393000	2.062786000
1	2.181345000	6.198774000	-0.659945000

6	-12.194368000	-4.682748000	-2.066010000
1	-11.978945000	-5.483189000	-2.774258000
1	-12.596812000	-5.089304000	-1.134507000
1	-12.920484000	-3.982360000	-2.488705000
6	-11.207528000	-5.221658000	1.718350000
1	-12.225520000	-4.983342000	1.397066000
1	-10.803758000	-6.059337000	1.148621000
1	-11.213691000	-5.447210000	2.787944000
6	13.427015000	-2.812919000	2.132694000
1	13.761803000	-2.667713000	3.160060000
1	13.362957000	-3.878489000	1.895683000
1	14.117506000	-2.337127000	1.430685000
6	10.796436000	-5.671769000	1.099345000
1	10.827872000	-5.694399000	2.189289000
1	10.332535000	-6.580013000	0.705870000
1	11.803066000	-5.574734000	0.681990000

Table S6. Cartesian coordinates for fragment 2 of the dimer formed by association of two **PMIDE-*m*-d** molecules *in vacuo*.

6	-4.569458000	0.494035000	-2.420913000
6	-5.784477000	-0.187660000	-2.288159000
6	-6.617513000	-0.007702000	-1.182189000
6	-6.211144000	0.917241000	-0.174499000
6	-4.999497000	1.653915000	-0.346666000
6	-4.168621000	1.408022000	-1.463080000
6	-7.880804000	-0.743172000	-1.013498000
6	-6.986567000	1.114393000	1.007862000
6	-8.176128000	0.280225000	1.231092000
6	-8.625287000	-0.620921000	0.208950000
6	-8.884214000	0.342655000	2.424420000
6	-6.572939000	2.086911000	1.920130000
6	-5.419124000	2.850799000	1.713499000
6	-4.620736000	2.630309000	0.604990000
1	-5.128208000	3.616468000	2.424406000
1	-3.917521000	0.313368000	-3.269131000
6	-8.370300000	-1.575614000	-2.010574000
6	-9.587000000	-2.247553000	-1.864545000
6	-10.330552000	-2.150584000	-0.703393000
6	-9.824935000	-1.397678000	0.406834000
1	-9.976207000	-2.833062000	-2.689717000
6	-9.986253000	-0.480414000	2.655898000
1	-10.493789000	-0.454007000	3.614049000
6	-10.448630000	-1.363271000	1.696368000
1	-7.147848000	2.278142000	2.817576000
1	-8.563947000	1.000169000	3.222820000

1	-6.051155000	-0.895432000	-3.064024000
1	-7.838226000	-1.686463000	-2.947698000
6	-3.387056000	3.430751000	0.427135000
6	-2.856570000	2.086862000	-1.607311000
7	-2.523944000	3.027777000	-0.605462000
6	-1.227403000	3.658177000	-0.680680000
6	-1.144498000	5.022885000	-0.950566000
6	-0.084223000	2.889700000	-0.462780000
6	0.104825000	5.636661000	-1.007211000
6	1.156909000	3.517424000	-0.551810000
6	1.260127000	4.882374000	-0.818141000
1	-2.053932000	5.593980000	-1.098916000
1	-0.159131000	1.831213000	-0.239149000
1	0.178169000	6.701512000	-1.206492000
8	-3.115438000	4.374130000	1.156648000
8	-2.103957000	1.865021000	-2.538248000
6	-11.540445000	-2.267274000	2.183799000
8	-12.348679000	-1.941188000	3.028594000
8	-11.459962000	-3.509779000	1.672168000
6	-11.734266000	-2.660390000	-0.765433000
8	-12.706047000	-2.050610000	-0.367884000
8	-11.809263000	-3.840526000	-1.418199000
6	5.344636000	2.512330000	1.827459000
6	6.550156000	1.809330000	1.931233000
6	6.932819000	0.857564000	0.983674000
6	6.071217000	0.625437000	-0.130036000
6	4.829197000	1.324868000	-0.215680000
6	4.480833000	2.277322000	0.770924000
6	8.179458000	0.083509000	1.098880000
6	6.421422000	-0.306300000	-1.151640000
6	7.736077000	-0.963241000	-1.103874000
6	8.593463000	-0.783289000	0.031643000
6	8.178223000	-1.758471000	-2.153443000
6	5.495348000	-0.557079000	-2.166568000
6	4.267984000	0.111481000	-2.229061000
6	3.933909000	1.057448000	-1.276194000
1	3.560870000	-0.092504000	-3.026154000
1	5.064866000	3.248265000	2.573760000
6	8.976505000	0.171251000	2.231638000
6	10.217978000	-0.465039000	2.295233000
6	10.697926000	-1.223400000	1.242022000
6	9.863694000	-1.470255000	0.101412000
1	10.841054000	-0.328112000	3.170934000
6	9.394082000	-2.438004000	-2.083060000
1	9.709784000	-3.071440000	-2.904835000
6	10.214823000	-2.351125000	-0.972032000
1	5.710022000	-1.290773000	-2.934401000

1	7.581888000	-1.871796000	-3.050769000
1	7.188706000	2.026480000	2.778444000
1	8.658545000	0.756331000	3.085450000
6	2.644965000	1.779142000	-1.395877000
6	3.218121000	3.050192000	0.676854000
7	2.369343000	2.746832000	-0.398972000
8	1.871468000	1.585720000	-2.315572000
8	2.932509000	3.920041000	1.489779000
6	11.393600000	-3.278639000	-1.039951000
8	11.998412000	-3.496153000	-2.069307000
8	11.619174000	-3.930670000	0.113514000
6	12.149760000	-1.573585000	1.288299000
8	12.912978000	-1.489407000	0.347675000
8	12.543695000	-1.892633000	2.540531000
1	2.240394000	5.343854000	-0.868665000
6	-13.142850000	-4.344593000	-1.610389000
1	-13.021068000	-5.292795000	-2.131626000
1	-13.628017000	-4.510056000	-0.647023000
1	-13.736051000	-3.640605000	-2.200509000
6	-12.539139000	-4.392713000	2.050265000
1	-13.494500000	-3.914204000	1.821468000
1	-12.388102000	-5.294664000	1.464421000
1	-12.487028000	-4.615903000	3.117702000
6	12.772851000	-4.799282000	0.084464000
1	13.657833000	-4.209868000	-0.169898000
1	12.836825000	-5.217161000	1.082766000
6	13.939982000	-2.197117000	2.684162000
1	14.104334000	-2.299929000	3.757602000
1	14.168682000	-3.136570000	2.176806000
1	14.556563000	-1.394459000	2.271873000
1	12.627742000	-5.586909000	-0.656926000

Table S7. Cartesian coordinates for fragment 1 of the dimer formed by association of two **PMIDE-*p*-d** molecules *in vacuo*.

6	-5.585380000	2.254095000	0.327499000
6	-6.983441000	2.289367000	0.324605000
6	-7.750176000	1.128170000	0.205480000
6	-7.073915000	-0.125101000	0.113366000
6	-5.646655000	-0.152319000	0.093024000
6	-4.911780000	1.049820000	0.206925000
6	-9.220560000	1.153909000	0.162626000
6	-7.798270000	-1.350857000	0.026894000
6	-9.266028000	-1.324752000	0.114738000
6	-9.965592000	-0.073367000	0.161100000
6	-10.002372000	-2.501700000	0.163007000

6	-7.077623000	-2.536662000	-0.135214000
6	-5.679600000	-2.552992000	-0.176329000
6	-4.958652000	-1.377766000	-0.052980000
1	-5.136790000	-3.483697000	-0.302751000
1	-5.006809000	3.167357000	0.415085000
6	-9.915437000	2.354568000	0.120068000
6	-11.308967000	2.388881000	0.199789000
6	-12.056662000	1.229089000	0.300894000
6	-11.410294000	-0.047918000	0.203764000
1	-11.816337000	3.345720000	0.226406000
6	-11.396801000	-2.482473000	0.177491000
1	-11.952236000	-3.413907000	0.189238000
6	-12.109714000	-1.296564000	0.151381000
1	-7.595578000	-3.482255000	-0.241337000
1	-9.506349000	-3.464590000	0.181558000
1	-7.462722000	3.255820000	0.416916000
1	-9.386056000	3.296209000	0.047359000
6	-3.478549000	-1.431071000	-0.087824000
6	-3.430779000	1.047004000	0.203856000
7	-2.793988000	-0.193060000	0.006905000
6	-1.354812000	-0.214994000	-0.127273000
6	-0.548837000	0.347110000	0.863394000
6	-0.786978000	-0.782684000	-1.267749000
6	0.832557000	0.360486000	0.697276000
6	0.596028000	-0.786433000	-1.422950000
6	1.399264000	-0.198804000	-0.448438000
1	-0.998923000	0.792343000	1.742098000
1	-1.426410000	-1.188372000	-2.040994000
1	1.468966000	0.816551000	1.448210000
1	1.041454000	-1.212878000	-2.310602000
8	-2.870456000	-2.482796000	-0.182818000
8	-2.792088000	2.080351000	0.357182000
6	-13.590663000	-1.503791000	0.014530000
8	-14.180047000	-2.409900000	0.566059000
8	-14.158763000	-0.670517000	-0.873546000
6	-13.485344000	1.395902000	0.705643000
8	-14.056948000	0.709855000	1.528467000
8	-14.041973000	2.480702000	0.124436000
6	5.774026000	-2.451087000	-0.653593000
6	7.168766000	-2.401859000	-0.546691000
6	7.858119000	-1.192617000	-0.422941000
6	7.109322000	0.020859000	-0.491150000
6	5.685122000	-0.037482000	-0.577882000
6	5.025486000	-1.286290000	-0.640584000
6	9.308470000	-1.125300000	-0.179335000
6	7.758564000	1.291907000	-0.468859000
6	9.225078000	1.347541000	-0.382326000

6	9.972547000	0.147659000	-0.145322000
6	9.913501000	2.548801000	-0.496156000
6	6.967868000	2.442441000	-0.502464000
6	5.572395000	2.378924000	-0.570001000
6	4.926385000	1.155181000	-0.609987000
1	4.976228000	3.283843000	-0.601910000
1	5.254699000	-3.401339000	-0.720712000
6	10.057877000	-2.273565000	0.042779000
6	11.403185000	-2.198165000	0.419965000
6	12.052326000	-0.982009000	0.528443000
6	11.387631000	0.222111000	0.123947000
1	11.932191000	-3.103737000	0.696853000
6	11.299487000	2.607185000	-0.347760000
1	11.821084000	3.546698000	-0.488509000
6	12.046875000	1.484615000	-0.032102000
1	7.427881000	3.422094000	-0.464905000
1	9.388425000	3.463389000	-0.741988000
1	7.706872000	-3.342481000	-0.536442000
1	9.595348000	-3.252483000	0.006132000
6	3.447863000	1.117646000	-0.676029000
6	3.545202000	-1.374598000	-0.662085000
7	2.838641000	-0.150392000	-0.601620000
8	2.780946000	2.139852000	-0.771598000
8	2.956642000	-2.441913000	-0.705728000
6	13.524509000	1.741756000	-0.004835000
8	14.005966000	2.833179000	0.215278000
8	14.269517000	0.670213000	-0.342177000
6	13.326526000	-0.970621000	1.308675000
8	13.557739000	-0.210688000	2.224123000
8	14.150102000	-1.988088000	0.955271000
6	15.339862000	-2.102312000	1.755779000
1	15.882666000	-2.957572000	1.351840000
1	15.939469000	-1.191338000	1.678106000
1	15.083593000	-2.267174000	2.805786000
6	15.673087000	0.927035000	-0.481075000
1	16.119288000	-0.035767000	-0.732618000
1	15.839001000	1.649325000	-1.284033000
1	16.086127000	1.315201000	0.453559000
6	-15.589938000	-0.821476000	-0.994016000
1	-16.049839000	-0.664004000	-0.014691000
1	-15.893796000	-0.067172000	-1.711158000
1	-15.829813000	-1.821671000	-1.358771000
6	-15.407159000	2.738593000	0.487853000
1	-15.521347000	2.766557000	1.574341000
1	-15.645479000	3.707973000	0.048106000
1	-16.054171000	1.965371000	0.068199000

Table S8. Cartesian coordinates for fragment 2 of the dimer formed by association of two **PMIDE-*p*-d** molecules *in vacuo*.

6	-5.781868000	-2.431185000	-0.583665000
6	-7.178670000	-2.361908000	-0.557662000
6	-7.857075000	-1.146190000	-0.438911000
6	-7.090084000	0.056721000	-0.398333000
6	-5.664213000	-0.021689000	-0.430478000
6	-5.020737000	-1.278181000	-0.505490000
6	-9.322525000	-1.067804000	-0.331160000
6	-7.720901000	1.336009000	-0.328949000
6	-9.188867000	1.411855000	-0.274659000
6	-9.968895000	0.208169000	-0.220854000
6	-9.851412000	2.633823000	-0.260744000
6	-6.909821000	2.472547000	-0.295473000
6	-5.514186000	2.389557000	-0.337153000
6	-4.885720000	1.157946000	-0.400597000
1	-4.904464000	3.285944000	-0.328132000
1	-5.272537000	-3.386683000	-0.651002000
6	-10.106563000	-2.214856000	-0.327109000
6	-11.499101000	-2.144539000	-0.269364000
6	-12.159540000	-0.932261000	-0.159698000
6	-11.401070000	0.281002000	-0.063890000
1	-12.079225000	-3.057205000	-0.334932000
6	-11.223746000	2.706571000	-0.014176000
1	-11.709718000	3.670608000	0.091082000
6	-11.983541000	1.566606000	0.171102000
1	-7.352754000	3.458683000	-0.233975000
1	-9.307007000	3.560150000	-0.393751000
1	-7.727965000	-3.294012000	-0.612661000
1	-9.653373000	-3.195758000	-0.399404000
6	-3.405805000	1.098768000	-0.442012000
6	-3.542188000	-1.391197000	-0.470187000
7	-2.817586000	-0.180489000	-0.384759000
6	-1.380248000	-0.258910000	-0.229332000
6	-0.593995000	-0.868895000	-1.204362000
6	-0.798872000	0.291237000	0.913066000
6	0.788936000	-0.893267000	-1.054618000
6	0.583117000	0.253904000	1.071421000
6	1.372207000	-0.326112000	0.078096000
1	-1.052843000	-1.285940000	-2.089847000
1	-1.422736000	0.763504000	1.664331000
1	1.417609000	-1.312860000	-1.829124000
1	1.047096000	0.698007000	1.943467000
8	-2.722348000	2.112031000	-0.506130000
8	-2.972868000	-2.469287000	-0.493882000
6	-13.335883000	1.810822000	0.754546000

8	-14.025929000	2.781952000	0.525774000
8	-13.646506000	0.884923000	1.694109000
6	-13.644379000	-0.956874000	-0.301571000
8	-14.328017000	-0.098010000	-0.824647000
8	-14.173735000	-2.107640000	0.173523000
6	5.585409000	2.182030000	0.247429000
6	6.979866000	2.230627000	0.155354000
6	7.751156000	1.071202000	0.059529000
6	7.090943000	-0.193477000	0.084704000
6	5.665425000	-0.234934000	0.138906000
6	4.924422000	0.965516000	0.230687000
6	9.214131000	1.110956000	-0.069424000
6	7.826416000	-1.415797000	0.045834000
6	9.296677000	-1.367550000	0.073294000
6	9.973701000	-0.103206000	0.008374000
6	10.059532000	-2.526184000	0.157559000
6	7.112564000	-2.615463000	-0.001820000
6	5.713364000	-2.646003000	0.009449000
6	4.984351000	-1.472101000	0.091406000
1	5.176689000	-3.587599000	-0.038473000
1	4.999977000	3.092159000	0.320350000
6	9.885914000	2.308279000	-0.277149000
6	11.271659000	2.342933000	-0.434305000
6	12.045706000	1.201087000	-0.305246000
6	11.413549000	-0.050456000	0.006974000
1	11.750686000	3.275553000	-0.702677000
6	11.448541000	-2.466403000	0.300696000
1	12.019439000	-3.373235000	0.471310000
6	12.124841000	-1.260834000	0.289533000
1	7.636302000	-3.562504000	-0.055632000
1	9.585759000	-3.499949000	0.190263000
1	7.452783000	3.204654000	0.161569000
1	9.336441000	3.233390000	-0.396475000
6	3.502826000	-1.536676000	0.109614000
6	3.446151000	0.948036000	0.320491000
7	2.813500000	-0.301354000	0.186367000
8	2.899268000	-2.594352000	0.071522000
8	2.807265000	1.978196000	0.489724000
6	13.549863000	-1.347702000	0.738335000
8	14.288005000	-2.279036000	0.502028000
8	13.863128000	-0.333915000	1.580444000
6	13.479888000	1.292250000	-0.704517000
8	14.177538000	0.354001000	-1.040575000
8	13.918657000	2.569889000	-0.719637000
6	15.213067000	2.786863000	-1.307932000
1	15.479725000	3.812445000	-1.049399000
1	15.943714000	2.083303000	-0.901336000

1	15.150566000	2.667948000	-2.392362000
6	15.236776000	-0.308930000	1.996823000
1	15.327346000	0.555435000	2.655946000
1	15.498275000	-1.228864000	2.526552000
1	15.885888000	-0.201012000	1.122799000
6	-14.955838000	1.025349000	2.266711000
1	-15.714828000	0.948698000	1.482448000
1	-15.050889000	0.206572000	2.980884000
1	-15.056953000	1.991226000	2.768476000
6	-15.592520000	-2.245829000	-0.000554000
1	-15.858867000	-2.210539000	-1.060630000
1	-15.845284000	-3.216104000	0.427643000
1	-16.122311000	-1.444660000	0.522178000

Additional quantum chemical studies were made using the GAMESS suite of programs.[S16] These DFT calculations refer mostly to **PMIDE-m** in the ground-state and made use of the hybrid PBE0 functional[S17] and the 6-31D(G) basis set. All real harmonic vibrational frequencies were obtained for the energy optimized structures. Solvent effects being treated by the PCM protocol,[S18] with $CHCl_3$ as solvent for all studies, and using the Mennucci-Thomasi correction.[S19] As above, the solubilizing chains were replaced with methyl groups to speed up the calculations. The final structure obtained for the monomer is shown below in Figure S31 and the corresponding cartesian coordinates are listed in Table S9.

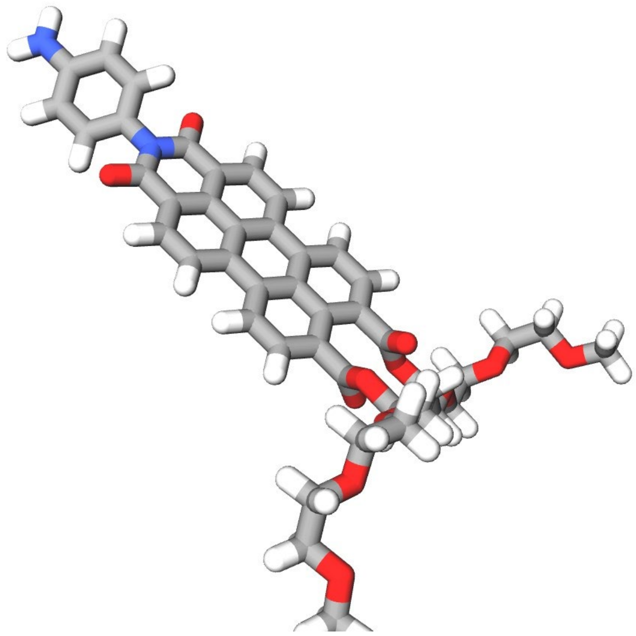

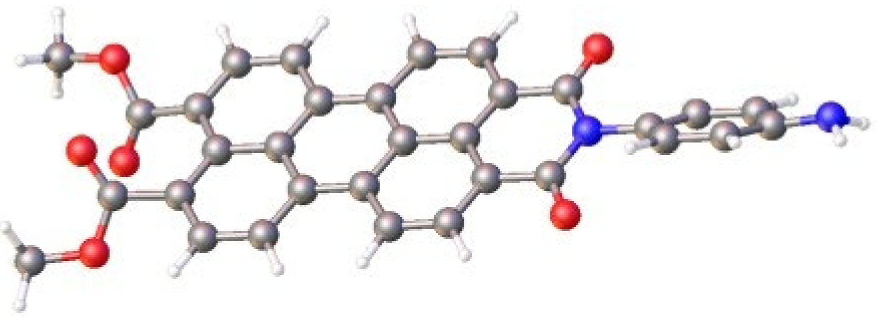

Figure S31. Computed structure for the model compound used to mimic **PMIDE-m**. Geometry optimization was made at the DFT/PBE0/6-311G(d,p) level with solvent effects being treated by the PCM ($CHCl_3$) method. The dihedral angle subtended between PMI and the phenyl ring is 62.3°. The imide carbonyl groups lie almost parallel with the plane of the polycycle, the mean torsion angle being 2.3°.

Table S9. Cartesian coordinates for the energy-minimized structure derived for the reference compound used to model **PMIDE-m** in $CHCl_3$.

C1	C	-0.30192	0.26432	0.30576	1.00000	0.05000
C2	C	-0.34374	0.34439	0.39815	1.00000	0.05000
C3	C	-0.40894	0.33700	0.38644	1.00000	0.05000
C4	C	-0.43733	0.24688	0.28105	1.00000	0.05000

C5	C	-0.33033	0.17220	0.19894	1.00000	0.05000
C6	C	-0.39756	0.16307	0.18754	1.00000	0.05000
C7	C	-0.29110	0.08920	0.10357	1.00000	0.05000
C8	C	-0.42654	0.06975	0.08246	1.00000	0.05000
C9	C	-0.38759	-0.01203	-0.01130	1.00000	0.05000
C10	C	-0.32240	-0.00166	-0.00090	1.00000	0.05000
C11	C	-0.22406	0.10054	0.11659	1.00000	0.05000
C12	C	-0.23471	0.27217	0.31524	1.00000	0.05000
C13	C	-0.09903	0.30079	0.34847	1.00000	0.05000
C14	C	-0.14347	0.37629	0.43588	1.00000	0.05000
C15	C	-0.20730	0.36321	0.42065	1.00000	0.05000
C16	C	-0.12317	0.20180	0.23379	1.00000	0.05000
C17	C	-0.19252	0.19169	0.22210	1.00000	0.05000
C18	C	-0.08722	0.11152	0.12914	1.00000	0.05000
C19	C	-0.18590	0.01901	0.02216	1.00000	0.05000
C20	C	-0.12155	0.02460	0.02851	1.00000	0.05000
C21	C	-0.50351	0.23803	0.26673	1.00000	0.05000
N1	N	-0.53071	0.14458	0.16487	1.00000	0.05000
C22	C	-0.49290	0.06029	0.07406	1.00000	0.05000
O1	O	-0.51704	-0.02918	-0.01809	1.00000	0.05000
O2	O	-0.53752	0.31898	0.34892	1.00000	0.05000
C23	C	-0.59575	0.13328	0.15328	1.00000	0.05000
C24	C	-0.62736	0.21779	0.04810	1.00000	0.05000
C25	C	-0.69276	0.19987	0.03341	1.00000	0.05000
C26	C	-0.72825	0.09795	0.12448	1.00000	0.05000
C27	C	-0.69774	0.01606	0.23089	1.00000	0.05000
C28	C	-0.63227	0.03343	0.24488	1.00000	0.05000
N2	N	-0.79081	0.07844	0.10965	1.00000	0.05000
C29	C	-0.03657	0.34504	0.39970	1.00000	0.05000
C30	C	-0.02030	0.08608	0.09968	1.00000	0.05000
O3	O	-0.02609	0.44309	0.51324	1.00000	0.05000
C31	C	0.03174	0.49590	0.57438	1.00000	0.05000

O4	O	0.02503	0.14084	0.16306	1.00000	0.05000
H1	H	-0.81163	0.13303	0.03543	1.00000	0.05000
H2	H	-0.81529	0.00923	0.17291	1.00000	0.05000
O5	O	0.00254	-0.00743	-0.00808	1.00000	0.05000
C32	C	0.06600	-0.04474	-0.04822	1.00000	0.05000
H3	H	-0.32885	0.41216	0.47858	1.00000	0.05000
H4	H	-0.43552	0.39864	0.45697	1.00000	0.05000
H5	H	-0.40630	-0.08050	-0.08918	1.00000	0.05000
H6	H	-0.29932	-0.06495	-0.07464	1.00000	0.05000
H7	H	-0.13176	0.44754	0.51835	1.00000	0.05000
H8	H	-0.23251	0.42500	0.49205	1.00000	0.05000
H9	H	-0.20310	-0.04886	-0.05639	1.00000	0.05000
H10	H	-0.10115	-0.04187	-0.04852	1.00000	0.05000
H11	H	-0.60262	0.29259	-0.01921	1.00000	0.05000
H12	H	-0.71438	0.26091	-0.04406	1.00000	0.05000
H13	H	-0.72304	-0.05730	0.29821	1.00000	0.05000
H14	H	-0.61112	-0.02882	0.32228	1.00000	0.05000
O6	O	0.01494	0.30490	0.35316	1.00000	0.05000
H15	H	0.05908	0.58927	0.51185	1.00000	0.05000
H16	H	0.02121	0.57224	0.66281	1.00000	0.05000
H17	H	0.05908	0.36823	0.59719	1.00000	0.05000
H18	H	0.08995	0.09186	-0.06156	1.00000	0.05000
H19	H	0.06330	-0.11580	-0.14561	1.00000	0.05000
H20	H	0.09100	-0.12424	0.03156	1.00000	0.05000

S5. Ultrafast transient absorption spectroscopy

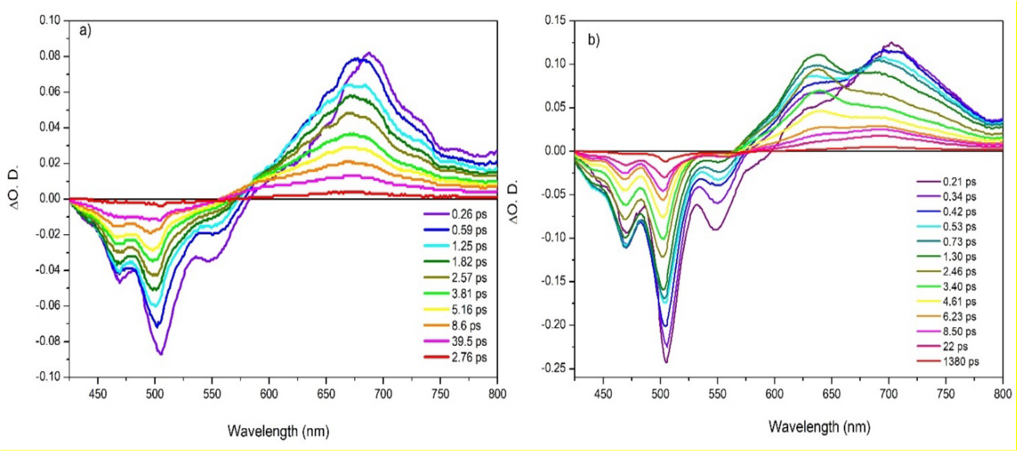

Figure S32. Transient differential absorption spectra recorded for **PMIDE-m** in (a) MeCN (C_T = 100 μM; α_D = 0.13; F_D = 4%) and (b) THF (C_T = 100 μM; α_D = 0.62; F_D = 48%) with λ_{exc} = 500 nm.

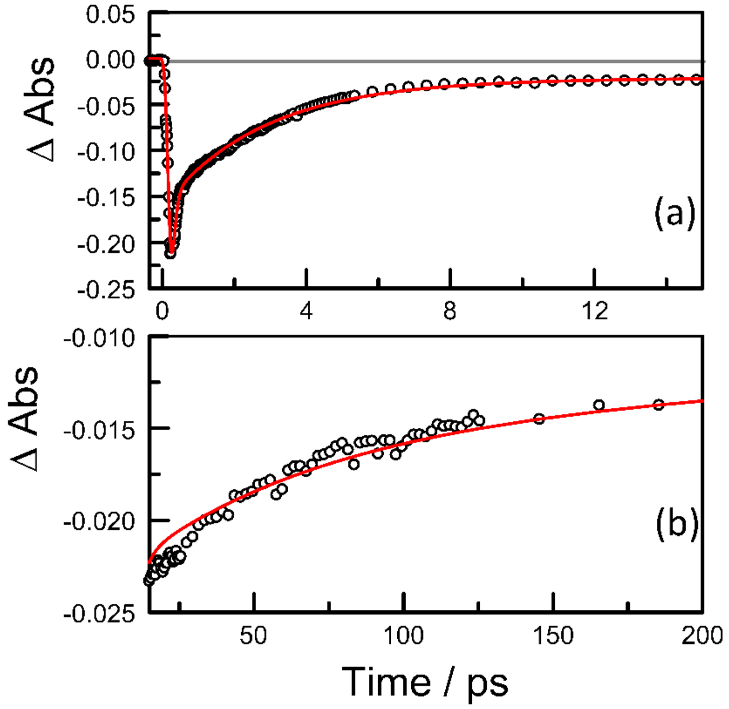

Figure S33. Decay kinetics monitored for **PMIDE-m** in THF (C_T = 100 μM; α_D = 0.62; F_D = 48%) at 511 nm on (a) short and (b) long timescales (λ_{exc} = 500 nm). The red line corresponds to a nonlinear least-squares fit to the model described in the text while the open circles refer to the experimental data.

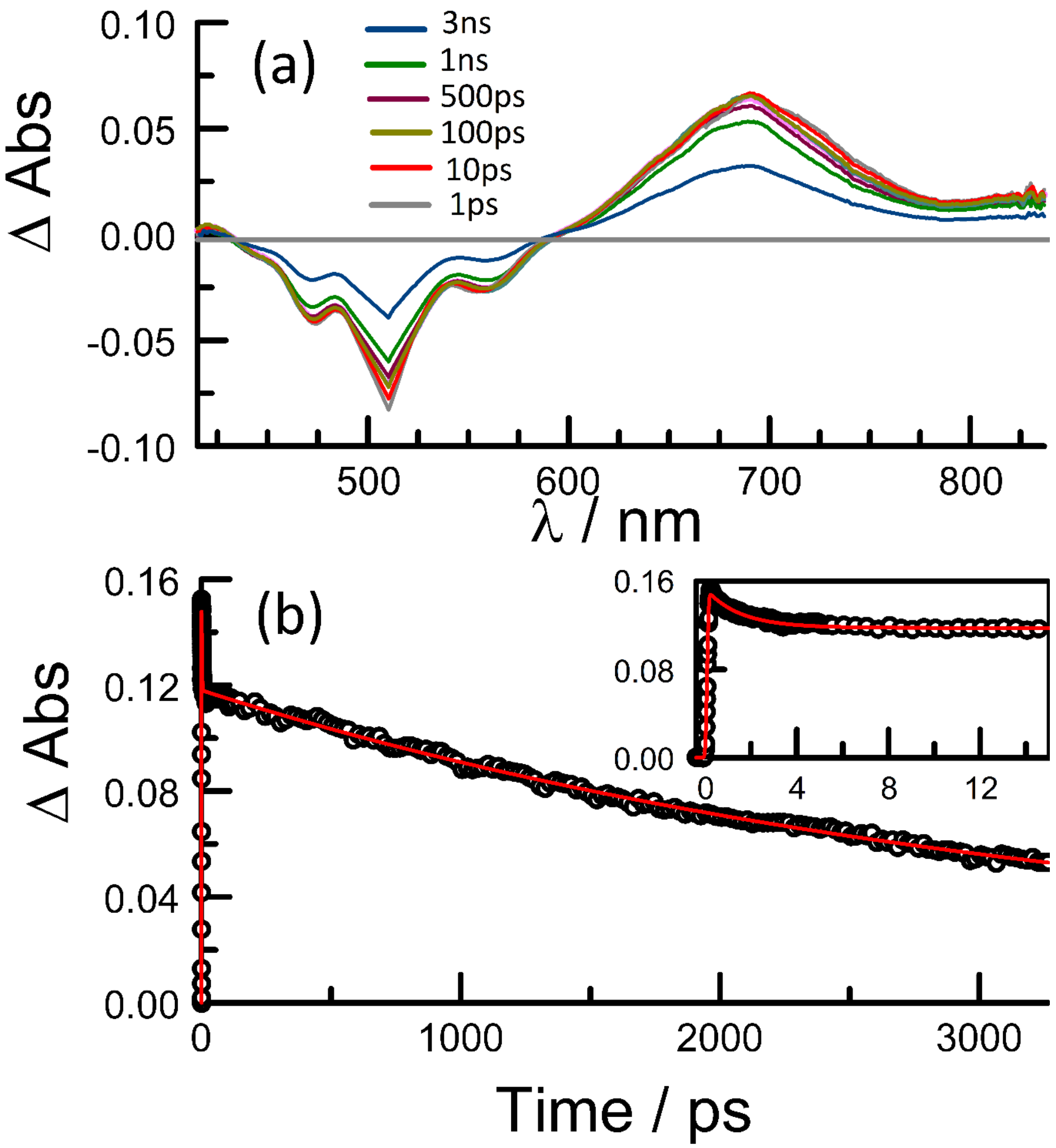

Figure S34. (a) Selection of transient absorption spectra recorded following excitation at 500 nm of **PMIDE-m** in THF at low concentration (C_T = 12 μM; α_D = 0.06; F_D = 2%). The spectral region around 500 nm suffers from contamination by scattered excitation light. (b) Example of a kinetic profile recorded at 720 nm. The experimental points appear as open circles while the calculated dual-exponential fit is shown as a red line running through the data points. The insert shows an expansion of the early time region, emphasizing conversion of S_{FC} to S_1. Note that lifetimes were determined from global analysis.

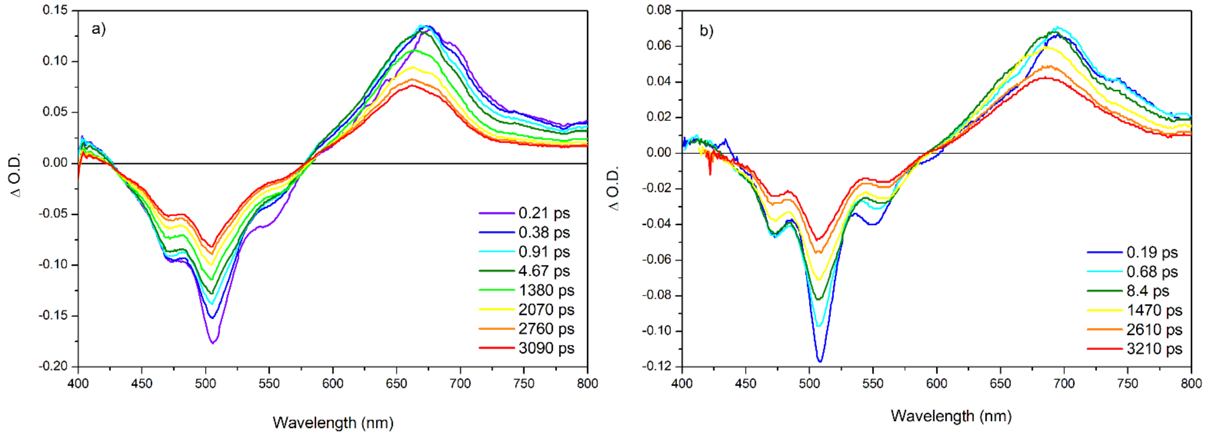

Figure S35. Transient absorption spectra recorded for **PMIDE-*m*-d** in (a) MeCN (C_T = 40 μM; α_D = 0.08; $F_D \approx$ 3%) and (b) THF (C_T = 30 μM; α_D = 0.02; $F_D \approx$ 1%). The excitation wavelength was 500 nm.

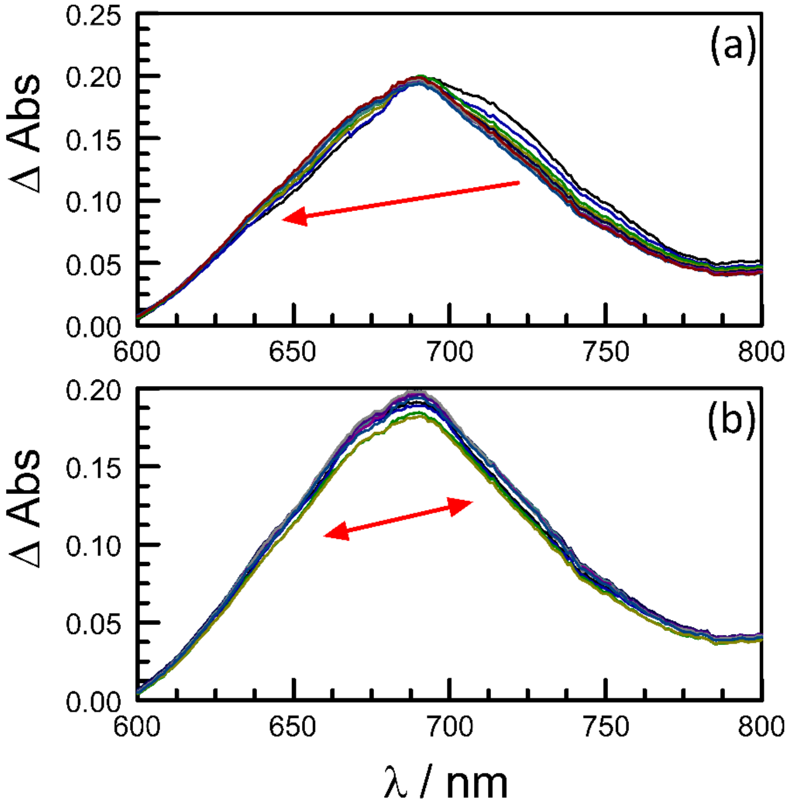

Figure S36. Spectral changes monitored for **PMIDE-*m*-d** in THF (C_T = 30 μM; α_D = 0.02; $F_D \approx$ 1%) (a) at early times and (b) at intermediate times following excitation at 500 nm. Delay times are (a) 0.5, 1, 2, 3, 5, 8, 12, 16, 20, 25 and 30 ps and (b) 50, 75, 100, 125, 150, 200, 250, 300, 400 and 500 ps. The arrow indicates the generic effect of increasing delay time.

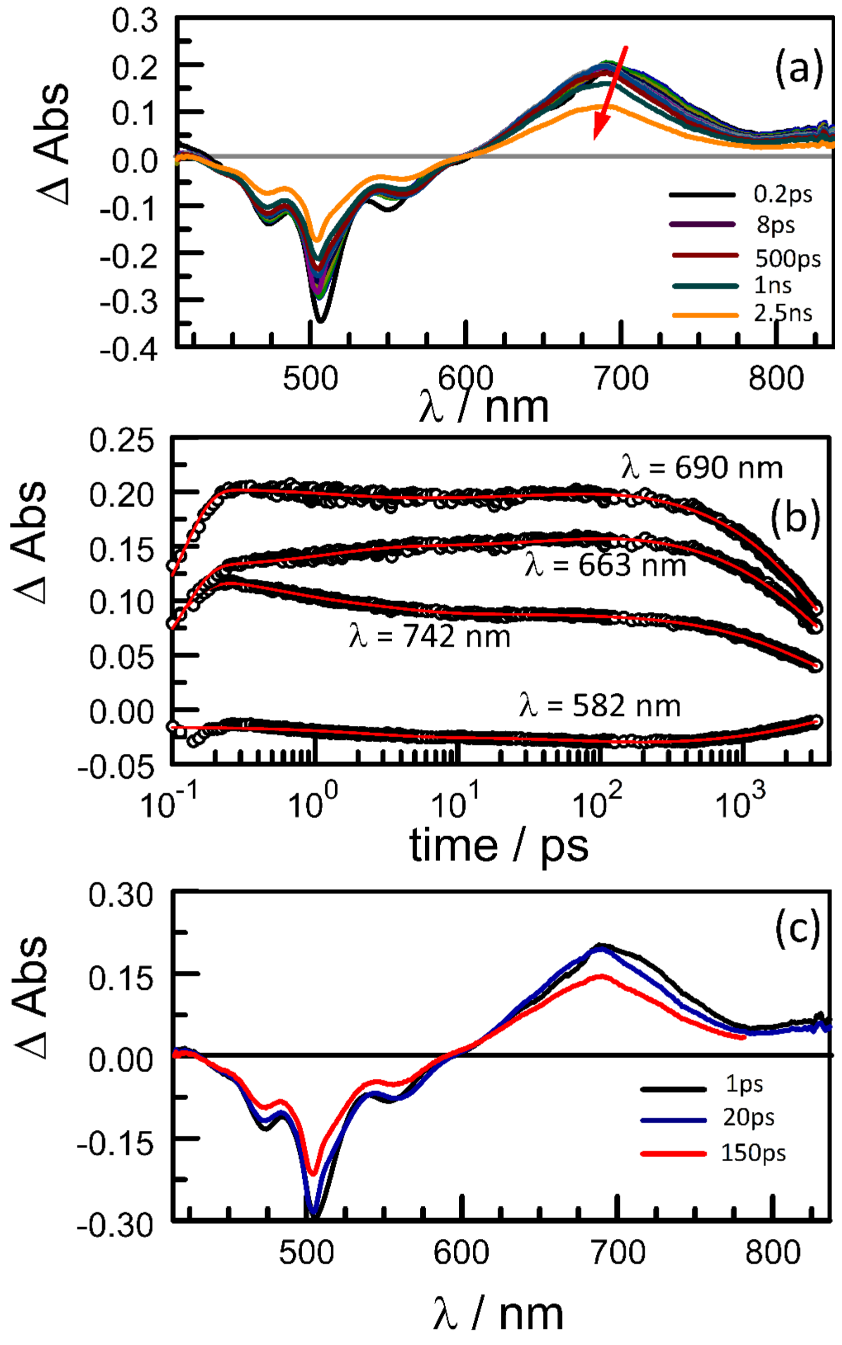

Figure S37. (a) Differential absorption spectra recorded after laser excitation at 500 nm of **PMIDE-*m*-d** in THF (C_T = 30 μM; α_D = 0.02; $F_D \approx$ 1%) . Individual spectra weere recorded at delay times of 0.2, 0.4, 0.6, 1, 2, 4, 8, 20, 50, 100, 500, 1,000 and 2,500 ps. The arrow indicates the direction of increasing delay time. (b) Kinetic plots constructed at various wavelengths for the spectra indicated in (a). The experimental points are shown at open circles and the kinetic fits appear as red lines. The derived lifetimes arise from global fitting of the experimental data. (c) SADS derived for the three main species involved in the decay processes and assigned to S_{FC} (black curve), S_1 (blue curve) and the equilibrium mixture of S_1 and the CTS (red curve).

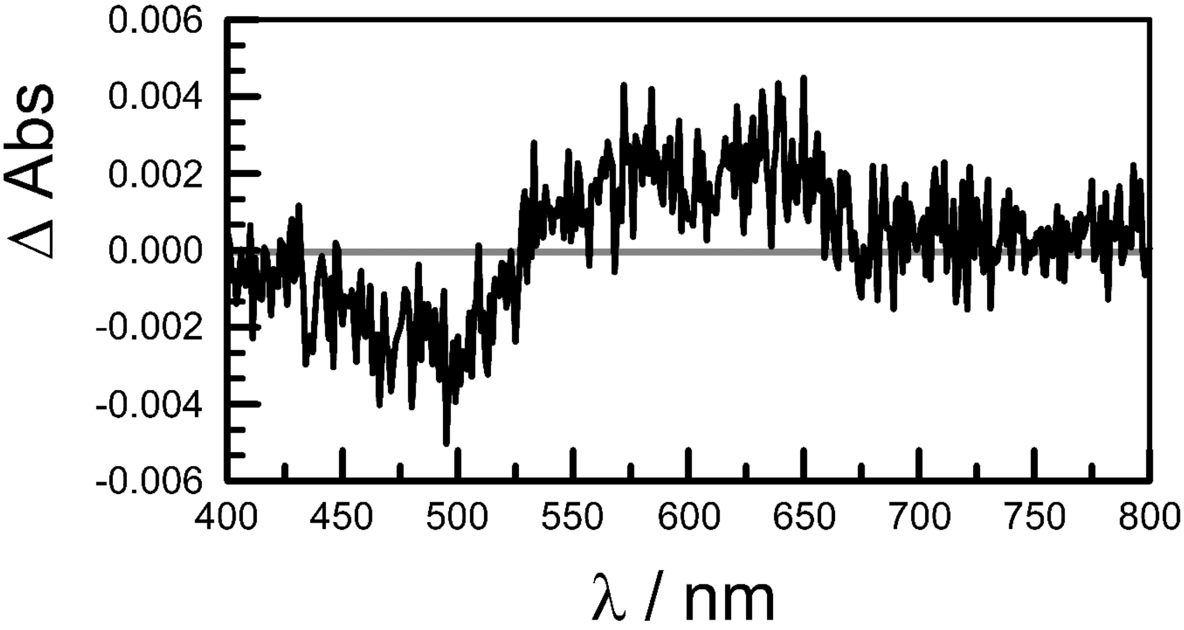

Figure S38. Transient differential absorption spectrum derived for the CTS involved in the excited-state behavior for **PMIDE-*m*-d** in THF solution. The spectrum was obtained from Figure S37c after isolated of the known spectrum for S_1. A reiterative non-linear, least squares fit, using the Levenberg-Marquardt algorithm, was used to discrimate the two overlapping spectra. The final spectrum is the result of averaging ten individual spectra recorded at different delay times.

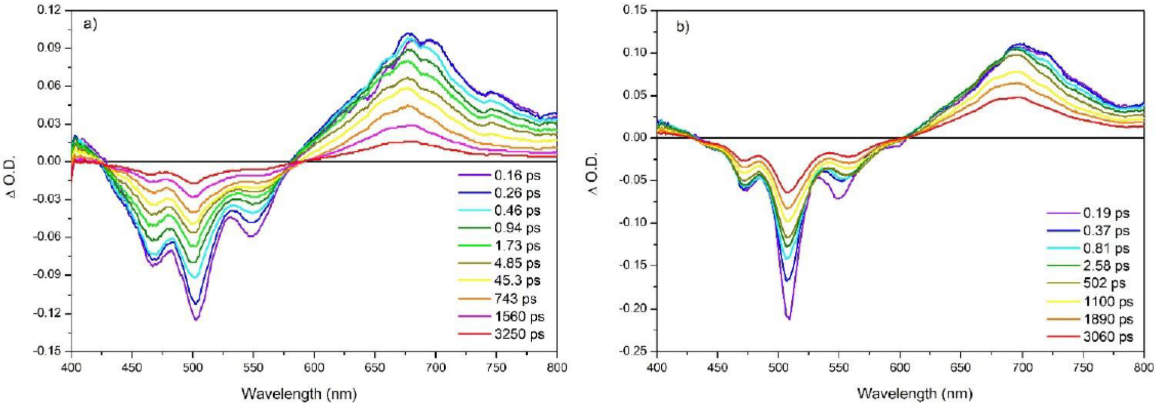

Figure S39. Transient absorption spectra recorded for **PMIDE-*p*-d** in (a) MeCN (C_T = 50 µM; α_D = 0.15; $F_D \approx$ 4%) and (b) THF (C_T = 30 µM; α_D = 0.30; $F_D \approx$ 7%). The excitation wavelength was 500 nm.

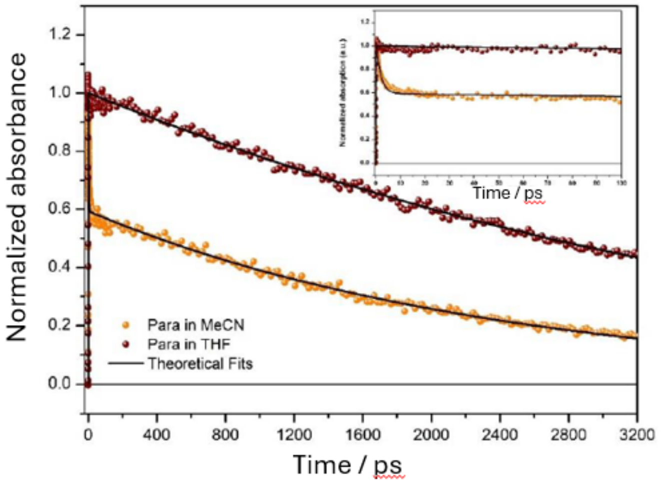

Figure S40. Examples of decay profiles recorded at 677 nm following laser excitation of **PMIDE-*p*-d** in MeCN (C_T = 50 µM; α_D = 0.15; $F_D \approx$ 4%) and THF (C_T = 50 µM; α_D = 0.15; $F_D \approx$ 4%). The excitation wavelength was 500 nm. The fast decay step observed for P**MIDE-*p*-d** in MeCN is attributed to relaxation of S_{FC} to give S_1. The same process occurs in THF but at slightly longer wavelength.

.

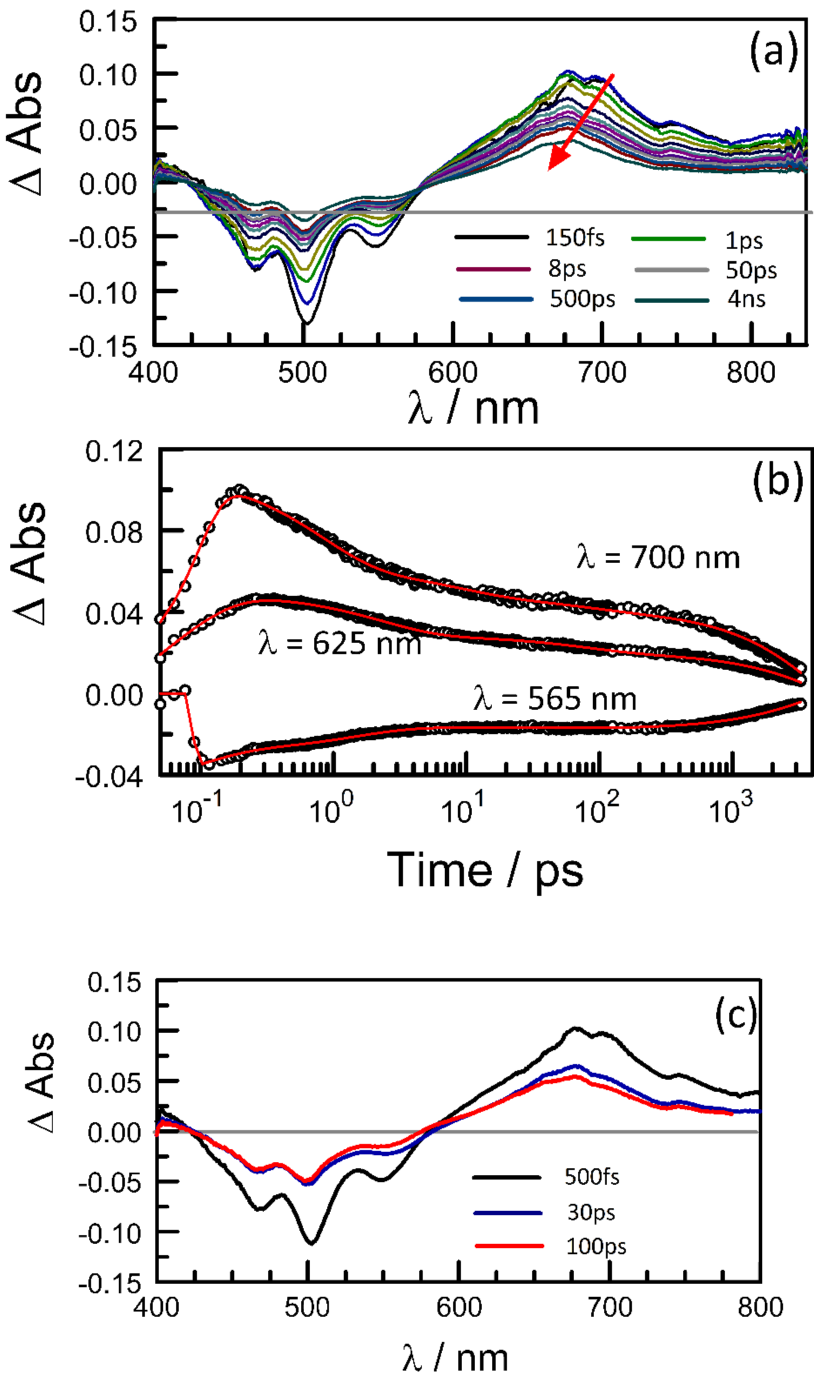

Figure S41. (a) Differential absorption spectra recorded after laser excitation at 500 nm of **PMIDE-*p*-d** in MeCN (C_T = 50 µM; α_D = 0.15; $F_D \approx$ 4%). Individual spectra were recorded at delay times of 0.15, 0.25, 0.5, 1, 2, 4, 8, 20, 50, 200, 500, 2.000 and 4,000 ps. The arrow indicates the direction of increasing delay time. (b) Kinetic plots constructed at various wavelengths for the spectra indicated in (a). The experimental points are shown at open circles and the kinetic fits appear as red lines. The derived lifetimes arise from global fitting of the experimental data. (c) SADS derived for the three main species involved in the decay processes and assigned to S_{FC} (black curve), S_1 (blue curve) and the equilibrium mixture of S_1 and the CTS (red curve).

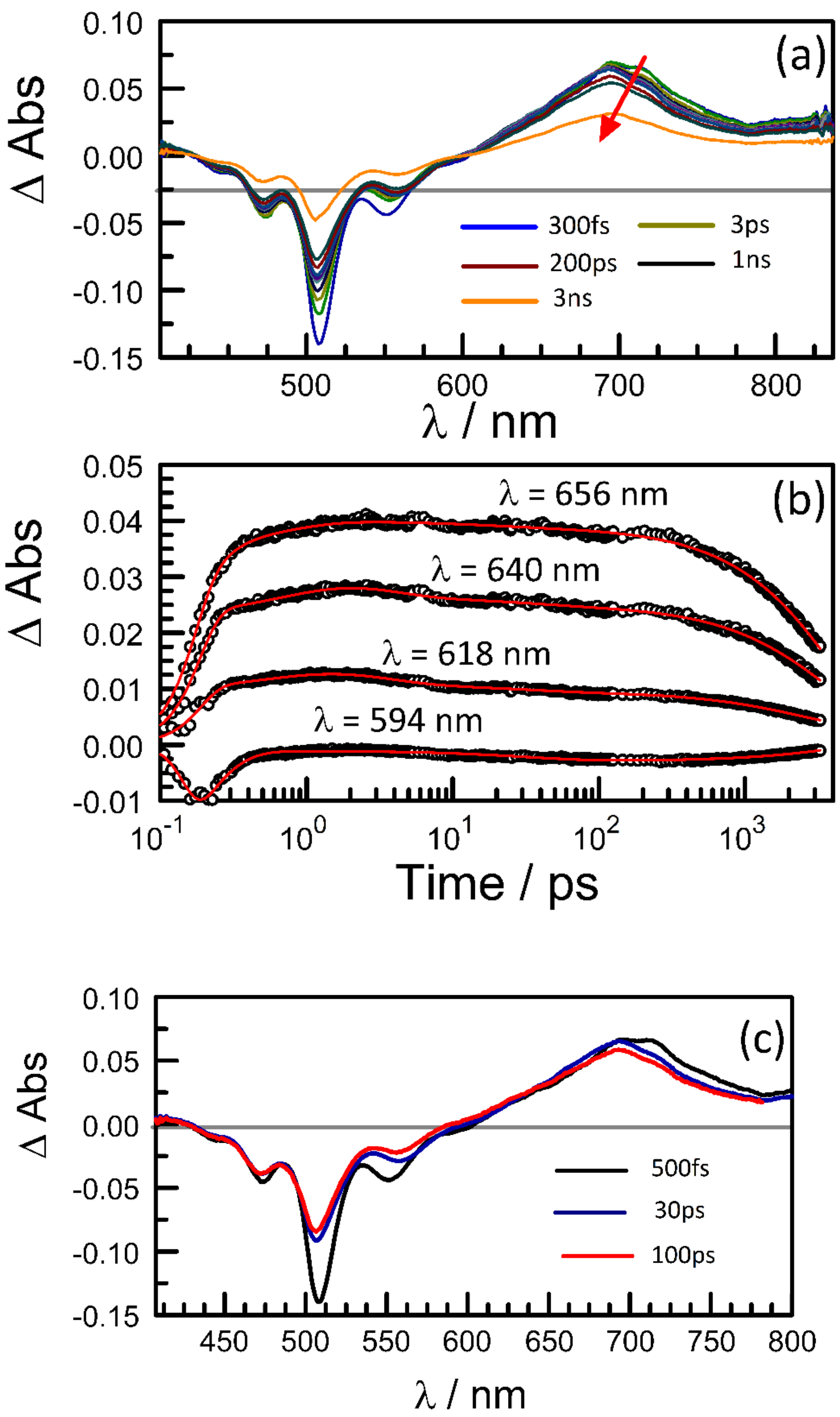

Figure S42. (a) Differential absorption spectra recorded after laser excitation at 500 nm of **PMIDE-*p*-d** in THF (C_T = 20 μM; α_D = 0.06; $F_D \approx$ 2%). Individual spectra were recorded at delay times of 0.3, 0.5, 1, 2, 4, 8, 20, 200, 500, 1,000 and 3,000 ps. The arrow indicates the direction of increasing delay time. (b) Kinetic plots constructed at various wavelengths for the spectra indicated in (a). The experimental points are shown at open circles and the kinetic fits appear as red lines. The derived lifetimes arise from global fitting of the experimental data. (c) SADS derived for the three main species and assigned to S_{FC} (black curve), S_1 (blue curve) and the equilibrium mixture of S_1 and the CTS (red curve).

S6. Time-resolved fluorescence

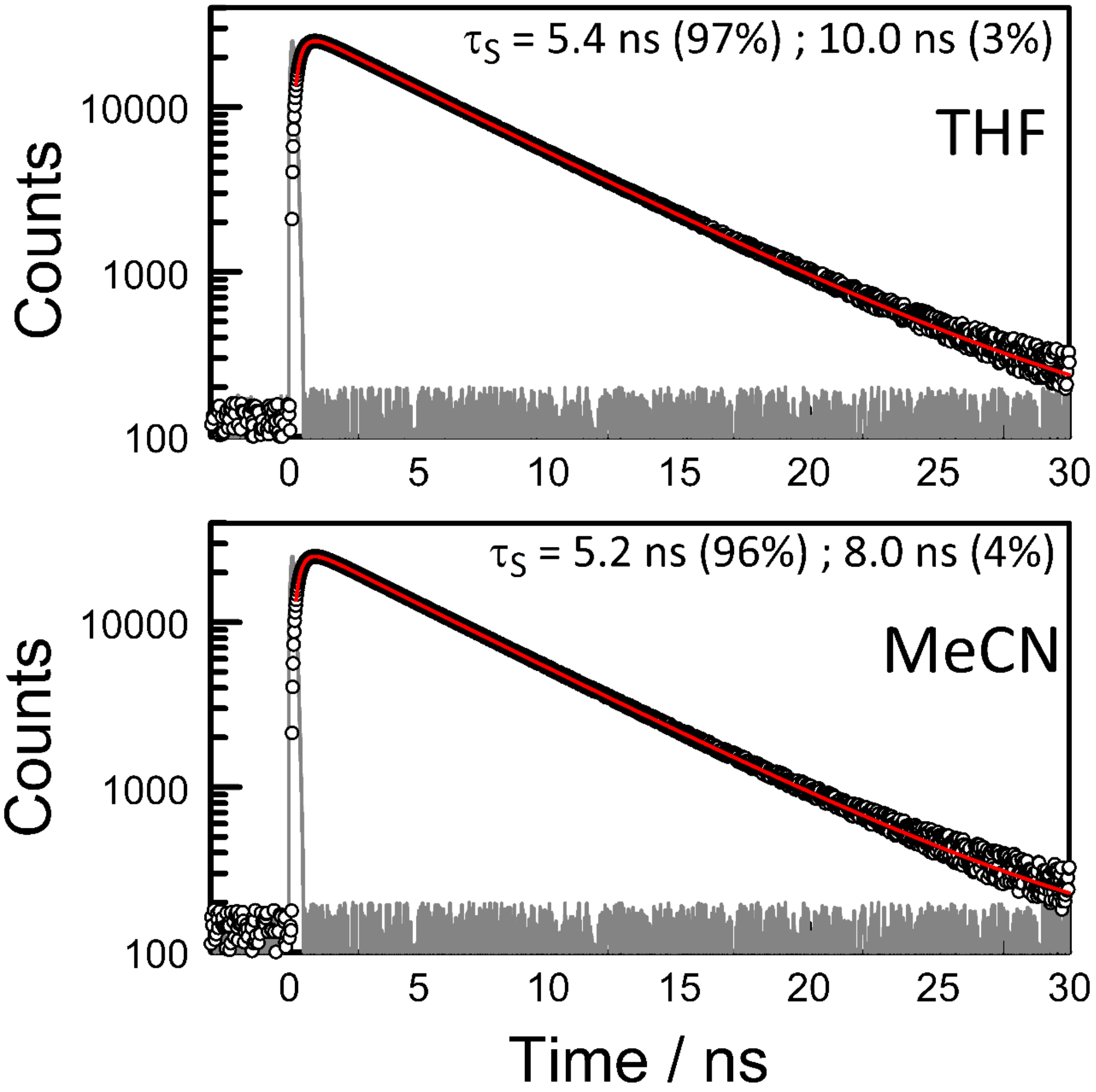

Figure S43. Examples of fluorescence decay profiles recorded for **PMIDE-m** by time-correlated, single photon counting following excitation at 440 nm. The instrumental response function is shown as a grey curve while the experimental data appear as open circles. The fit to the sum of two exponential components is shown in red and the derived numerical parameters are indicated on the figure.

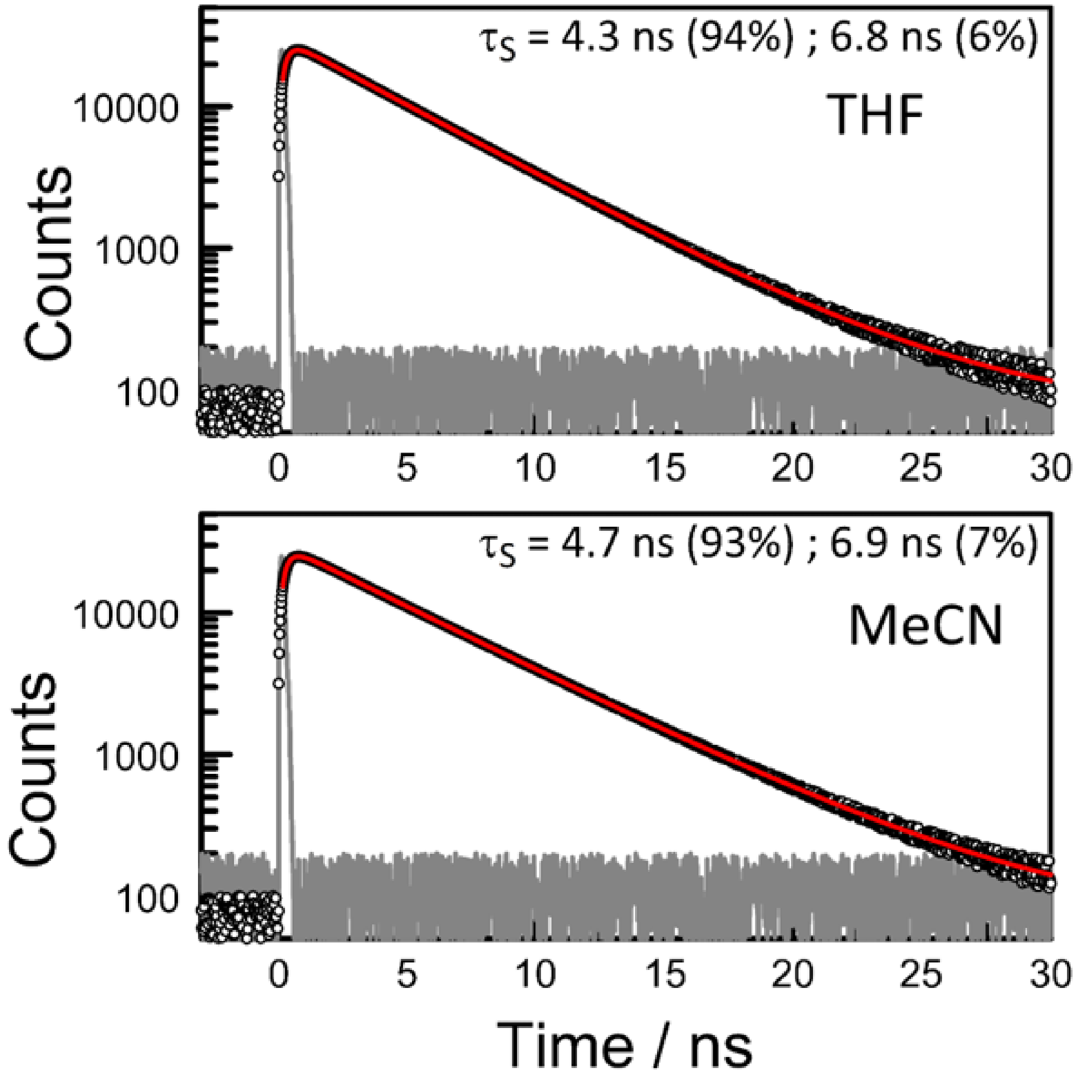

Figure S44. Examples of fluorescence decay profiles recorded for **PMIDE-m-d** by time-correlated, single photon counting following excitation at 440 nm. The instrumental response function is shown as a grey curve while the experimental data appear as open circles. The fit to the sum of two exponential components is shown in red and the derived numerical parameters are indicated on the figure.

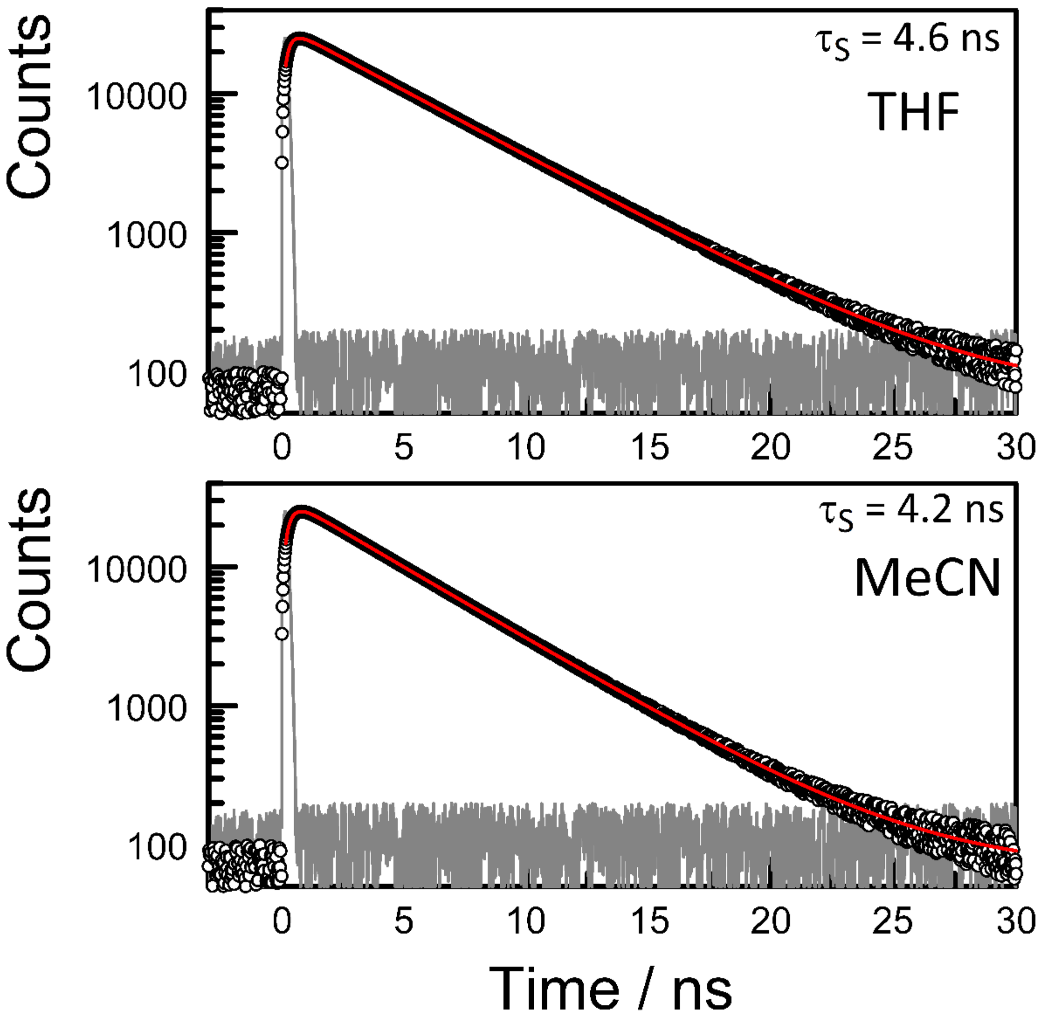

Figure S45. Examples of fluorescence decay profiles recorded for **PMIDE-p-d** by time-correlated, single photon counting following excitation at 440 nm. The instrumental response function is shown as a grey curve while the experimental data appear as open circles. The fit to a single exponential component is shown in red and the derived numerical parameters are indicated on the figure.

S7. Nanosecond transient absorption spectroscopy

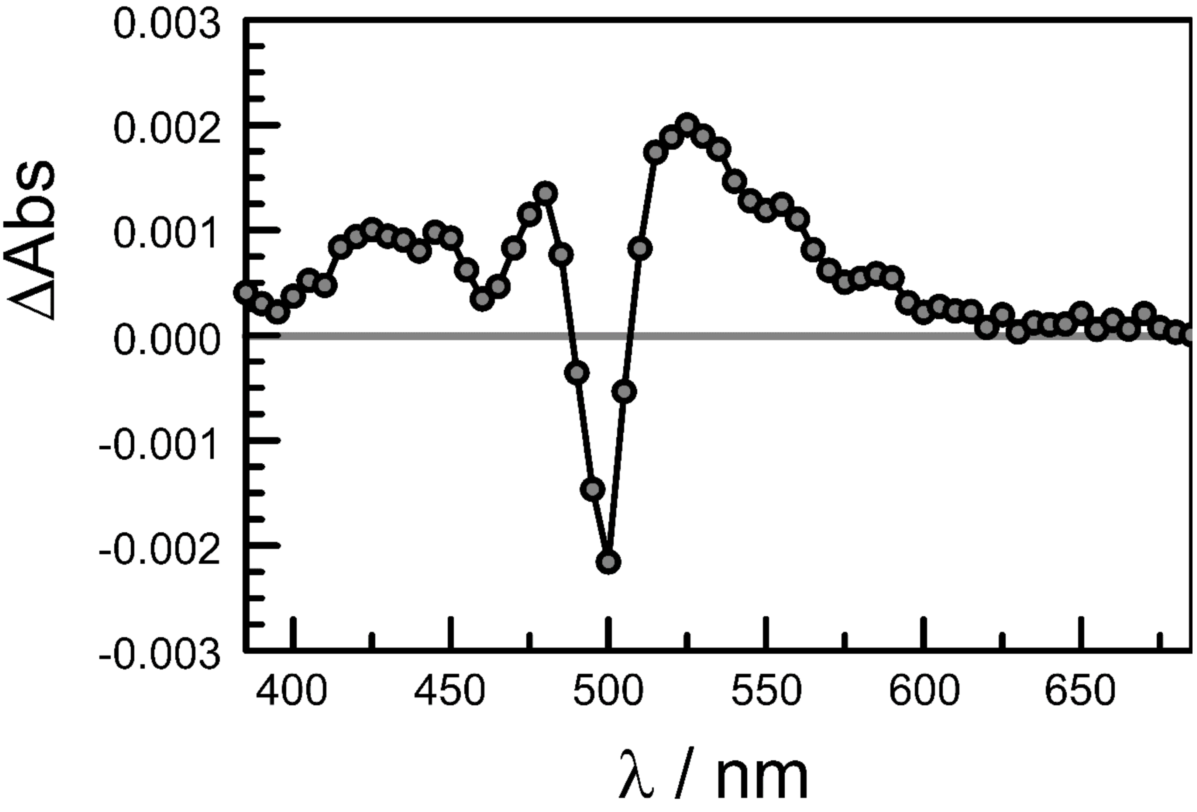

Figure S46. Example of a transient differential absorption spectrum recorded after laser excitation of **PMIDE-m** in MeCN (10 µM). The excitation pulse, for which the FWHM was 4 ns, was delivered at 440 nm and the spectrum was measured at a time delay of 100 ns. The solution was deaerated before the experiment. The filled circles correspond to the experimental data points joined by a straight line.

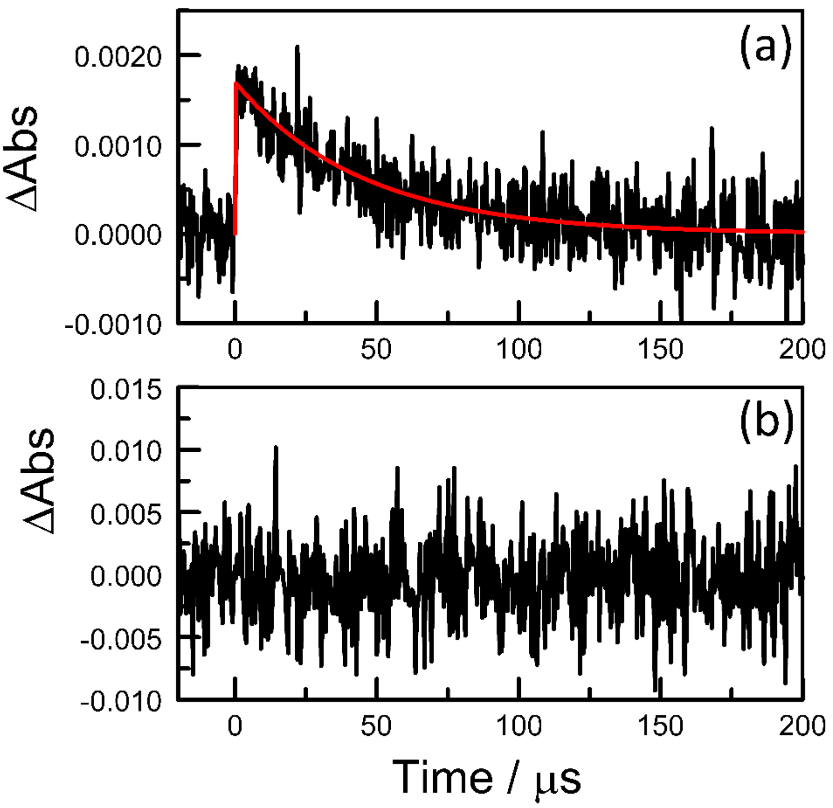

Figure S47. Examples of kinetic traces recorded at 535 nm following laser excitation of **PMIDE-m** in (a) MeCN and (b) THF, after deaeration of the solution with a stream of dried N_2. For (a), the red curve drawn through the experimental points corresponds to an exponential fit with a lifetime of 44.2 μs. For (b), the record shows no appreciable formation of a transient species on the μs timescale.

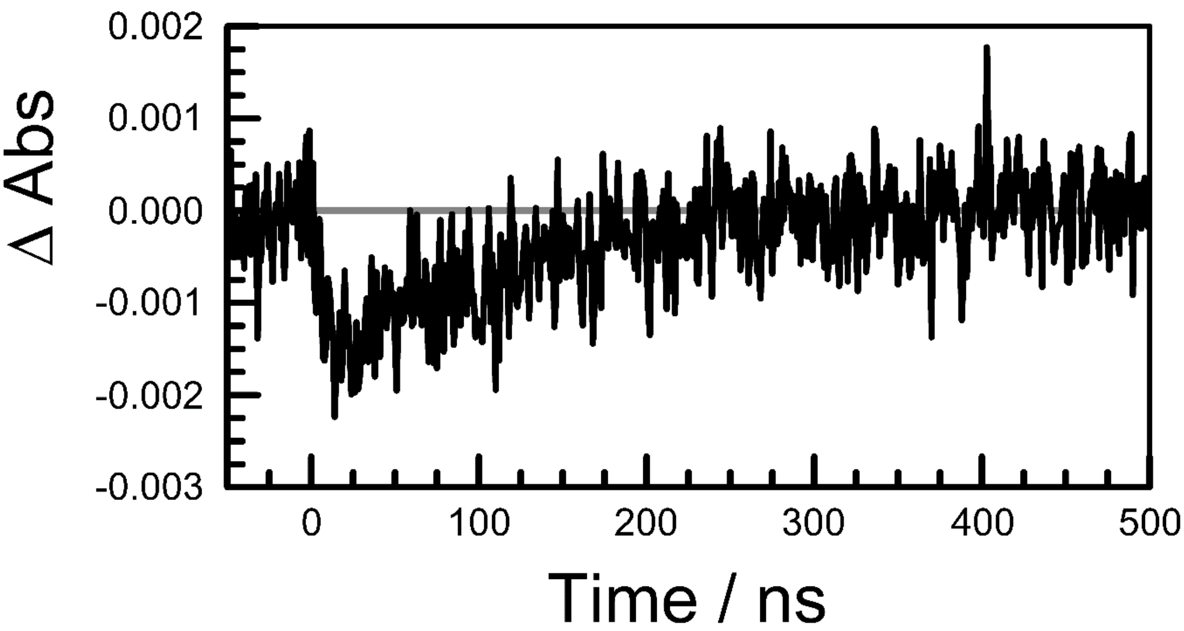

Figure S48. Kinetic profile recorded at 500 nm following excitation of **PMIDE-m** in THF (C_T = 100 µM; α_D = 0.62; F_D = 48%) with a 4-ns laser pulse delivered at 460 nm. The approximate lifetime of the transient bleaching is 90 ns. The optical pathlength was 1 mm.

S8. Charge-transfer state

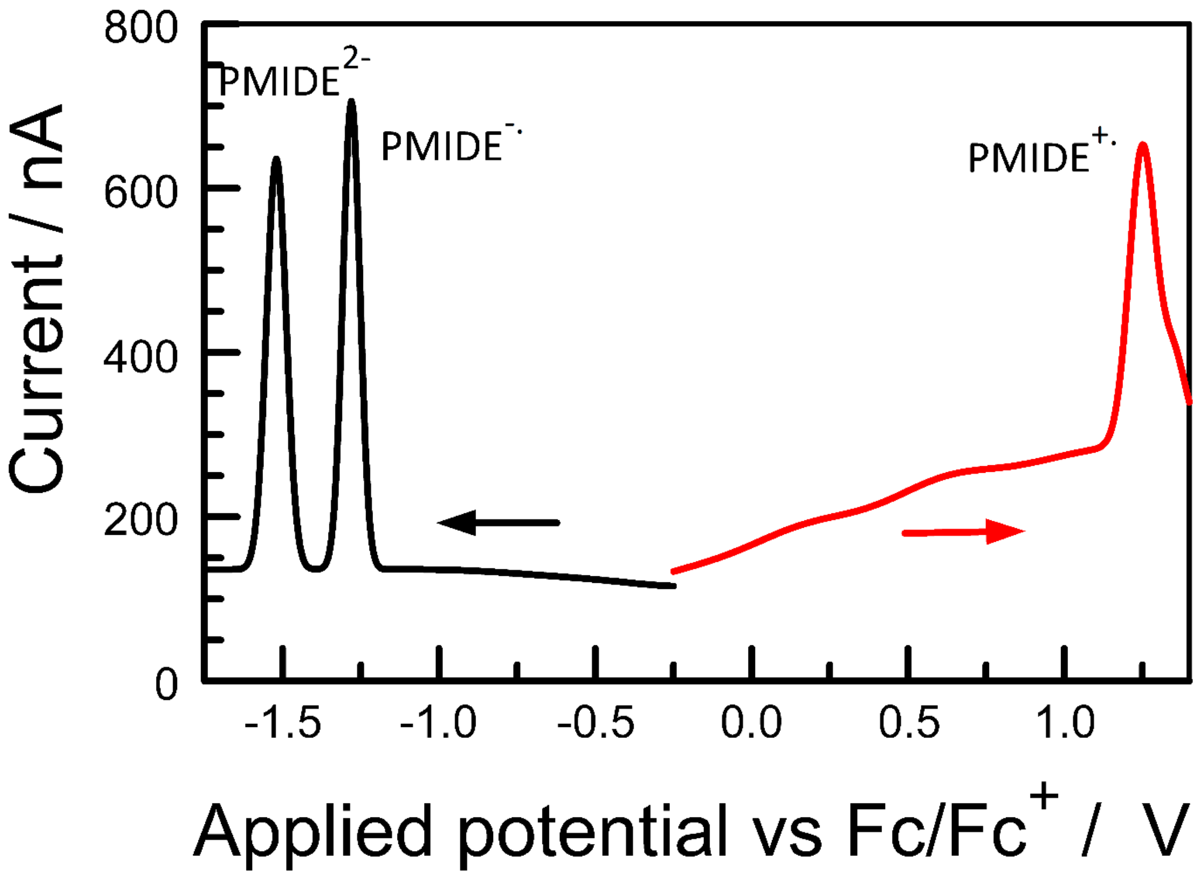

Figure S49. Differential pulse voltammogram recorded for **PMIDE-m-d** in freshly distilled CH_2Cl_2 after displacement of the dissolved air with argon. The initial solute concentration was *ca*. 0.5 mM and the solution contained tetrabutylammonium hexafluorophosphate (0.1 M) as supporting electrolyte. The arrows indicate the direction of the scan. The broad "peak" seen at around 0.5 V vs Fc/Fc^+ is attributed to adsorption onto the glassy carbon working electrode.

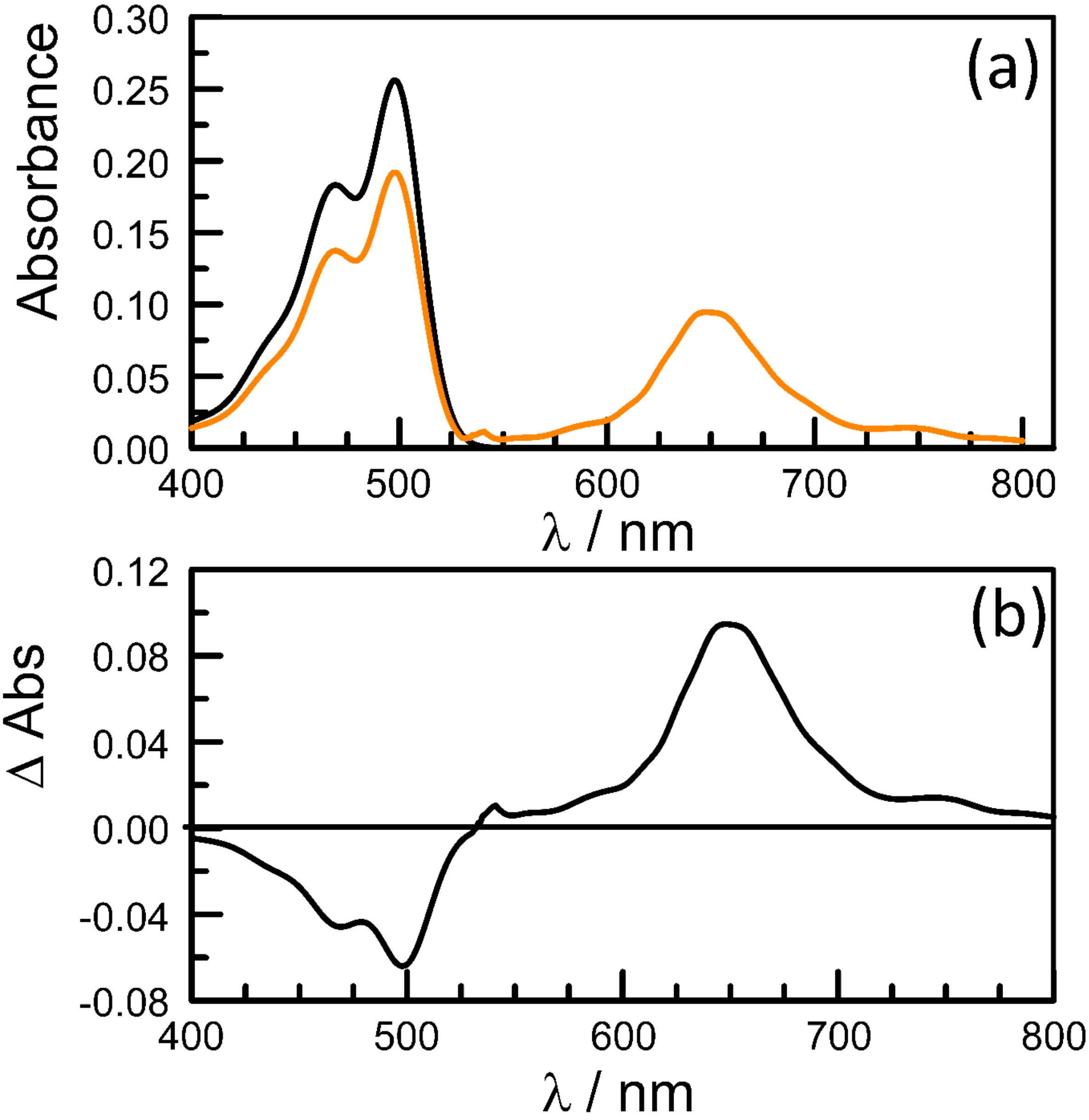

Figure S50. (a) Absorption spectra recorded before (black curve) and after (orange curve) electrolysis of **PMIDE-m** in freshly distilled MeCN after displacement of the dissolved air with argon. The solute concentration was *ca.* 1 mM and the solution contained tetrabutylammonium hexafluorophosphate (0.1 M) as supporting electrolyte. The applied potential was -1.2 V vs Fc/Fc^{+}. The experiment was performed with the sample housed in an OTTLE. (b) Corresponding differential spectrum for the π-radical anion. Assuming the latter does not contribute towards the absorbance change at 500 nm, the molar absorption coefficient for the anion radical at the maximum can be determined by reference to ground-state bleaching. The averaged value is 73,250 M^{-1} cm^{-1}.

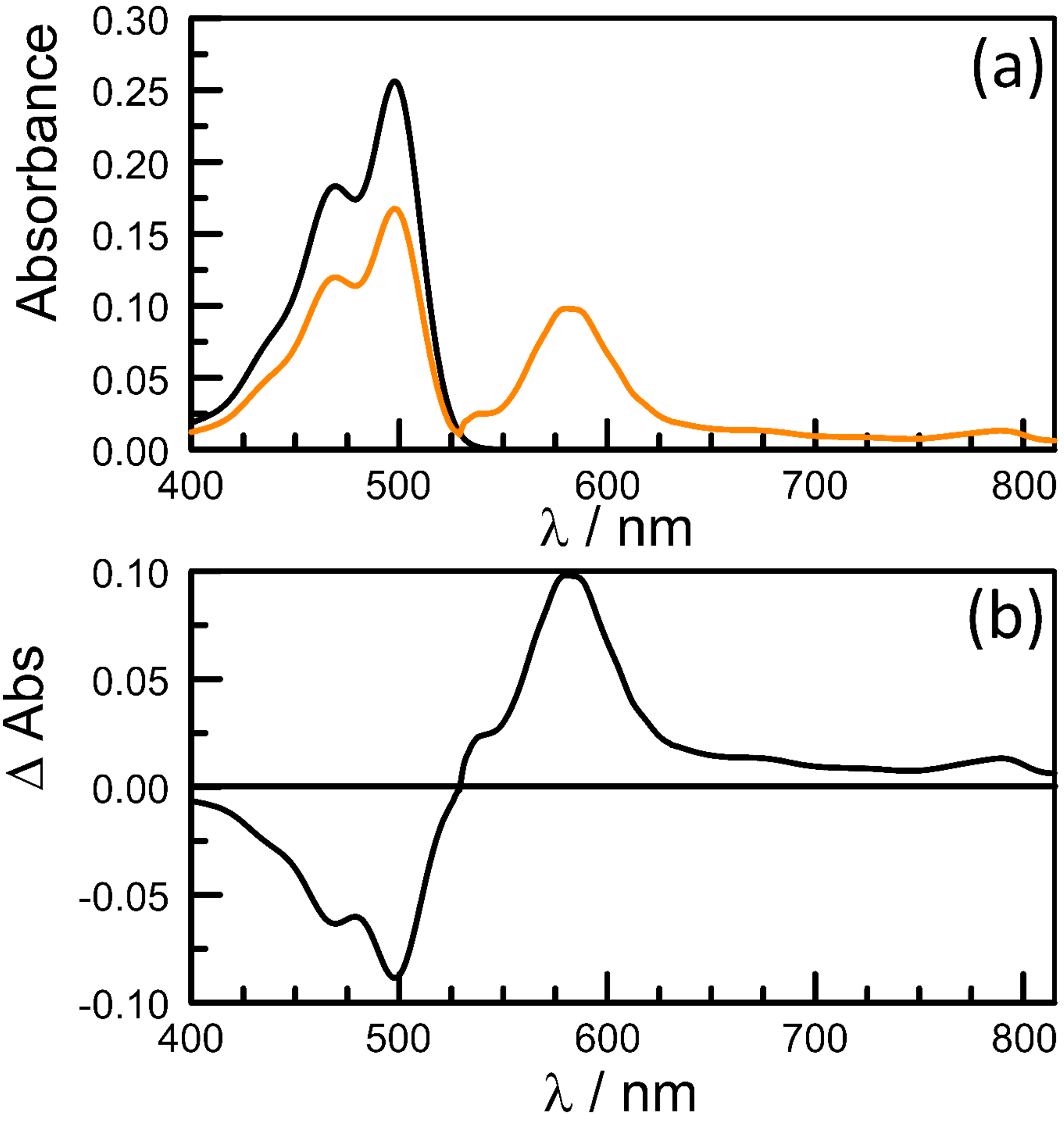

Figure S51. (a) Absorption spectra recorded before (black curve) and after (orange curve) photolysis (λ > 450 nm) of **PMIDE-m** in freshly distilled CH_2Cl_2 after saturation with dried air. The solute concentration was *ca*. 15 μM. The light source was a high intensity xenon flash lamp and the spectrum was recorded with a rapid scan spectrophotometer. The final spectrum is the result of numerous averages. (b) Corresponding differential spectrum for the π-radical cation. Assuming the latter does not contribute towards the absorbance change at 500 nm, the molar absorption coefficient for the cation radical at the maximum can be determined by reference to ground-state bleaching. The averaged value is 56,500 M^{-1} cm^{-1}.

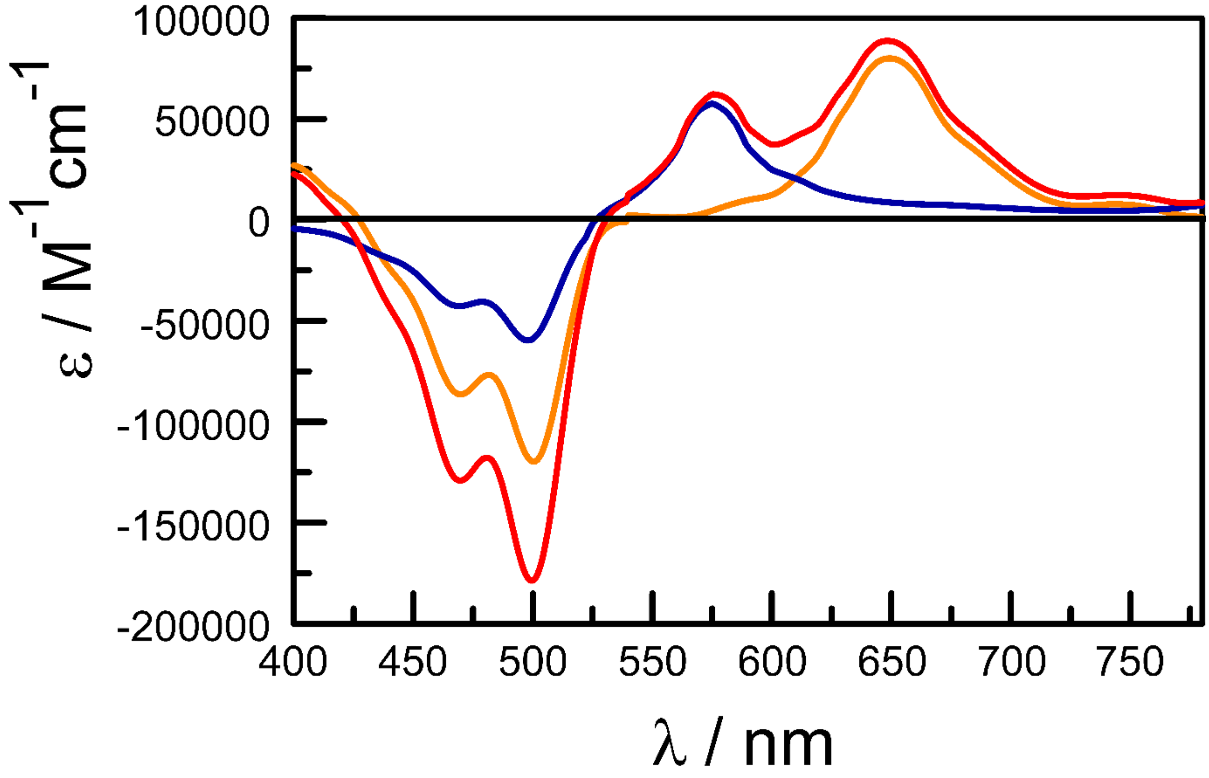

Figure S52. Compilation of the differential absorption spectrum derived for the idealized charge-transfer states (CTS) for **PMIDE-m-d** in MeCN (red curve) by summation of the individual spectra. The molar absorption coefficients are based on the π-radical anion (orange curve) and the π-radical cation (blue curve) making no contribution to the spectrum at 500 nm.

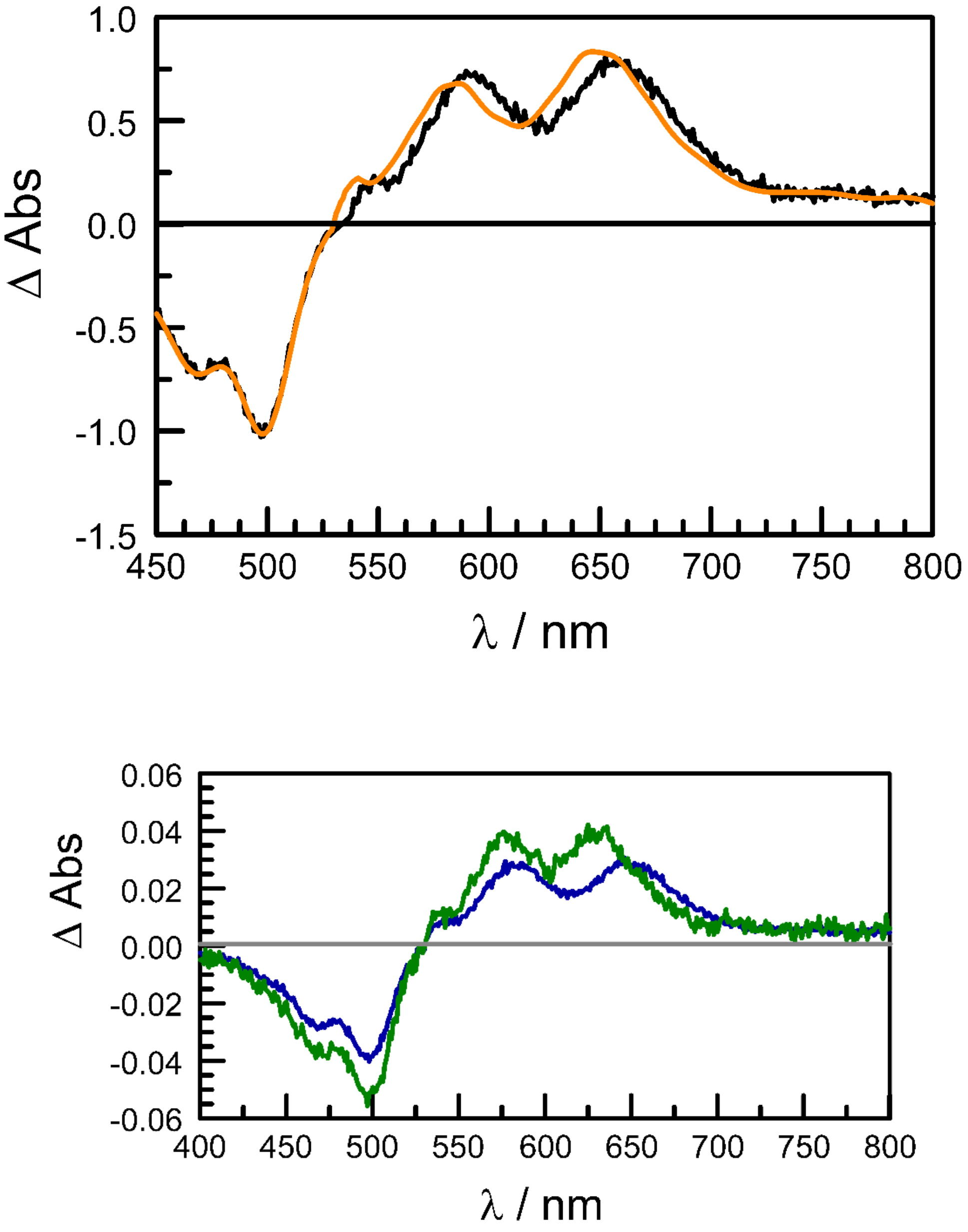

Figure S53. The upper panel shows a comparison of differential absorption spectra derived for the CTS state formed with **PMIDE-m-d** in MeCN (black curve) and the projected spectrum for the charge-separated state (CSS) as derived from spectroelectrochemical and photochemical measurements made with **PMIDE-m** (orange curve). The latter was compiled from individual spectra for radical anion and radical cation on the basis of zero electronic interaction between the radicals and assuming no absorption at 500 nm. The two spectra were normalized at 500 nm. The lower panel shows a comparison of the derived absorption spectra for the CTS from **PMIDE-*p*-d** in THF (green curve) and MeCN (blue curve).

S8 ^{1}H NMR, ^{13}C NMR, and mass spectra

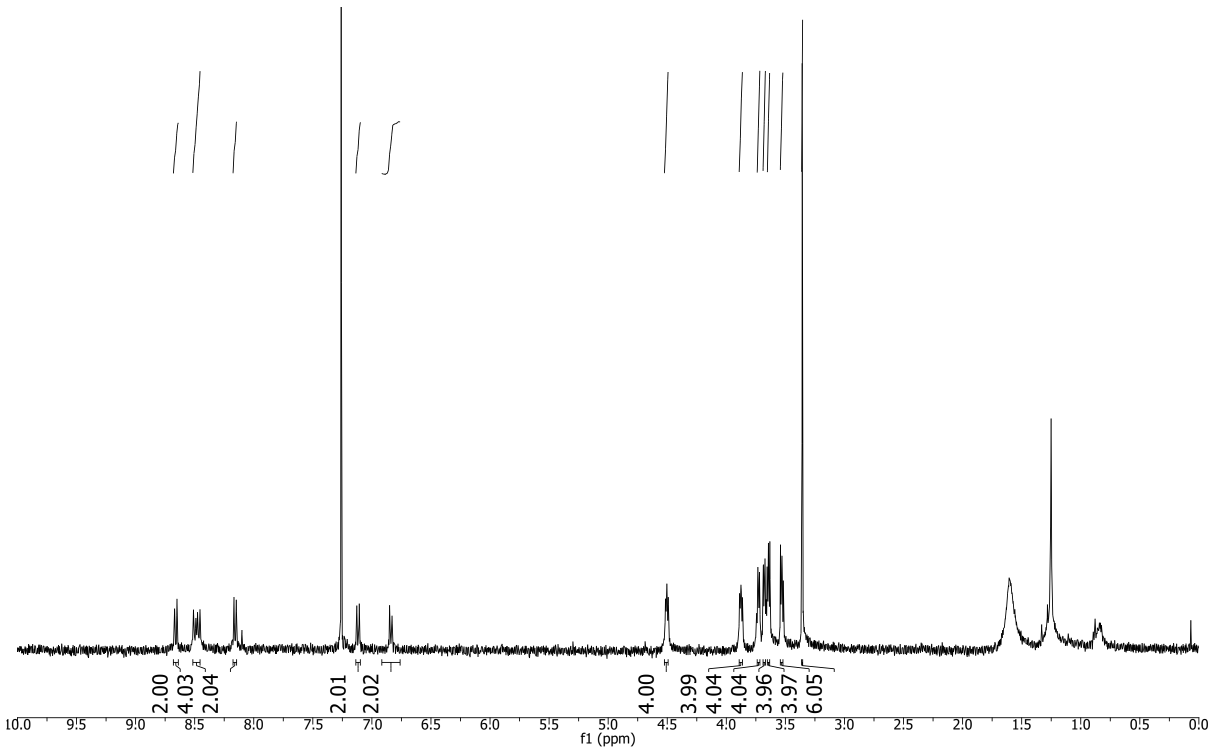

Figure S54. ^{1}H-NMR spectrum (400 MHz, $CDCl_3$, 298 K) of **PMIDE-m**.

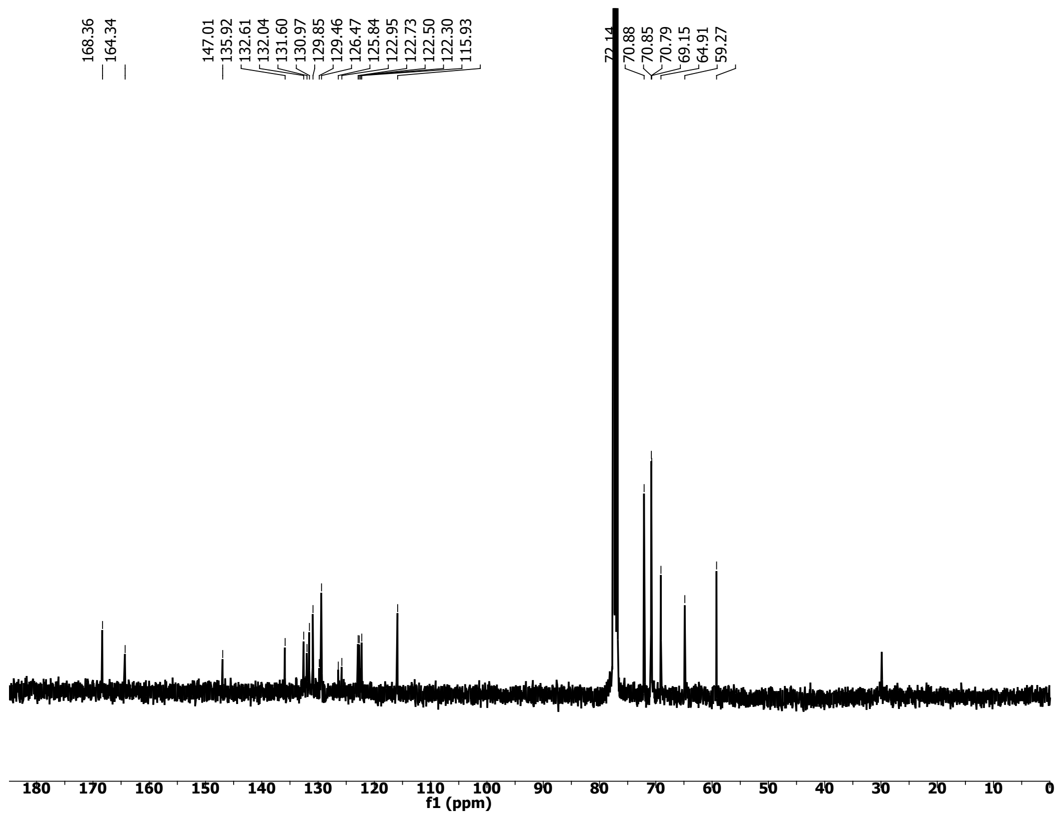

Figure S55. ^{13}C-NMR spektrum (100 MHz, $CDCl_3$, 298 K) of **PMIDE-m**.

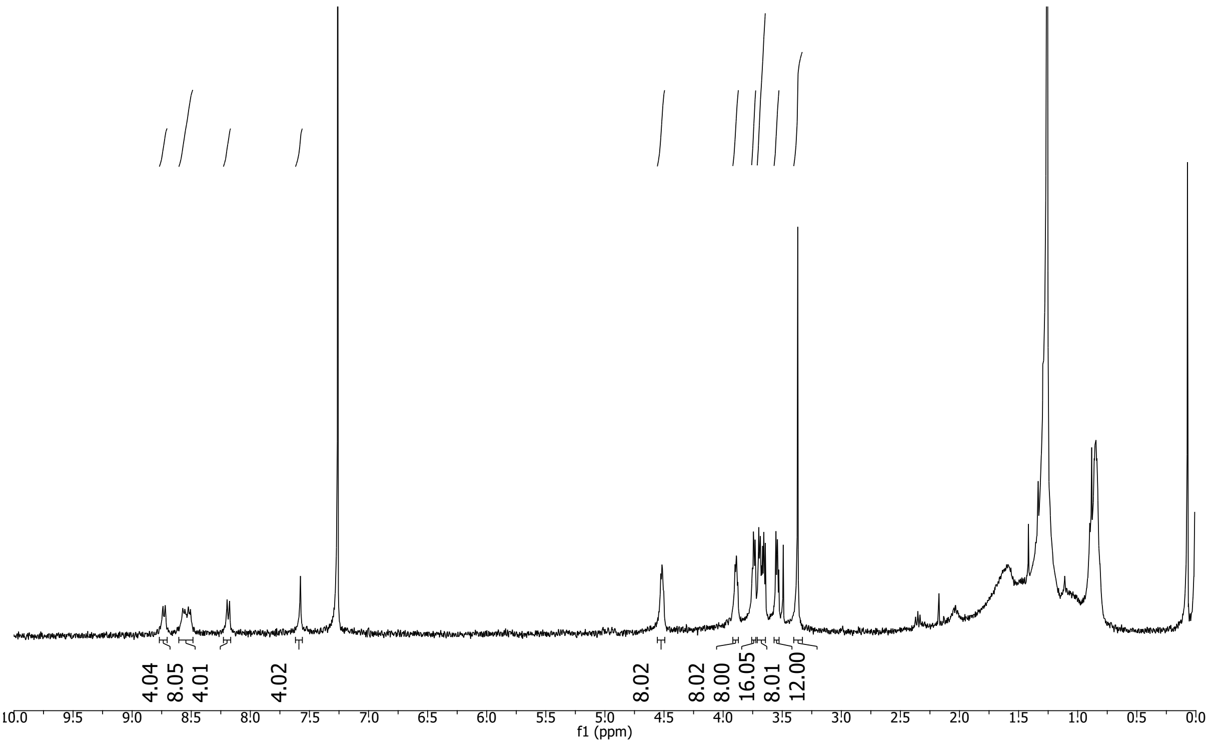

Figure S56. ^{1}H-NMR spectrum (400 MHz, $CDCl_3$, 298 K) of **PMIDE-*p*-d**.

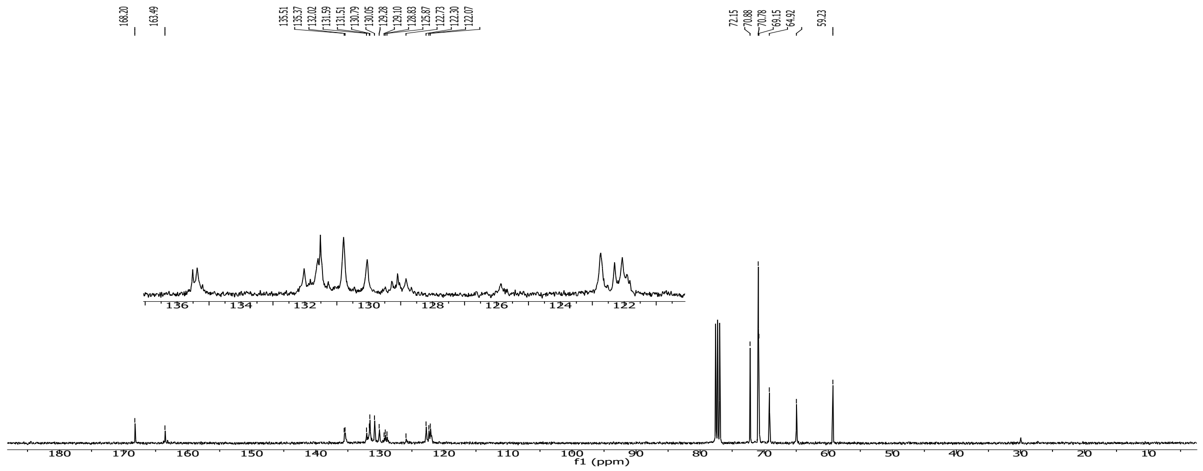

Figure S57. ^{13}C-NMR spektrum (100 MHz, $CDCl_3$, 298 K) of **PMIDE-*p*-d**.

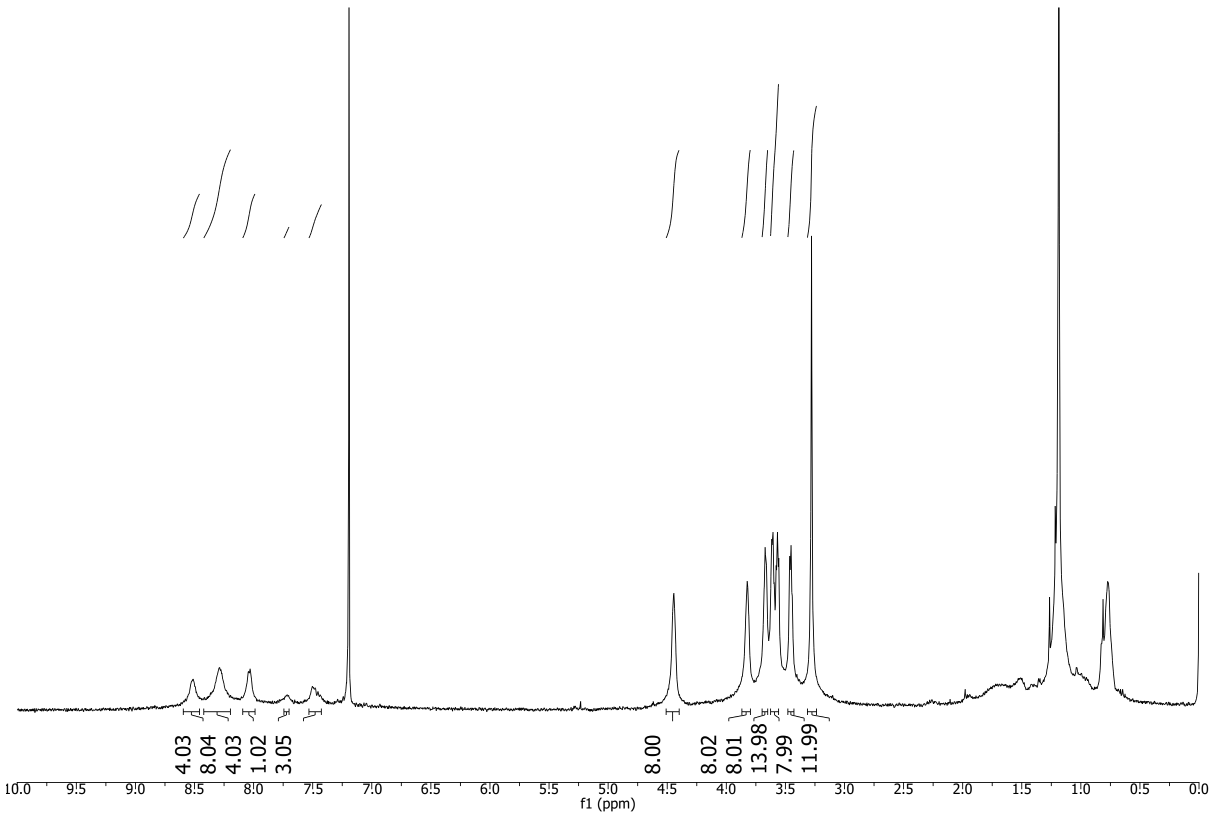

Figure S58. ^{1}H-NMR spectrum (400 MHz, $CDCl_3$, 298 K) of **PMIDE-*m*-d**.

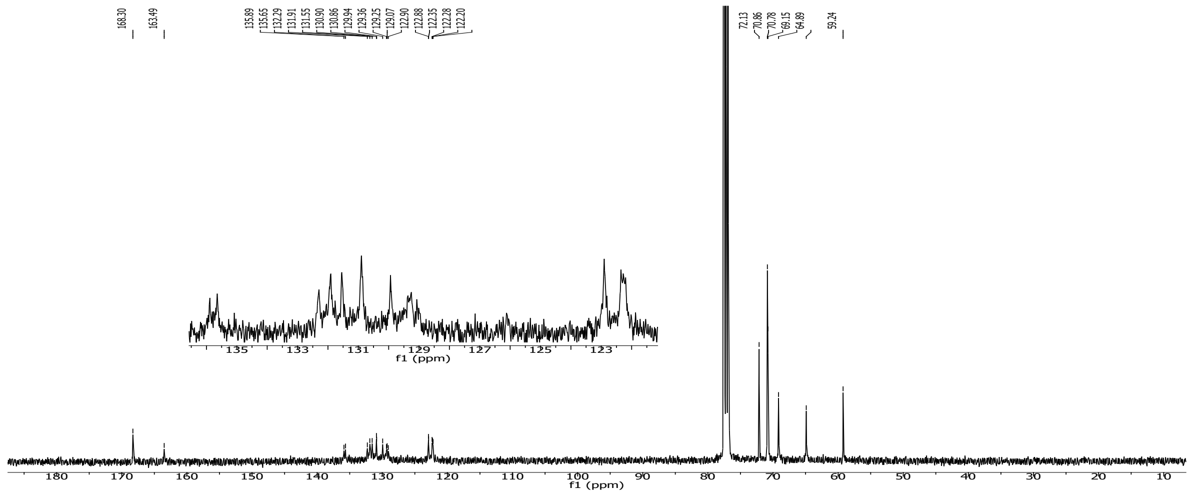

Figure S59. ^{13}C-NMR spectrum (100 MHz, $CDCl_3$, 298 K) of **PMIDE-*m*-d**.

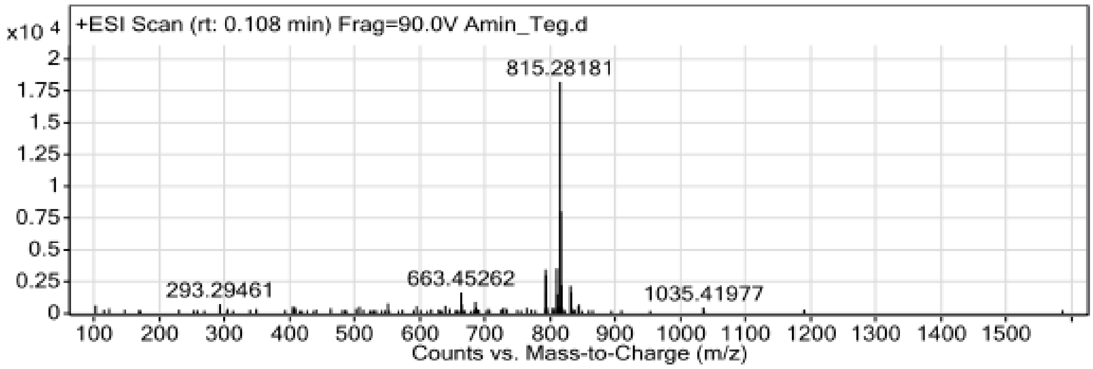

Figure S60. HR-ESI mass spectrum of **PMIDE-m**.

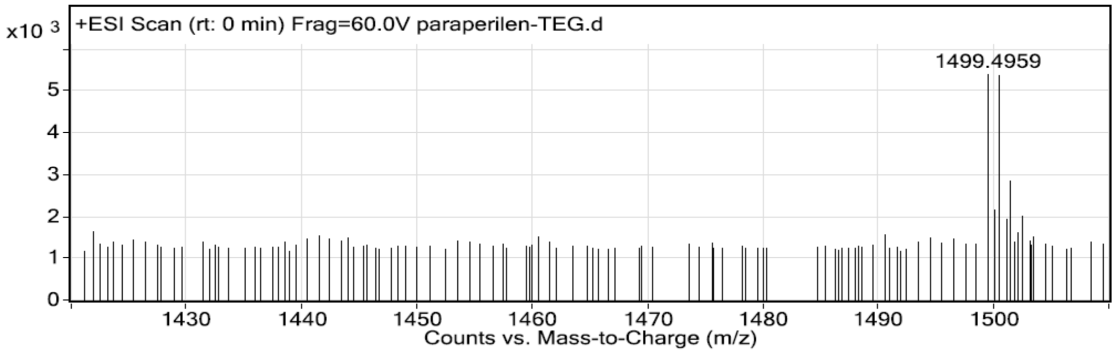

Figure S61. HR-ESI mass spectrum of **PMIDE-*p*-d**.

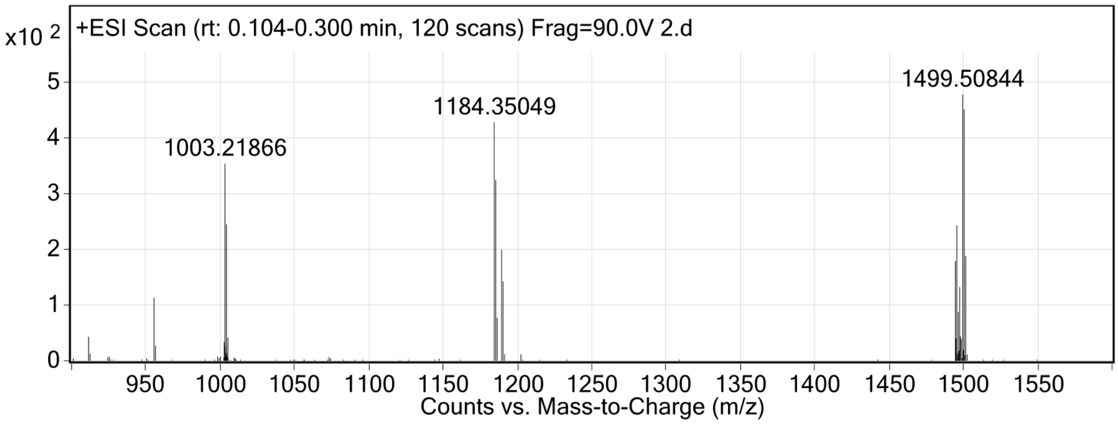

Figure S62. HR-ESI mass spectrum of **PMIDE-*m*-d**.

S10. References

S1. Gültekin, D. D. *J. Photochem. Photobiol. A: Chem.,* **2021**, *426,* 113769.

S2. Marciniak, H.; Auerhammer, N.; Ricker, S.; Schmiedel, A.; Holzapfel, M.; Lambert, C. *J. Phys. Chem. C* **2019**, *123*, 3426-3432.

S3. Porrès, L.; Holland, A.; Pålsson, L.-O.; Monkman, A. P.; Kemp, C.; Beeby, A. *J. Fluoresc*. **2006**, *16*, 267-273.

S4. O'Connor, D. V.; Phillips, D. *Time-Correlated Single Photon Counting*, Academic Press, London, 1984.

S5. Smith, D. A.; McKenzie, G.; Jones, A. C.; Smith, T. A. *Methods Appl. Fluoresc*. **2017**, *5*, 042001.

S6. Benniston, A. C.; Harriman, A.; Li, P. Y.; Rostron, J. P.; van Ramesdonk, H. J.; Groeneveld, M. M.; Zhang, H.; Verhoeven, J. W. *J. Am. Chem. Soc*. **2005**, *127*, 16054-16064.

S7. Schaberle, F. A.; Serpa, C.; Arnaut, L. G. ; Ward, A. D.; Karlsson, J. K. G.; Atahan, A.; Harriman, A. *Chemistry* **2020**, *2*, 545-564.

S8. Polster, J.; Lachmann, H. *Spectrometric Titrations. Analysis of Chemical Equilibria*, VCH, Weinheim, 1989.

S9. (a) Becke, A. D. *J. Chem. Phys*. **1993**, *98*, 5648-5652. (b) Lee, C.; Yang, W.; Parr, R. G. *Phys. Rev. B* **1988**, *37*, 785-789. (c) Vosko, S. H.; Wilk, L.; Nusair, M. *Can. J. Phys*. **1980**, *58*, 1200-1211. (d) Stephens, P. J.; Devlin, F. J.; Chabalowski, C. F.; Frisch, M. J. *J. Phys. Chem*. **1994**, *98*, 11623-11627.

S10. (a) Krishnan, R.; Binkley, J. S.; Seeger, R.; Pople, J. A. *J. Chem. Phys*. **1980**, *72*, 650-654. (b) Clark, T.; Chandrasekhar, J.; Spitznagel, G. W.; Schleyer, P. V. R. *J. Comput. Chem*. **1983**, *4*, 294-301.

S11. Yanai, T.; Tew, D. P.; Handy, N. C. *Chem. Phys. Lett*. **2004**, *393*, 51-57.

S12. Papadopoulos, I.; Gutiérrez-Moreno, D.; McCosker, P. M.; Casillas, R.; Keller, P. A.; Sastre-Santos, À.; Clark, T.; Fernández-Lázaro, F.; Guldi, D. M. *J. Phys. Chem. A* **2020** *124*, 5727-5736.

S13. Frisch, M. J.; Trucks, G. W.; Schlegel, H. B.; Scuseria, G. E.; Robb, M. A.; Cheeseman, J. R.; Scalmani, G.; Barone, V.; Mennucci, B.; Petersson, G. A.; Nakatsuji, H.; Caricato, M.; Li, X.; Hratchian, H. P.; Izmaylov, A. F.; Bloino, J.; Zheng, G.; Sonnenberg, J. L.; Hada, M.; Ehara, M.; Toyota, K.; Fukuda, R.; Hasegawa, J.; Ishida, M.; Nakajima, T.; Honda, Y.; Kitao, O.; Nakai, H.; Vreven, T.; Montgomery, J. A., Jr.; Peralta, J. E.; Ogliaro, F.; Bearpark, M.; Heyd, J. J.; Brothers, E.; Kudin, K. N.; Staroverov, V. N.; Kobayashi, R.; Normand, J.; Raghavachari, K.; Rendell, A.; Burant, J. C.; Iyengar, S. S.; Tomasi, J.; Cossi, M.; Rega, N.; Millam, J. M.; Klene, M.; Knox, J. E.; Cross, J. B.; Bakken, V.; Adamo, C.; Jaramillo, J.; Gomperts, R.; Stratmann, R. E.; Yazyev, O.; Austin, A. J.; Cammi, R.; Pomelli, C.; Ochterski, J. W.; Martin, R. L.; Morokuma, K.; Zakrzewski, V. G.; Voth, G. A.; Salvador, P.; Dannenberg, J. J.; Dapprich, S.; Daniels, A. D.; Farkas, Ö.; Foresman, J. B.; Ortiz, J. V.; Cioslowski, J.; Fox, D. J. Gaussian, Inc., Wallingford CT, 2009. Revision E01.

S14. Martin, R. L. *J. Chem. Phys.* **2003**, *118*, 4775–4777.

S15. Mentel, L. M.; Baerends, E. J. *J. Chem. Theory Comput.* **2014**, *10*, 252–267.

S16. (a) Schmidt, M. W.; Baldridge, K. K.; Boatz, J. A.; Elbert, S. T.; Gordon, M. S.; Jensen, J. H.; Koseki, S.; Matsunaga, N.; Nguyen, K. A.; Su, S.; Windus, T. L.; Dupuis, M.; Montgomery, J. A. *J. Comput. Chem.* **1993**, *14*, 1347-1363. (b) Gordon, M. S.; Schmidt, M. W. in "Theory and Applications of Computational Chemistry: the first forty years" Dykstra, C. E.; Frenking, G.; Kim, K. S.; Scuseria, G. E. (editors), Elsevier, Amsterdam, 2005, pp. 1167-1189.

S17. del Campo, J. M.; Gázquez, J. L.; Trickey, S. B.; Vela, A. *J. Chem. Phys.* **2012**, *136*, 104108.

S18. Mennucci, B.; Tomasi, J.; Cammi, R.; Cheeseman, J. R.; Frisch, M. J.; Devlin, F. J.; Gabriel, S.; Stephens, P. J. *J. Phys. Chem. A* **2002**, *106*, 25, 6102–6113.

S19. Tomasi, J.; Mennucci, B.; Cancès , E. *J. Mol. Struct.: THEOCHEM* **1999**, *464*, 211-226.
